# Multi Component Reactions under Increased Pressure: On the Mechanism of Formation of Pyridazino[5,4,3-*de*][1,6]naphthyridine Derivatives by the Reaction of Malononitrile, Aldehydes and 2-Oxoglyoxalarylhydrazones in Q-Tubes

**DOI:** 10.3390/molecules22122114

**Published:** 2017-12-01

**Authors:** Majdah A. AL-Johani, Khadijah M. Al-Zaydi, Sameera M. Mousally, Norah F. Alqahtani, Noha Hilmy Elnagdi, Mohamed H. Elnagdi

**Affiliations:** 1 Department of Chemistry, Faculty of Sciences—AL Faisaliah, King Abdulaziz University, Jeddah, P.O. Box 50918, Jeddah 21533, Saudi Arabia; moon98.1@hotmail.com (M.A.A.); smousally@kau.edu.sa (S.M.M.); Nalqahtani00123@kau.edu.sa (N.F.A.); 2 Department of Organic Chemistry, Faculty of Pharmacy, Modern University for Technology and Information, Cairo, P.O. Box 12518, Cairo 11511, Egypt; elnagdinoha@yahoo.com; 3 Faculty of Science, Cairo University, Cairo, P.O. Box 12613, Cairo 11511, Egypt; m.h.elnagdi@outlook.com

**Keywords:** X-ray crystallography, arylhydrazonals, 2-amino-1,1,3-propenetricarbonitrile, pyridazines, negative activation volume

## Abstract

Efficient synthesis of phenanthridin-6(5*H*)-one derivatives **12a**–**n** in a four-component reaction of aldehyde hydrazone, aromatic aldehydes and malononitrile in Q-Tubes is reported. The results showed that the methodology has the advantage of being a one-pot synthesis of tricyclic systems in good yields. Potential routes leading to formation of compounds **12** are discussed. The structures of the synthesized compounds could be unequivocally established via X-ray crystal structure determination and spectroscopic methods.

## 1. Introduction

**Scheme 1:**
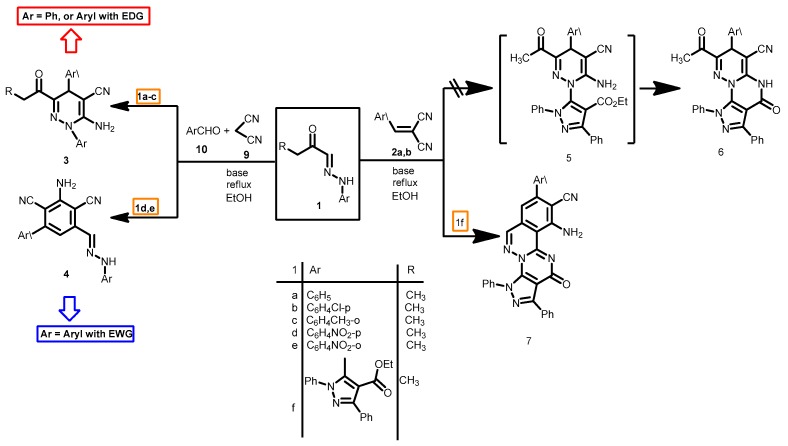
The reactivity of aryl hydrazones **1** towards α,β-functionally substituted cinnamonitriles.

The considerable biological and medicinal activities of pyridazines has stimulated considerable research on efficient syntheses of these derivatives in past years [1,2,3,4]. Elnagdi et al. reported synthesis of 2-amino-1,4-dihydropyridazine, an isoelectronic derivative of 1,4-dihydropyrimidines of established biological activities [5,6,7,8], via 3 + 3 atom combination of arylhydrazones **1a** and α,β-unsaturated nitriles **2** [9] or by reacting a mixture of **1**, **4** and **5** in one pot (Scheme 1). Subsequent studies [10,11] on this novel route revealed however that it is of a limited scope as the reaction products proved to be dependent on the nature of the reacting aryl hydrazones. Multicomponent reaction of **1a**–**c** with α,β-unsaturated nitriles **2** in presence of a base has been reported to yield **3**, while the reaction of **1f** with **2** in basic medium afforded the new substituted pyrazolo[4′,3′-5,6]pyrimido[2,1-a]phthalazine-9-carbonitriles ring system **7** [12] (Scheme 1).

Microwave energy has been reported to be effective in the synthesis of small molecules in many of our previous works [13,14,15,16]. However, we noted that microwaves technology is expensive to scale up [17], in contrast to using “Q-Tube” pressure reactors, which proved to accelerate reactions of negative activation volume in a more optimal and safer manner, compared to microwaves [18]. 

**Scheme 2:**
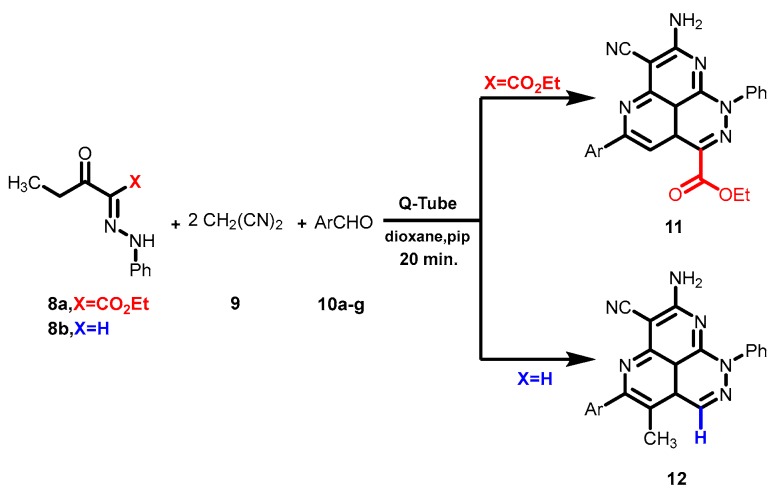
Novel synthesis of the tricyclic system **11** by reacting ethyl-3-oxo-2-(2-phenylhydrazono) pentanoate (**8a**) with malononitrile (**9**) and aromatic aldehyde derivatives **10** in a Q-Tube.

Our research group has previously reported extensively on the use of Q-Tubes to synthesize such compounds but the reaction conditions in these published works had many limitations that altered the nature of the synthesized products [19,20] (Scheme 2). The human urge to find cures for challenging diseases and improve human life leads organic chemists to be in a continuous quest to develop novel polyfunctional heterocycles, as well as developing new economical and greener technologies. Considering the promising biological activity of new compounds **12** where the ring system combines pyridazine and napthyridine rings, both with vast biological activities [21,22,23,24], we sought to expand this work to prove that the method proposed for the synthesis of these novel compounds is a general one by making more examples. Moreover this work has led to the proposal of a plausible reaction mechanism that would clarify and lead the way for any further work on such new ring systems.

## 2. Results

**Scheme 3:**
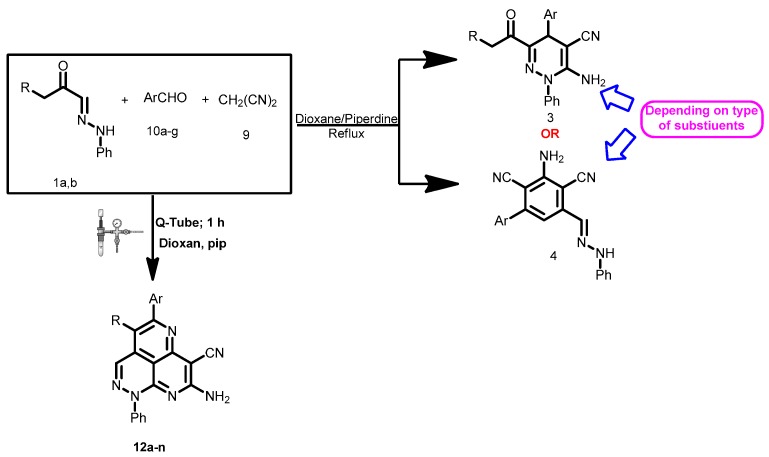
A new reaction path for the reaction of aldehyde hydrazones with malononitrile and aldehydes.

**Figure 1:**
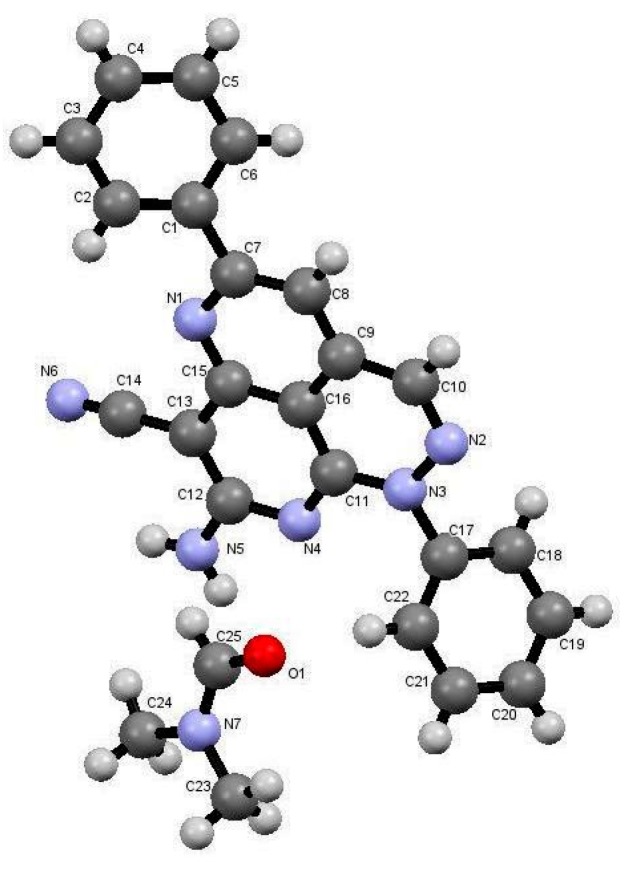
X-ray crystallographic structure of compound **12a** [25].

**Figure 2:**
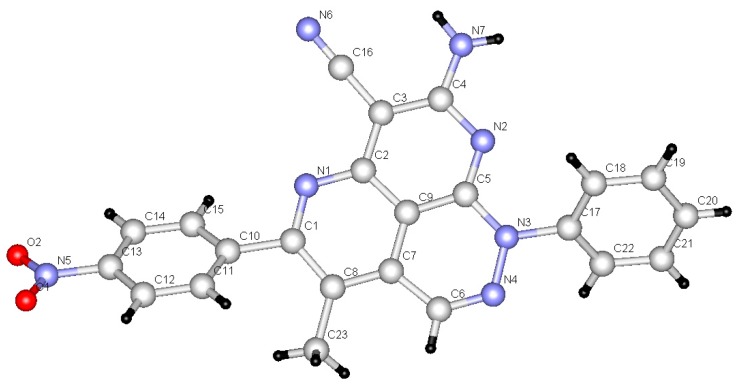
X-ray crystallographic structure of compound **12m** [26].

**Figure 3:**
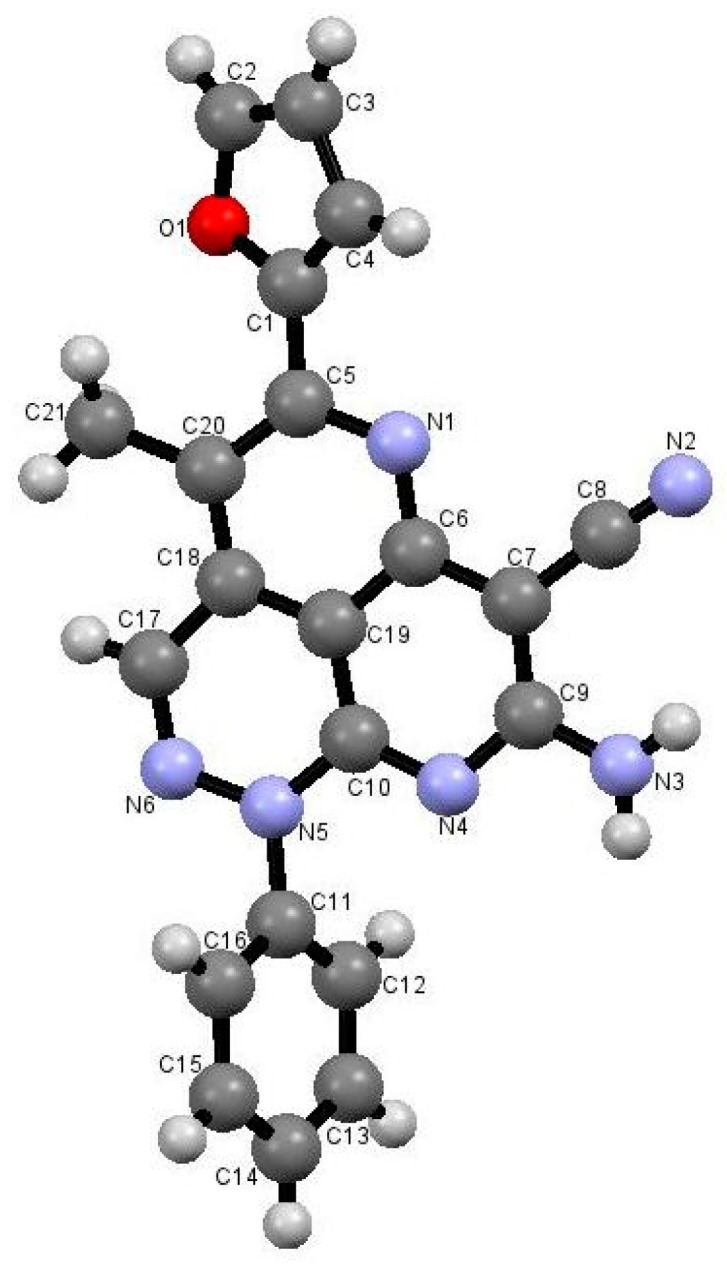
X-ray crystallographic structure of compound **12n** [27].

The reactions in Q-Tubes (cf. Figure 1) at 150 °C and 20 psi of 2-oxo-2-arylhydrazonals **1a**,**b** with aromatic aldehydes **10a**–**g** and malononitrile (**9**) in dioxane in the presence of piperidine afforded compounds **12a**–**n**. The tricyclic systems **12** are formed in 72–85% yield (Scheme 3, Table 1). The structure of the reaction products could be established to be pyridazino[5,4,3-*de*][1,6]naphthyridine derivatives **12a**–**n** via spectroscopic methods (available in the supplementary materials) as well as X-ray crystal structure determination of products **12a**, **12m** and **12n** (Figure 1, Figure 2 and Figure 3).

## 3. Discussion

**Scheme 4:**
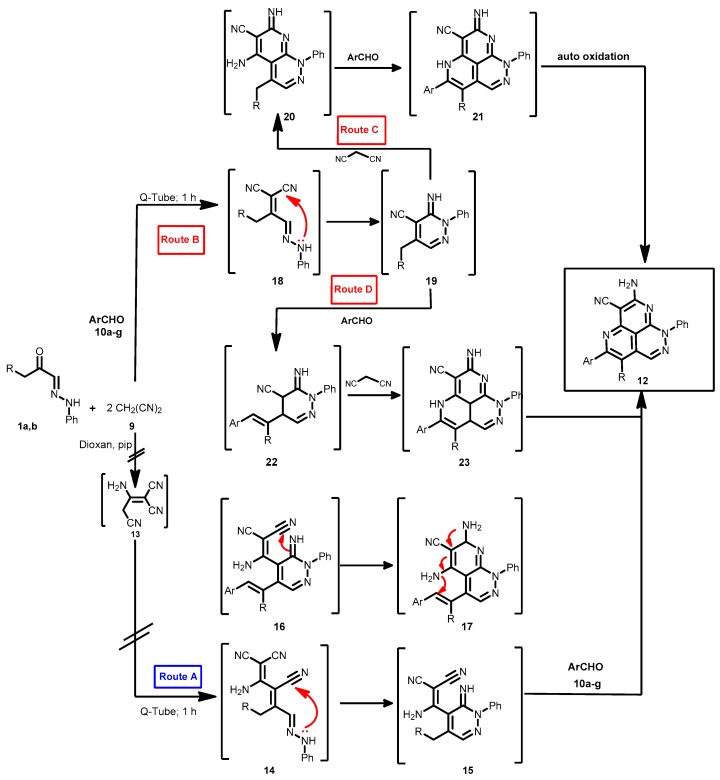
The proposed mechanism for the formation of pyridazino[5,4,3-*de*][1,6]naphthyridine derivatives **12a**–**n**.

Two mechanistic pathways seem possible (Scheme 4): initial dimerization of malononitrile to yield dimer **13**, that then condenses with the acyl carbonyl yielding **14** that cyclizes to form **15** (route A) [19]. This route could readily be eliminated as in our hands malononitrile could not be dimerized under the reported conditions moreover when it is considered that this dimerization it probably impossible in the absence of ethoxide or sodium hydroxide which are thought necessary for the dimerization of malononitrile [27].

Moustafa et al. also reported that the dimer **13** alone reacts with their arylhydrazones **1a**,**b** yielding pyridazino[5,4,3-*de*][1,6]naphthyridine derivatives, not condensed with pyridazine derivatives. Thus, it is almost certain that the initial step leading to formation of **12** is the condensation of malononitrile (**9**) with acyl carbonyl **18**. The product **19** can then either cyclize into **20** and then **21** (route C) or condense with an aromatic aldehyde to give **22** and then **23** (route D). Neither route C or D can completely be ruled out, although we believe that the aromatic aldehyde condenses initially with **19** then subsequent reactions lead to **22** that then reacts with malononitrile (**9**) to form **23** which cyclizes to the final product **12** (Scheme 4).

## 4. Materials and Methods

### 4.1. General Information

Q-tube assisted reactions were performed in a Q-tube safe pressure reactor from Q Labtech (East Lyme, CT 06333, CT, USA, equipped with a cap/sleeve, pressure adapter (120 psi), needle adapter/needle, borosilicate glass tube, Teflon septum, and catch bottle. All reactions were monitored by using TLC with 1:1 ethyl acetate-petroleum ether as eluent and were carried out until starting materials were completely consumed. Melting points are reported uncorrected and were determined with a Sanyo (Gallenkamp, Osaka, Japan) instrument. Infrared spectra were recorded using KBr pellets and a FT–IR 6300 instrument (Jasco, Tokyo, Japan) and absorption bands are reported in cm^−1^. ^1^H- and ^13^C-NMR spectra were determined by using a DPX instrument (Bruker, Billerica, MA, USA) at 400 MHz or 600 MHz for ^1^H-NMR and 100 MHz for ^13^C-NMR and either CDCl_3_ or DMSO-*d*_6_ solutions with TMS as internal standards. Chemical shifts are reported in ppm. Mass spectra and accurate mass measurements were made using a GCMS DFS spectrometer (Thermo, Bremen, Germany) with the EI (70 EV) mode. X-ray crystallographic structure determinations were performed by using Rapid II (Rigaku, Tokyo, Japan) and X8 Prospector (Bruker, Karlsruhe, Germany) single crystal X-ray diffractometers. All X-ray crystal structure data can be obtained free of charge from the Cambridge Crystallographic Data Centre [26,27,28] via www.ccdc.cam.ac.uk. The data and material are available in the Supplementary material and manuscript. Supplementary material is attached as PDF format and submitted along with the manuscript.

### 4.2. General Procedures for Q-Tube-Assisted Synthesis of ***12a–n***

2-Oxo-2-arylhydrazonals **1a**,**b** (0.01 mol), aromatic aldehydes **13a**–**g** (0.01 mol) and malononitrile (**9**) was 9 before (0.02 mol) in the presence of piperidine (1 mL) and dioxin (20 mL) as solvent were sequentially added in a 35 mL Q-tube pressure tube, furnished by Q Labtech. A Teflon septum was placed on top of the tube, and an appropriate cap was used. The mixture was heated in an oil bath at 150 °C. After about 60 min, the reaction mixture was monitored by TLC. The mixture was cooled and poured into ice-water. The solid was collected by filtration and purified by column chromatography and crystallized from ethanol. 

*8-Amino-1,5-diphenyl-1H-pyridazino[5,4,3-de][1,6]naphthyridine-7-carbonitrile* (**12a**). Dark yellow crystals, Yield 85%; m.p. 314–315 °C; Anal. Calcd. for C_22_H_14_N_6_ (362.13): C, 72.92; H, 3.89; N, 23.19. Found: C, 72.83; H, 3.79; N, 23.25. EI-HRMS: *m*/*z* = 362.1274 (MH^+^); C_22_H_14_N_6_ requires: *m*/*z* = 362.1279 (MH^+^); ^1^H-NMR (400 MHz, DMSO-*d*_6_): δ = 7.06 (br, 2H, NH_2_, D_2_O exchangeable), 7.44–7.66 (m, H, Ph-H, CH), 8.19–8.22 (m, 2H, Ph-H), 8.38 (s, 1H, CH); ^13^C-NMR (100 MHz, DMSO-*d*_6_): δ = 162.0, 161.2, 154.5, 150.9, 141.7, 138.7, 137.8, 133.2, 130.3, 128.8 (2C), 128.7 (2C), 128.1, 127.1 (2C), 126.3 (2C), 116.8, 108.8, 104.6, 73.3. MS: *m*/*z* (%) 362.2 (M^+^, 100), 334 (10), 181 (10), 77 (5).

*8-Amino-5-(4-chlorophenyl)-1-phenyl-1H-pyridazino[5,4,3-de][1,6]naphthyridine-7-carbonitrile* (**12b**). Green crystals, yield 75%; m.p. 340–341 °C; Anal. Calcd. for C_22_H_13_ClN_6_ (396.06): C, 66.59; H, 3.30; N, 21.18. Found: C, 66.70; H, 3.35; N, 21.20. EI-HRMS: *m*/*z* = 396.0885 (MH^+^); C_22_H_13_N_6_^35^Cl requires: *m*/*z* 396.0890 (MH^+^); ^1^H-NMR (400 MHz, DMSO-*d*_6_): δ = 7.10 (br, 2H, NH_2_, D_2_O exchangeable), 7.45–7.66 (m, 8H, Ph-H, CH), 8.22–8.24 (m, 2H, Ph-CH), 8.38 (s, 1H, CH); ^13^C-NMR (100 MHz, DMSO-*d*_6_): δ = 162.1, 160.0, 154.6, 151.0, 141.7, 138.7, 136.7, 133.4, 130.3, 129.1 (2C), 128.9 (2C), 128.2, 126.6 (2C), 124.6 (2C), 118.0, 109.1, 104.6, 56.0. MS: *m*/*z* (%) 396.1 (M^+^, 100), 368 (10), 198 (10), 166 (5), 77 (5).

*8-Amino-5-(3-chlorophenyl)-1-phenyl-1H-pyridazino[5,4,3-de][1,6]naphthyridine-7-carbonitrile* (**12c**). Green crystals, yield 80%; m.p. 314–316 °C; Anal. Calcd. for C_22_H_13_ClN_6_ (396.06): C, 66.59; H, 3.30; N, 21.18. Found: C, 66.57; H, 3.33; N, 21.12. EI-HRMS: *m*/*z* = 396.0885 (MH^+^); C_22_H_13_N_6_^35^Cl requires: *m*/*z* = 396.0890 (MH^+^); ^1^H-NMR (400 MHz, DMSO-*d*_6_): δ = 7.11 (br, 2H, NH_2_, D_2_O exchangeable), 7.27 (s, 1H, CH), 7.45–7.67 (m, 9H, Ph-H), 8.45 (s, 1H, CH); ^13^C-NMR (100 MHz, DMSO-*d*_6_): δ = 162.2, 162.0, 154.6, 151.1, 141.7, 138.8, 138.5, 132.4, 131.4, 131.0, 130.6, 129.9 (2C), 128.8, 128.2, 127.4, 126.4 (2C), 116.8, 108.9, 108.8, 73.1. MS: *m*/*z* (%) 396.1 (M^+^, 100), 361 (15), 334 (5), 198 (10), 166 (5), 77 (5).

*8-Amino-1-phenyl-5-p-tolyl-1H-pyridazino[5,4,3-de][1,6]naphthyridine-7-carbonitrile* (**12d**). Faint green crystals, yield 77%; m.p. 340–341 °C; Anal. Calcd. for C_23_H_16_N_6_ (376.14): C, 73.39; H, 4.28; N, 22.33. Found: C, 73.34; H, 4.30; N, 22.28. EI-HRMS: *m*/*z* = 376.1430 (MH^+^); C_23_H_16_N_6_ requires: *m*/*z* = 376.1436 (MH^+^); ^1^H-NMR (400 MHz, DMSO-*d*_6_): δ = 2.39, 2.51 (s, 3H, CH_3_), 7.05 (br, 2H, NH_2_, D_2_O exchangeable), 7.34–7.65 (m, 8H, Ph-H, CH), 8.09–8.11 (m, 2H, Ph-H), 8.36 (s, 1H, CH); ^13^C-NMR (100 MHz, DMSO-*d*_6_): δ = 162.2, 161.2, 154.4, 150.9, 141.7, 140.2, 138.8, 135.1, 133.2, 129.4 (2C), 128.8 (2C), 128.1, 127.1 (2C), 126.3 (2C), 116.9, 108.7, 104.2, 73.3, 20.9. MS: *m*/*z* (%) 376.2 (M^+^, 100), 348 (10), 188 (10), 77 (5).

*8-Amino-1-phenyl-5-m-tolyl-1H-pyridazino[5,4,3-de][1,6]naphthyridine-7-carbonitrile* (**12e**). Green crystals, yield 73%; m.p. 280–281 °C; Anal. Calcd. for C_23_H_16_N_6_ (376.14): C, 73.39; H, 4.28; N, 22.33. Found: C, 73.38; H, 4.27; N, 22.31. EI-HRMS: *m*/*z* = 376.1431 (MH^+^); C_23_H_16_N_6_ requires: *m*/*z* = 376.1436 (MH^+^); ^1^H-NMR (400 MHz, DMSO-*d*_6_): δ = 2.43,2.51 (s, 3H, CH_3_), 7.07 (br, 2H, NH_2_, D_2_O exchangeable), 7.18 (s, 1H, CH), 7.34–7.66 (m, 9H, Ph-H), 8.40 (s, 1H, CH); ^13^C-NMR (100 MHz, DMSO-*d*_6_): δ = 165.2, 162.0, 154.3, 151.1, 141.7, 139.6, 138.7, 135.8, 133.7, 130.8, 129.3, 128.9, 128.2 (2C), 128.1, 126.3 (2C), 125.9, 116.9, 108.3, 108.2, 73.3, 20.3. MS: *m*/*z* (%) 376 (M^+^, 50), 375 (100), 348 (10), 255 (10), 187 (10), 77 (5).

*8-Amino-5-(4-nitrophenyl)-1-phenyl-1H-pyridazino[5,4,3-de][1,6]naphthyridine-7-carbonitrile* (**12f**). Dark brown crystals, yield 82%; m.p. 397–398 °C; Anal. Calcd. for C_22_H_13_N_7_O_2_ (407.11): C, 64.86; H, 3.22; N, 24.07. Found: C, 64.75; H, 3.10; N, 24.12. EI-HRMS: *m*/*z* = 407.1125 (MH^+^); C_22_H_13_O_2_N_7_ requires: *m*/*z* = 407.1131 (MH^+^); ^1^H-NMR (400 MHz, DMSO-*d*_6_): δ = 7.18 (br, 2H, NH_2_, D_2_O exchangeable), 7.48–7.68 (m, 5H, Ph-H, CH), 7.93, 8.244 (d, 1H, CH-pyridazine), 8.40–8.48 (m, 4H, Ph-H, CH), 9.20, 9.41 (s, 1H, CH). MS: *m*/*z* (%) 407.2 (M^+^, 100), 361 (20), 334 (10), 180 (10), 77 (5).

*8-Amino-5-(furan-2-yl)-1-phenyl-1H-pyridazino[5,4,3-de][1,6]naphthyridine-7-carbonitrile* (**12g**). Dark green crystals, yield 86%; m.p. 345–346 °C; Anal. Calcd. for C_20_H_12_N_6_O (352.11): C, 68.18; H, 3.43; N, 23.85. Found: C, 68.21; H, 3.52; N, 23.77. EI-HRMS: *m*/*z* = 352.1066 (MH^+^); C_20_H_12_O_1_N_6_ requires: *m*/*z* = 352.1072 (MH^+^); ^1^H-NMR (400 MHz, DMSO-*d*_6_): δ = 6.74–6.75 (m, 1H, furyl-H), 7.05 (br, 2H, NH_2_, D_2_O exchangeable), 7.24–7.97 (m, 8H, Ph-H, furyl-H, CH), 8.42 (s, 1H, CH); ^13^C-NMR (100 MHz, DMSO-*d*_6_): δ = 162.0, 154.6, 153.2, 152.7, 150.8, 145.6, 141.7, 138.6, 133.2, 128.8 (2C), 128.2, 126.3 (2C), 116.8, 112.7, 11.8, 108.6, 102.7, 73.0. MS: *m*/*z* (%) 352.1 (M^+^, 100), 324 (5), 176 (10), 77 (5).

*8-Amino-4-methyl-1,5-diphenyl-1H-pyridazino[5,4,3-de][1,6]naphthyridine-7-carbonitrile* (**12h**). Yellow crystals, yield 83%; m.p. 364–365 °C; Anal. Calcd. for C_23_H_16_N_6_ (376.14): C, 73.39; H, 4.28; N, 22.33. Found: C, 73.35; H, 4.15; N, 22.41. EI-HRMS: *m*/*z* = 376.1431 (MH^+^); C_23_H_16_N_6_ requires: *m*/*z* = 376.1436 (MH^+^); ^1^H-NMR (400 MHz, DMSO-*d*_6_): δ = 2.34, 2.50 (s, 3H, CH_3_), 6.95 (br, 2H, NH_2_, D_2_O exchangeable), 7.45–7.66 (m, 10H, Ph-H, CH), 8.56 (s, 1H, CH); ^13^C-NMR (100 MHz, DMSO-*d*_6_): δ = 164.5, 161.6, 152.3, 150.6, 141.8, 140.0, 136.9, 131.0, 129.0 (2C), 128.8 (2C), 128.5, 128.2, 128.0 (2C), 126.3 (2C), 117.1, 114.1, 108.9, 72.6, 13.9. MS: *m*/*z* (%) 376.2 (M^+^, 100), 368 (10), 348 (5), 255 (5), 188 (10), 97 (10), 57 (5).

*8-Amino-5-(4-chlorophenyl)-4-methyl-1-phenyl-1H-pyridazino[5,4,3-de][1,6]naphthyridine-7-carbonitrile* (**12j**). Dark yellow crystals, yield 78%; m.p. 345–346 °C; Anal. Calcd. for C_23_H_15_ClN_6_ (410.1): C, 67.24; H, 3.68; N, 20.45. Found: C, 67.27; H, 3.56; N, 20.45. EI-HRMS: *m*/*z* = 410.1041 (MH^+^); C_23_H_15_N_6_^35^Cl requires: *m*/*z* = 410.1047 (MH^+^); ^1^H-NMR (400 MHz, DMSO-*d*_6_): δ = 2.35, 2.50 (s, 3H, CH_3_), 6.90 (br, 2H, NH_2_, D_2_O exchangeable), 7.45–7.66 (m, 9H, Ph-H, CH), 8.58 (s, 1H, CH); ^13^C-NMR (100 MHz, DMSO-*d*_6_): δ = 163.8, 162.1, 156.4, 151.1, 142.3, 139.4, 137.3, 134.0, 132.5, 131.7, 131.3 (2C), 129.3 (2C), 128.6 (2C), 126.7 (2C), 117.2, 114.8, 109.7, 73.5, 14.3. MS: *m*/*z* (%) 410.1 (M^+^, 100), 374 (10), 346 (5), 255 (5), 205 (5), 187 (10), 173 (5), 97 (5), 77 (5).

*8-Amino-4-methyl-1-phenyl-5-p-tolyl-1H-pyridazino[5,4,3-de][1,6]naphthyridine-7-carbonitrile* (**12k**). Dark orange crystals, yield 80%; m.p. 370–371 °C; Anal. Calcd. for C_24_H_18_N_6_ (390.16): C, 73.83; H, 4.65; N, 21.52. Found: C, 73.88; H, 4.59; N, 21.60. EI-HRMS: *m*/*z* = 390.1587 (MH^+^); C_24_H_18_N_6_ requires: *m*/*z* = 390.1593 (MH^+^); ^1^H-NMR (400 MHz, DMSO-*d*_6_): δ = 2.36 (s, 3H, CH_3_), 2.41 (s, 3H, CH_3_), 6.93 (br, 2H, NH_2_, D_2_O exchangeable), 7.34-7.67 (m, 9H, Ph-H, CH), 8.56 (s, 1H, CH); ^13^C-NMR (100 MHz, DMSO-*d*_6_): δ = 163.9, 161.6, 152.4, 150.7, 141.8, 140.2, 137.2, 129.0 (2C), 128.8 (2C), 128.6 (2C), 127.1, 126.3 (2C), 129.1, 124.5, 119.1, 114.8, 109.7, 72.6, 20.8, 14.0. MS: *m*/*z* (%) 390.2 (M^+^, 100), 375 (5), 269 (5), 187 (5), 77 (5).

*8-Amino-4-methyl-1-phenyl-5-m-tolyl-1H-pyridazino[5,4,3-de][1,6]naphthyridine-7-carbonitrile* (**12l**). Yellow crystals, yield 72%; m.p. 284–285 °C; Anal. Calcd. for C_24_H_18_N_6_ (390.16): C, 73.83; H, 4.65; N, 21.52. Found: C, 73.81; H, 4.68; N, 21.55. EI-HRMS: *m*/*z* = 390.1587 (MH^+^); C_24_H_18_N_6_ requires: *m*/*z* = 390.1592 (MH^+^); IR: 3489, 3336 (NH_2_), 2200 (CN); ^1^H-NMR (400 MHz, DMSO-*d*_6_): δ = 2.08 (s, 3H, CH_3_), 2.11 (s, 3H, CH_3_), 6.96 (br, 2H, NH_2_, D_2_O exchangeable), 7.19–7.66 (m, 9H, Ph-H, CH), 8.53 (s, 1H, CH); ^13^C-NMR (100 MHz, DMSO-*d*_6_): δ = 165.5, 161.5, 152.4, 150.7, 141.8, 139.8, 136.9, 134.8, 130.6, 130.0, 128.9 (2C), 128.2 (2C), 126.3 (2C), 125.6 (2C), 117.1, 114.7, 109.0, 72.6, 19.0, 13.0. MS: *m*/*z* (%) 390.2 (M^+^, 50), 375 (100), 346 (5), 255 (5), 195 (5), 187 (15), 173 (10), 129 (5), 77 (5).

*8-Amino-4-methyl-5-(4-nitrophenyl)-1-phenyl-1H-pyridazino[5,4,3-de][1,6]naphthyridine-7-carbonitrile* (**12m**). Dark yellow crystals, yield 82%; m.p. 368–369 °C; Anal. Calcd. for C_23_H_15_N_7_O_2_ (421.13): C, 65.55; H, 3.59; N, 23.27. Found: C, 65.59; H, 3.63; N, 23.31. EI-HRMS: *m*/*z* = 421.1282 (MH^+^); C_23_H_15_O_2_N_7_ requires: *m*/*z* = 421.1287 (MH^+^); ^1^H-NMR (400 MHz, DMSO-*d*_6_): δ = 2.37, 2.53 (s, 3H, CH_3_), 6.82 (br, 2H, NH_2_, D_2_O exchangeable), 7.47–8.38 (m, 9H, Ph-H, CH), 8.58, 8.58 (s, 1H, CH); ^13^C-NMR (100 MHz, DMSO-*d*_6_): δ = 14.56, 49.5, 105, 124.12 (2C) , 127.10 (2C), 129.08, 129.70 (2C), 131.31 (4C), 135.61, 137.67, 142.69, 162.56, 163.25; MS: *m*/*z* (%) 421.2 (M^+^, 100), 390 (15), 374 (25), 348 (10), 255 (5), 187 (10), 77 (5).

*8-Amino-5-(furan-2-yl)-4-methyl-1-phenyl-1H-pyridazino[5,4,3-de][1,6]naphthyridine-7-carbonitrile* (**12n**). Dark green crystals, yield 86%; m.p. 368–369 °C; Anal. Calcd. for C_21_H_14_N_6_O (366.12): C, 68.84; H, 3.85; N, 22.94. Found: C, 68.89; H, 3.78; N, 22.88. EI-HRMS: *m*/*z* = 366.1223 (MH^+^); C_21_H_14_O_1_N_6_ requires: *m*/*z* = 366.1229 (MH^+^); ^1^H-NMR (400 MHz, DMSO-*d*_6_): δ = 2.50 (s, 3H, CH_3_), 6.72–6.73 (m, 1H, furyl-H), 6.91 (br, 2H, NH_2_, D_2_O exchangeable), 7.19–7.97 (m, 7H, Ph-H, furyl-H), 8.52 (s, 1H, CH); ^13^C-NMR (100 MHz, DMSO-*d*_6_): δ = 161.6, 153.0, 152.4, 152.2, 150.4, 145.1, 141.8, 136.8, 131.8, 128.9 (2C), 128.2, 126.3 (2C), 117.0, 114.3, 113.2, 112.0, 108.8, 72.3, 13.2. MS: *m*/*z* (%) 366.1 (M^+^, 100), 337 (5), 311 (5), 183 (5), 77 (10).

## 5. Conclusions

Synthesis of 2-amino-1,4-dihydropyridazines by reacting arylhydrazonals, active methylene nitriles and aromatic aldehydes has been found of very limited scope. We show here that under pressure the sequence of this multicomponent reaction changes as the initial step with the least activation volume predominates, and in this way a novel route to pyridazino[5,4,3-*de*][1,6]naphthyridines could be developed. Our observations open the route for discovering new multicomponent reactions under pressure, thus it is recommended to expand this technique.

## Figures and Tables

**Table 1:** Synthesis of pyridazino[5,4,3-*de*][1,6]naphthyridine derivatives **12a**–**n**.

Entry	R	Ar	Yield %	Time (min)
12a	H	Ph	85	60
12b	H	4-ClC_6_H_4_	75	60
12c	H	2-ClC_6_H_4_	80	120
12d	H	4-CH_3_C_6_H_4_	77	60
12e	H	2-CH_3_C_6_H_4_	73	120
12f	H	4-O_2_NC_6_H_4_	82	60
12g	H	2-furyl	86	120
12h	CH_3_	Ph	83	60
12j	CH_3_	4-ClC_6_H_4_	78	60
12k	CH_3_	4-CH_3_C_6_H_4_	80	60
12l	CH_3_	2-CH_3_C_6_H_4_	72	120
12m	CH_3_	4-O_2_NC_6_H_4_	82	60
12n	CH_3_	2-furyl	86	120

## Supplementary Materials

Supplementary Materials are available online.
