# Supplementary material for: Multi Component Reactions under Increased Pressure: On the Mechanism of Formation of Pyridazino[5,4,3-de][1,6]naphthyridine Derivatives by the Reaction of Malononitrile, Aldehydes and 2-Oxoglyoxalarylhydrazones in Q-Tubes
Source: Molecules. 2017 Dec 1;22(12):2114. doi: 10.3390/molecules22122114 (PMC6149974; doi:10.3390/molecules22122114)

(Supplementary material)

Multi component reactions under increased pressure: On the mechanism of formation of pyridazino[5,4,3-de][1,6]naphthyridine derivatives from reaction of malononitrile, aldehyde and 2-oxoglyoxalarylhydrazones in Q-tubes

Majdah A. AL-Johani [1], Khadijah M. Al-Zaydi [1,*], Sameera M. Mousally [1], Norah F. Alqahtani [1], Noha Hilmy Elnagdi [2], Mohamed H. Elnagdi [3]

Compound 12a

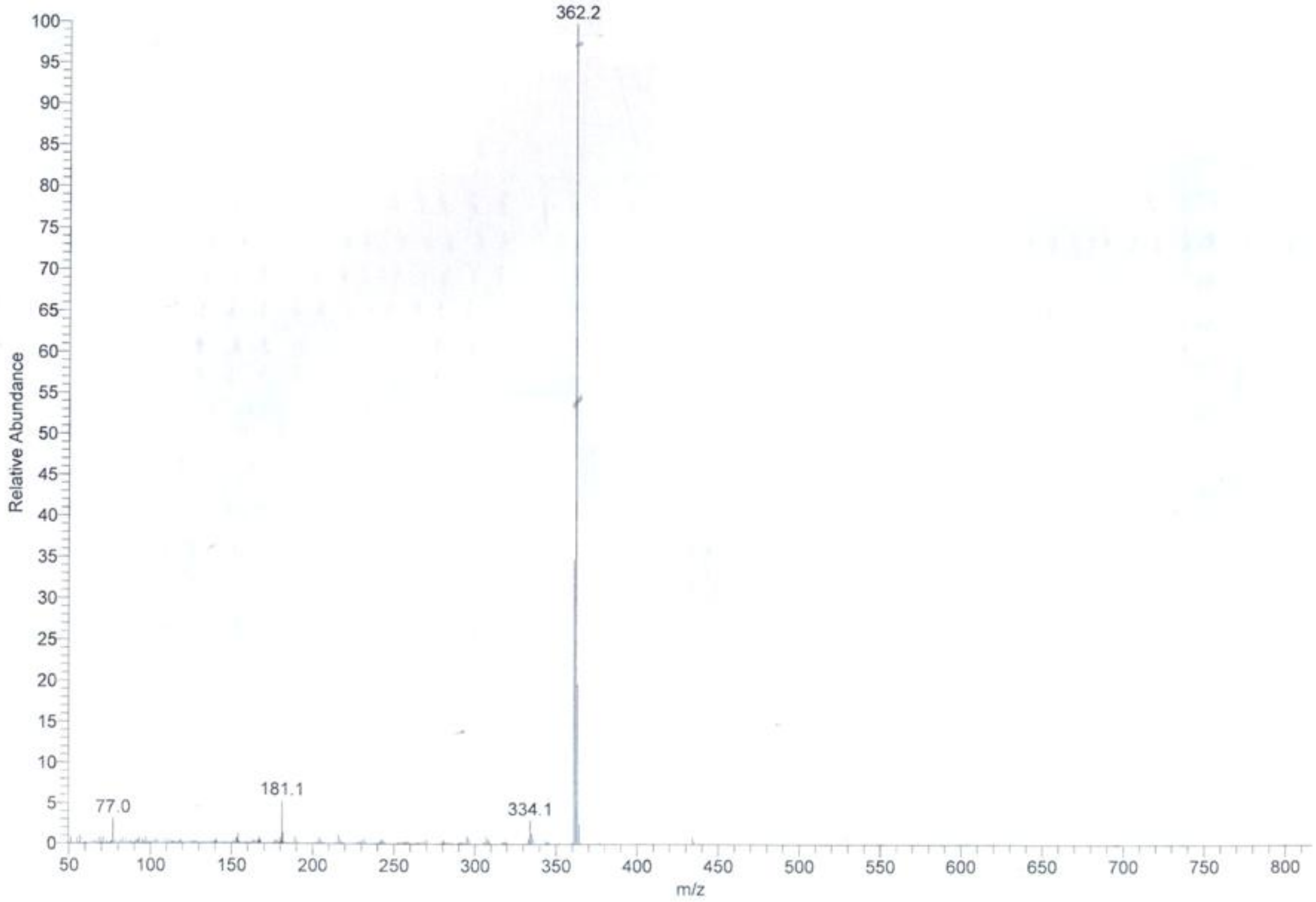

Compound 12a

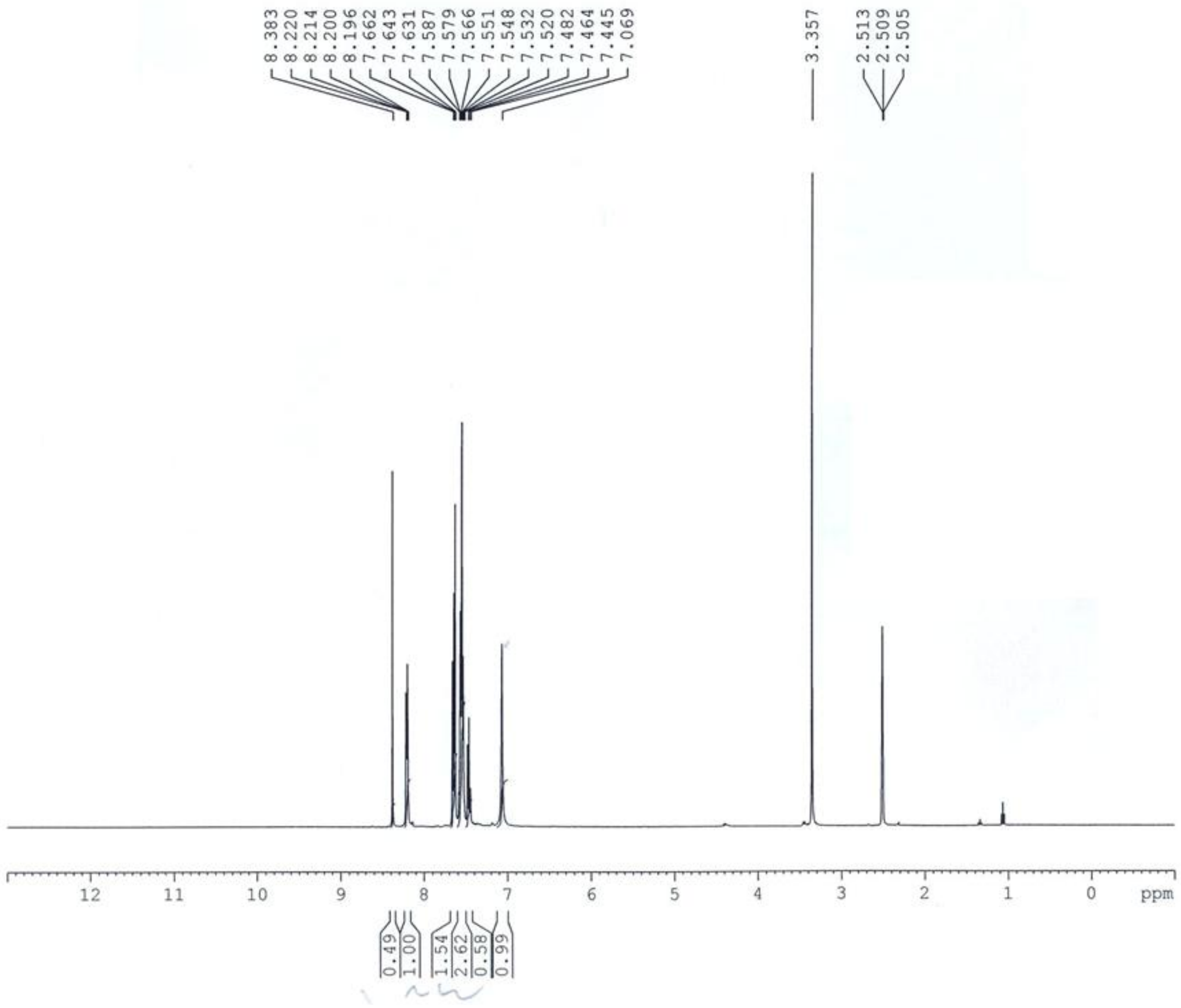

Compound 12a

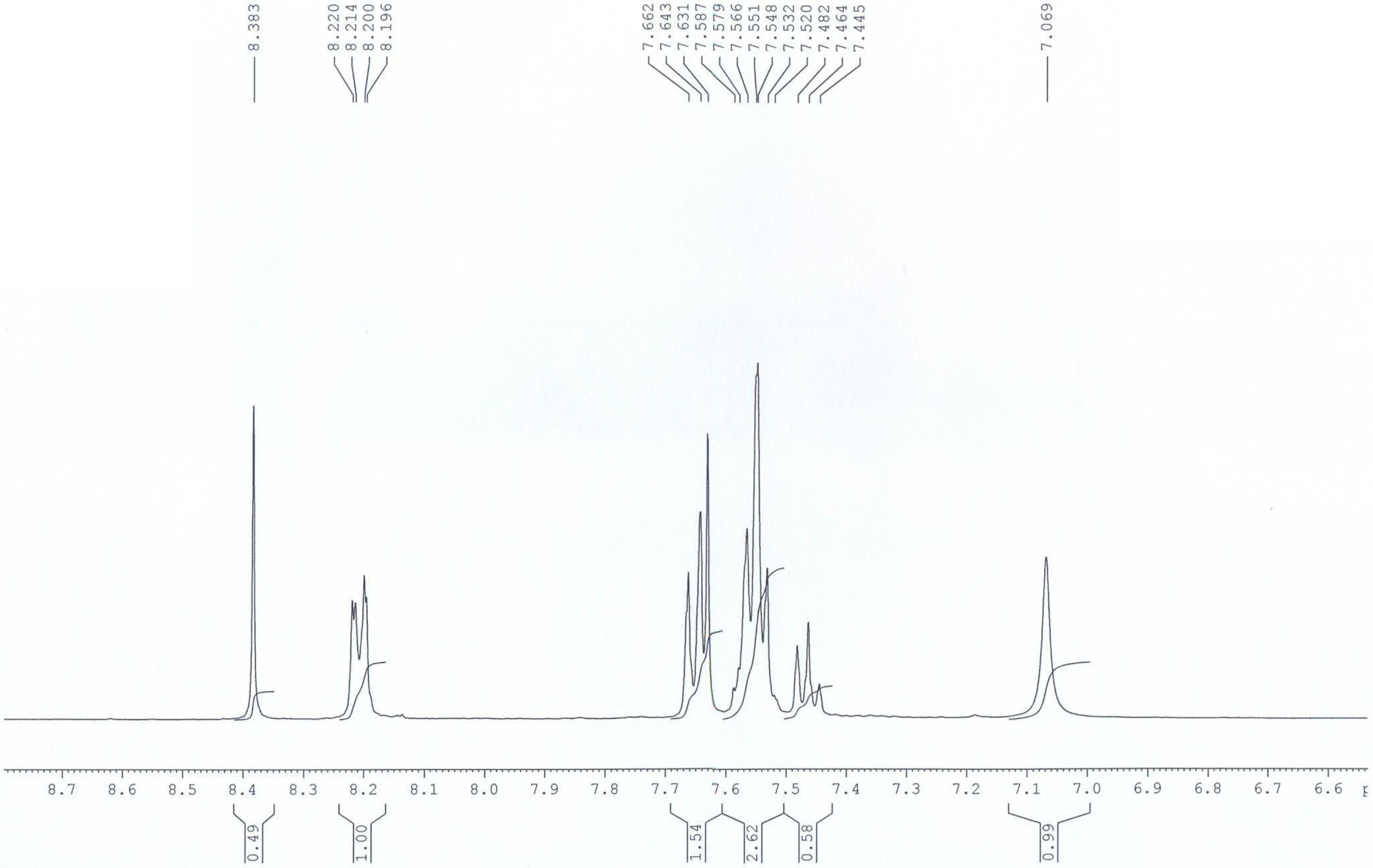

Compound 12a

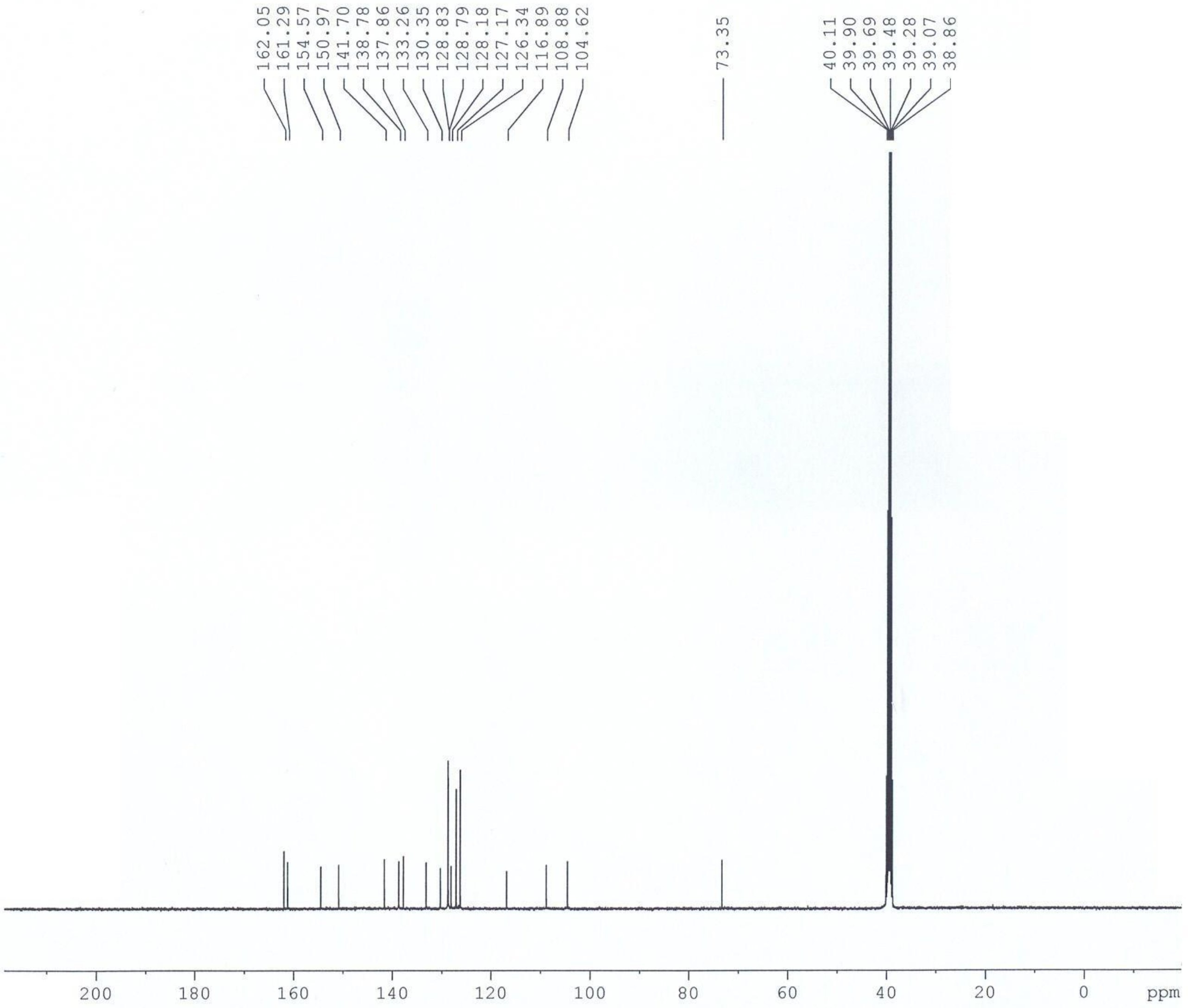

Compound 12a

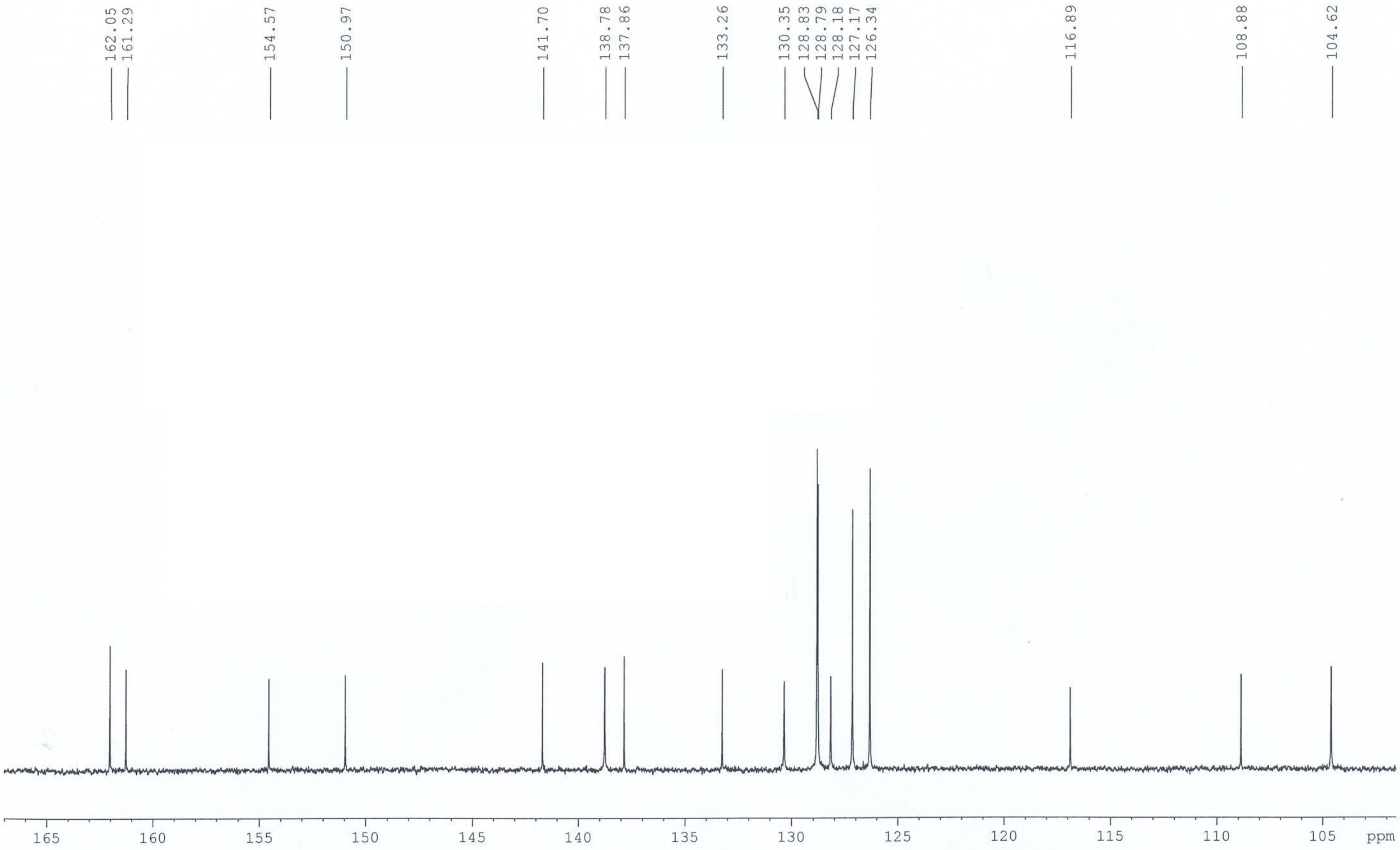

Compound 12a

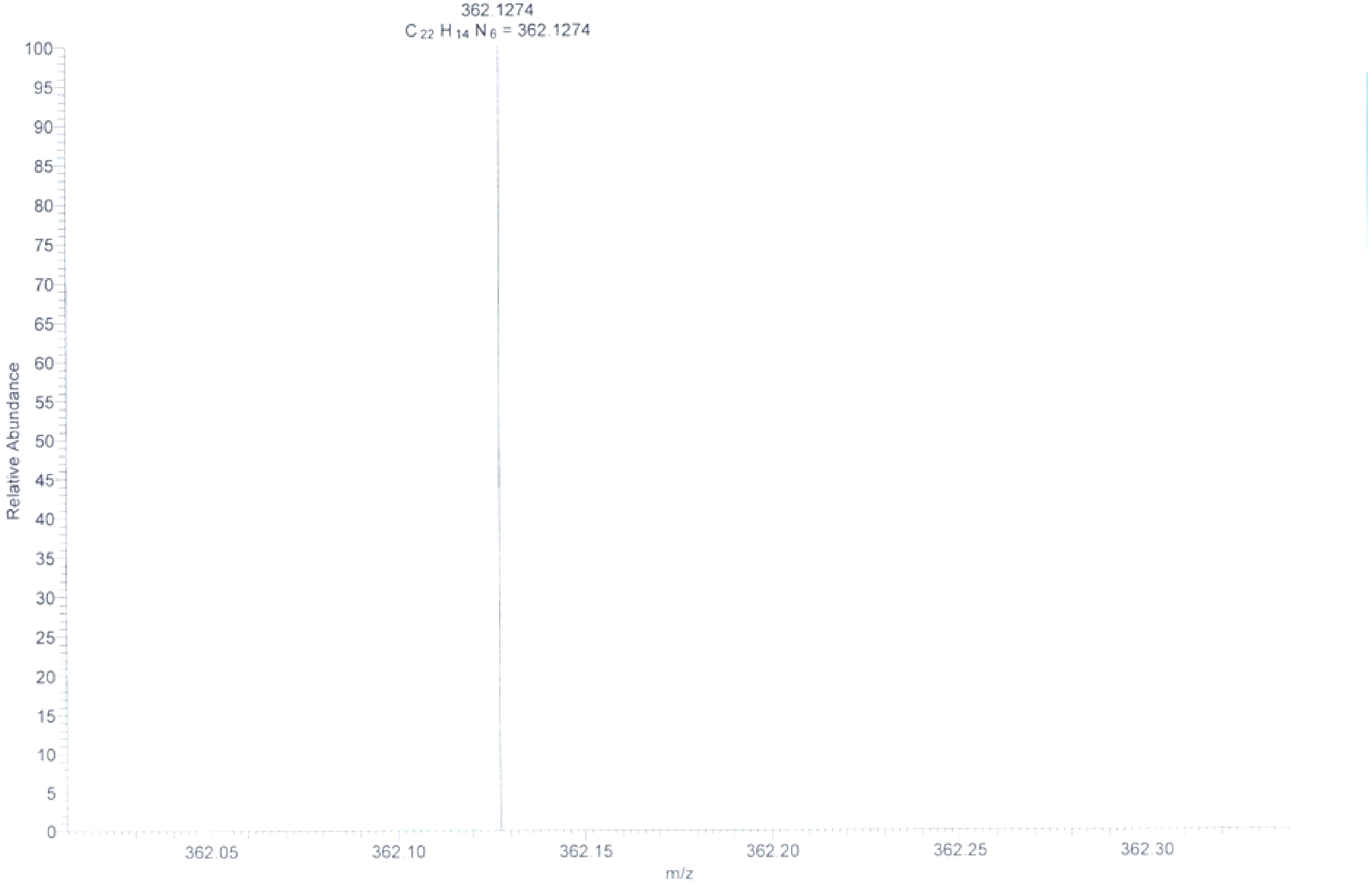

Compound 12b

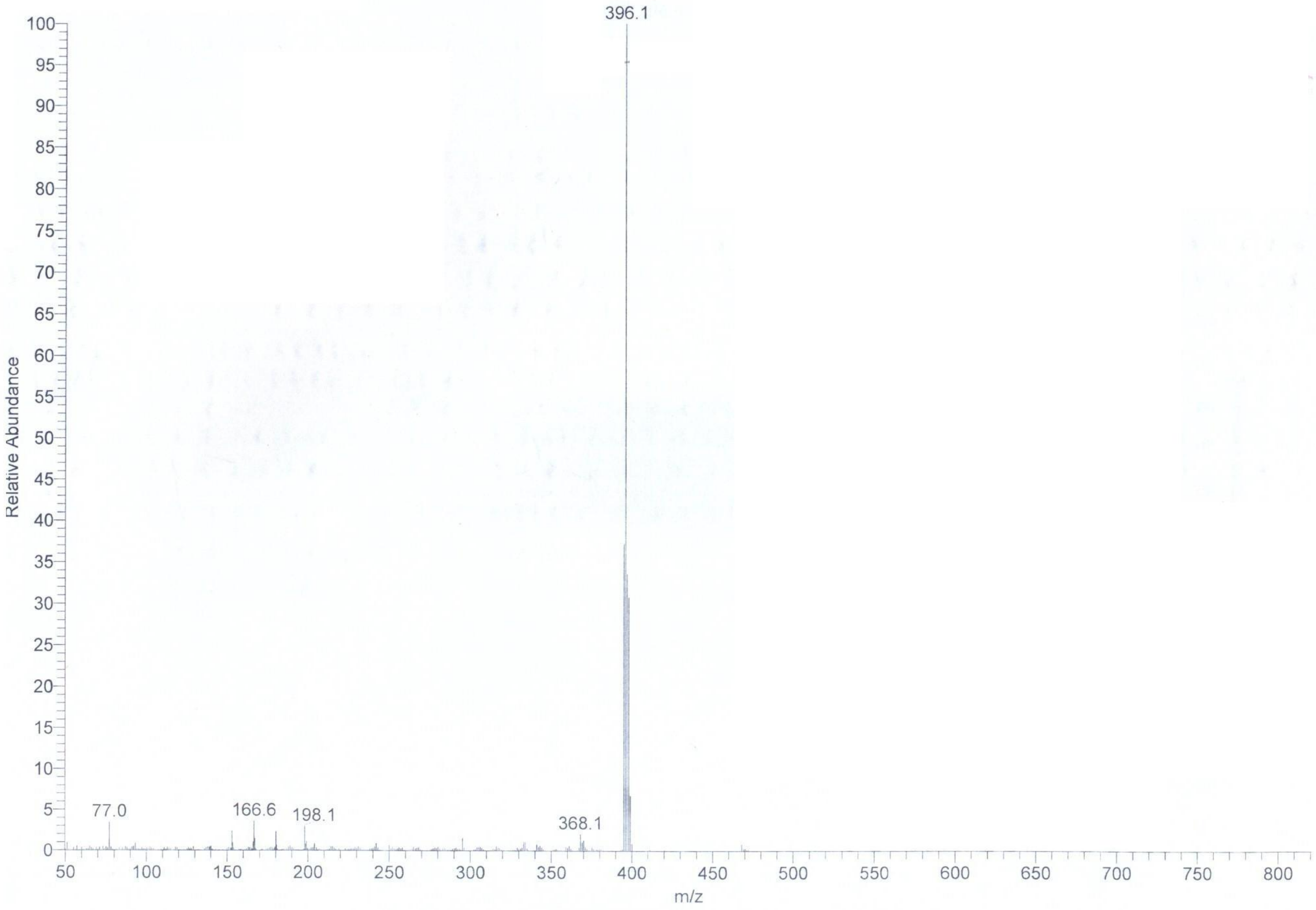

Compound 12b

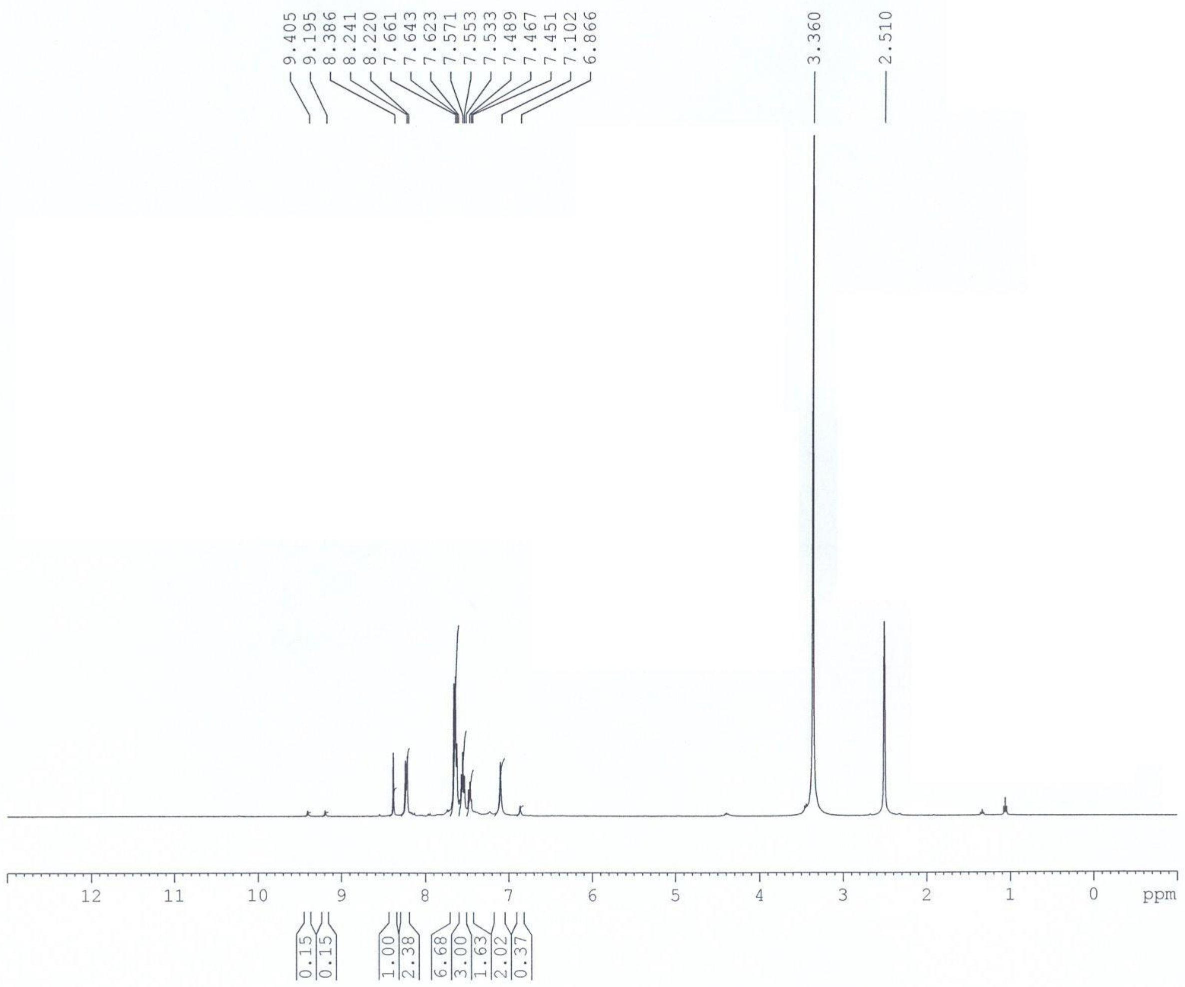

Compound 12b

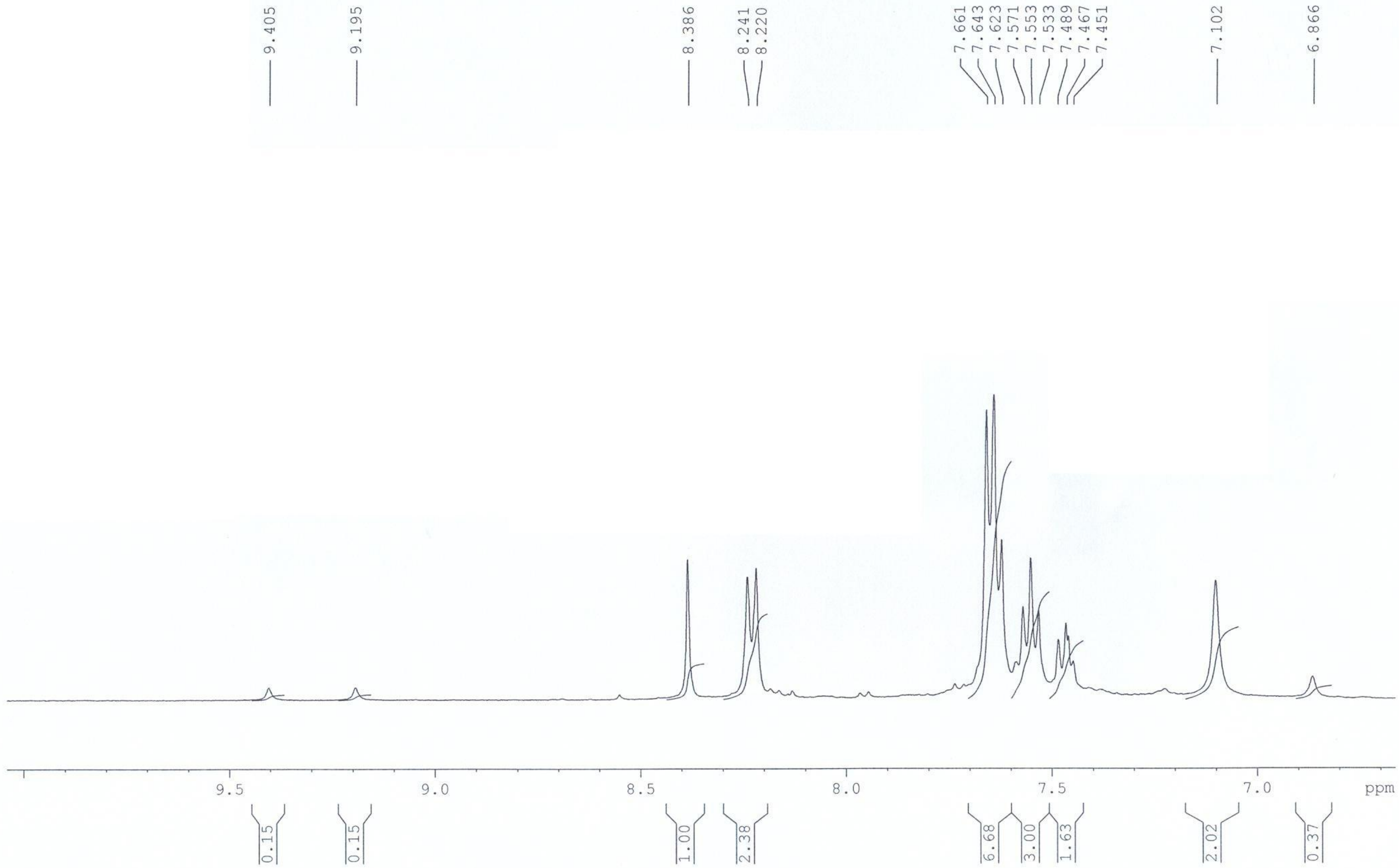

Compound 12b

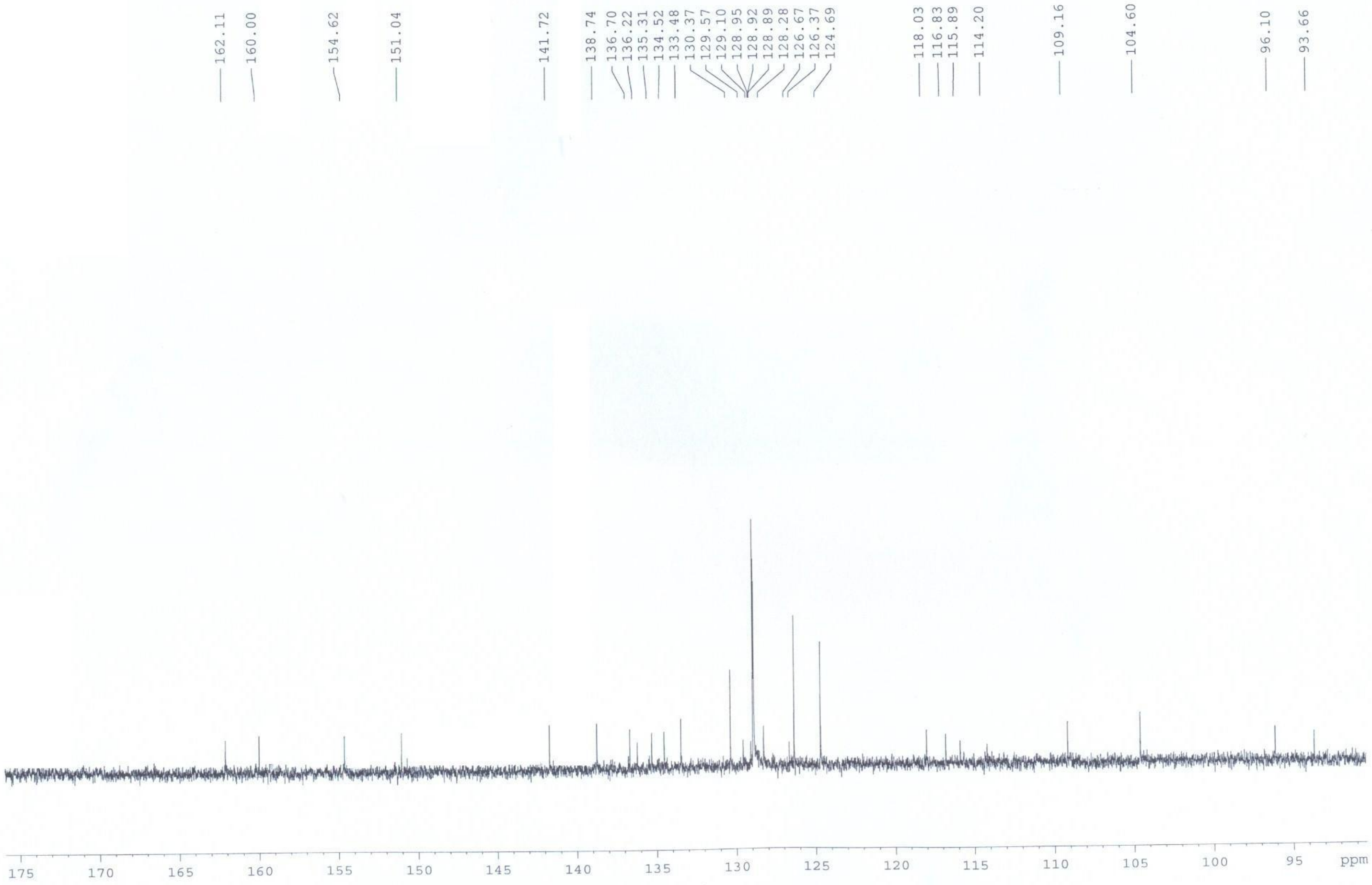

Compound 12b

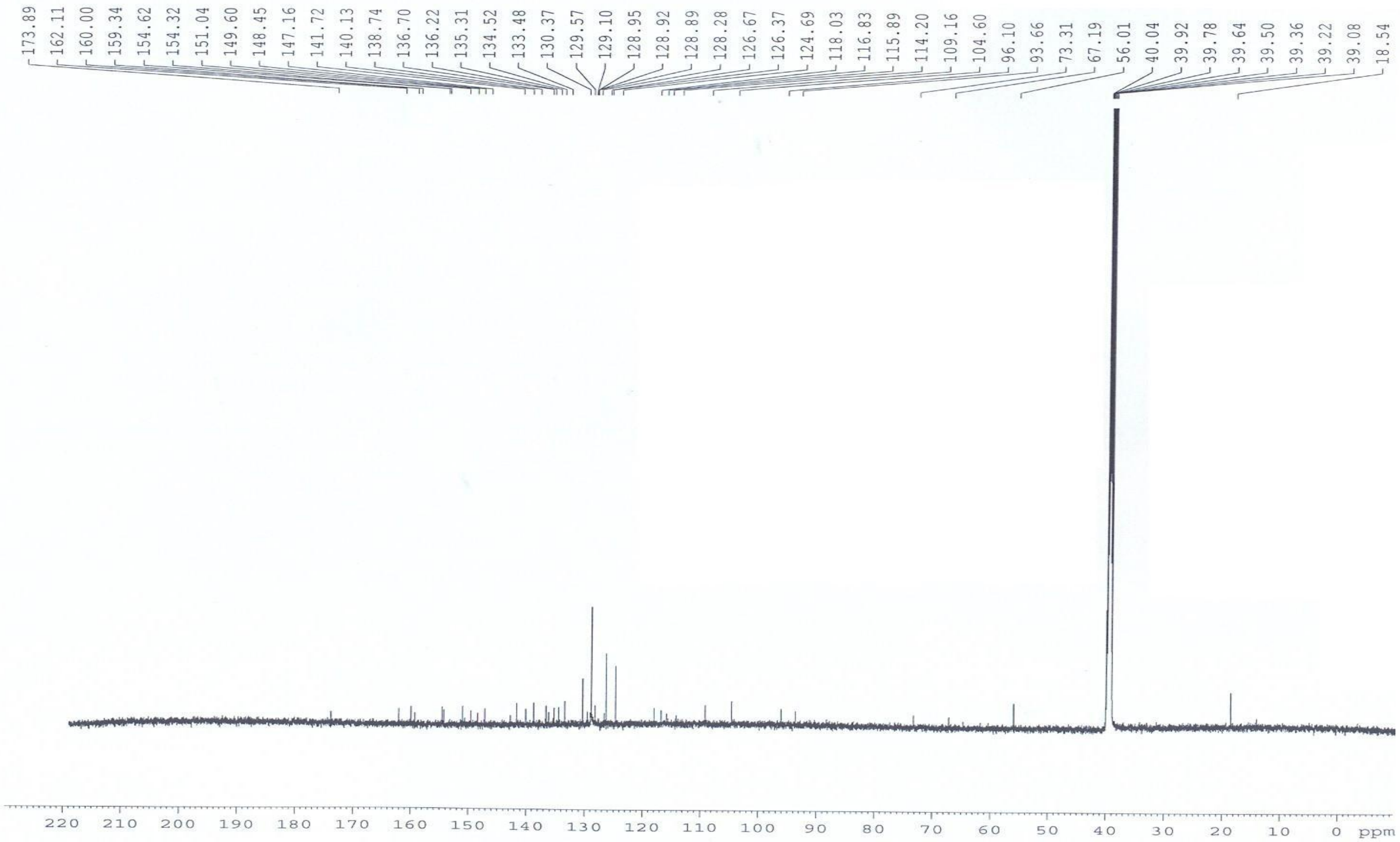

Compound 12b

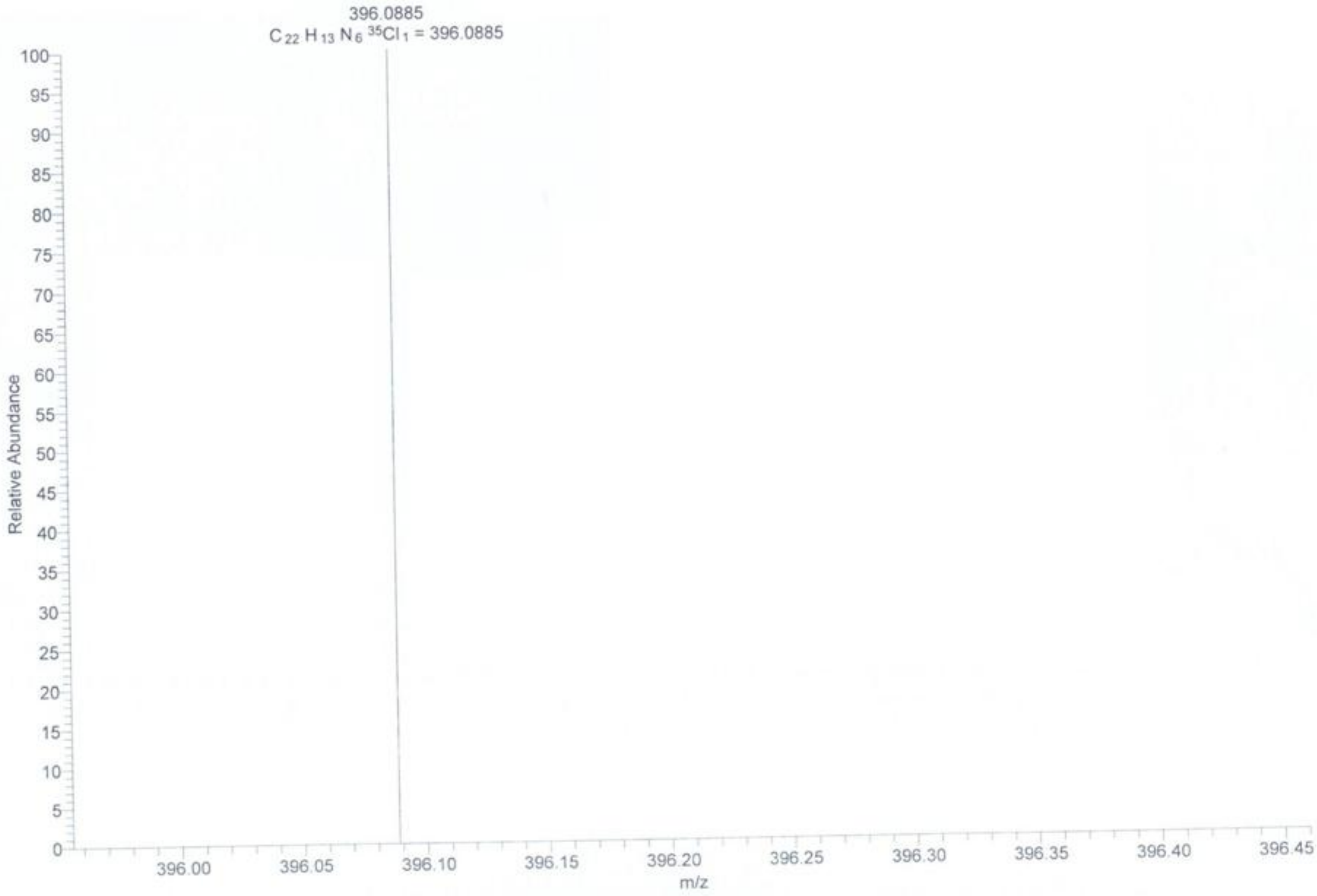

Compound 12b

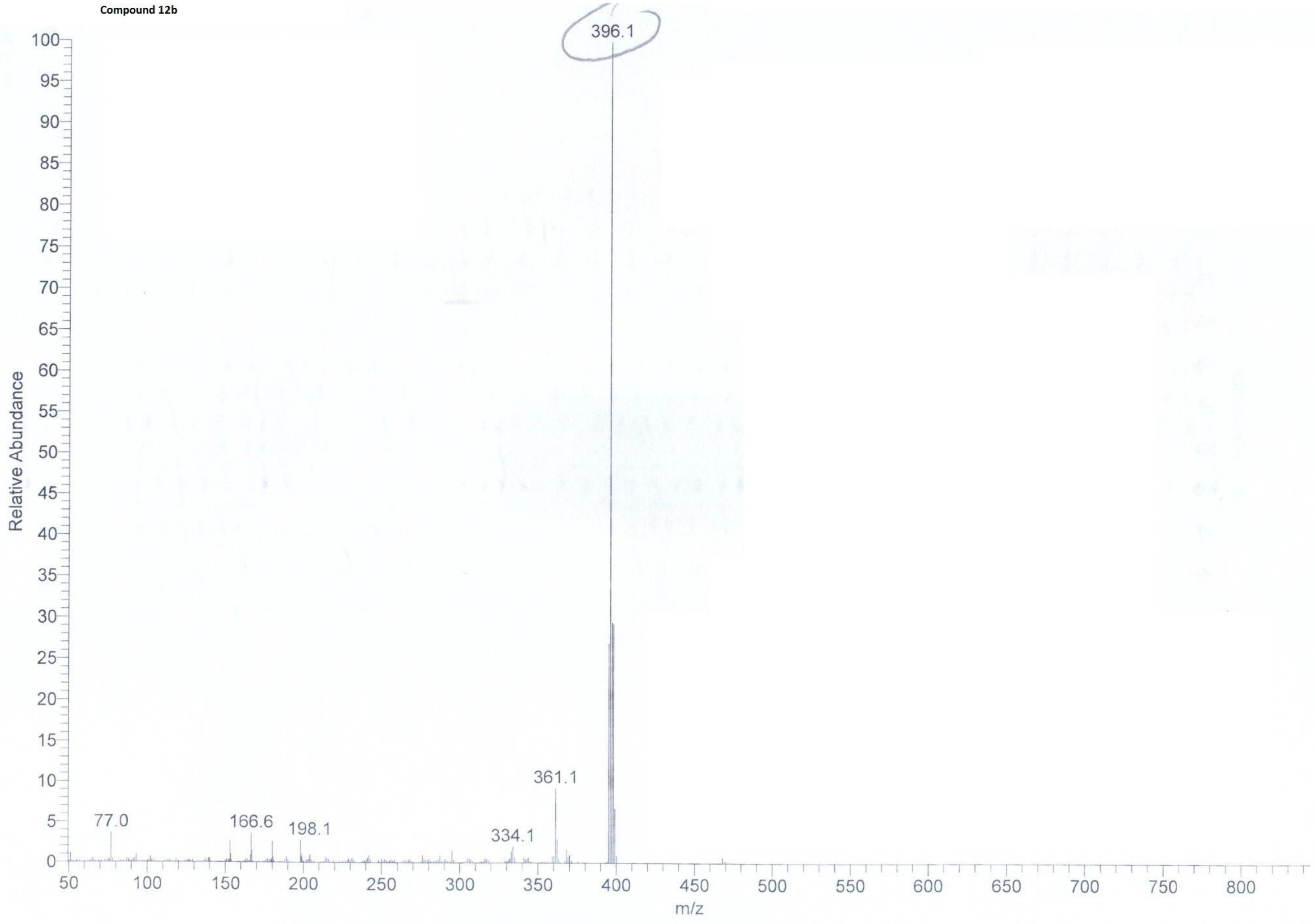

Compound 12b

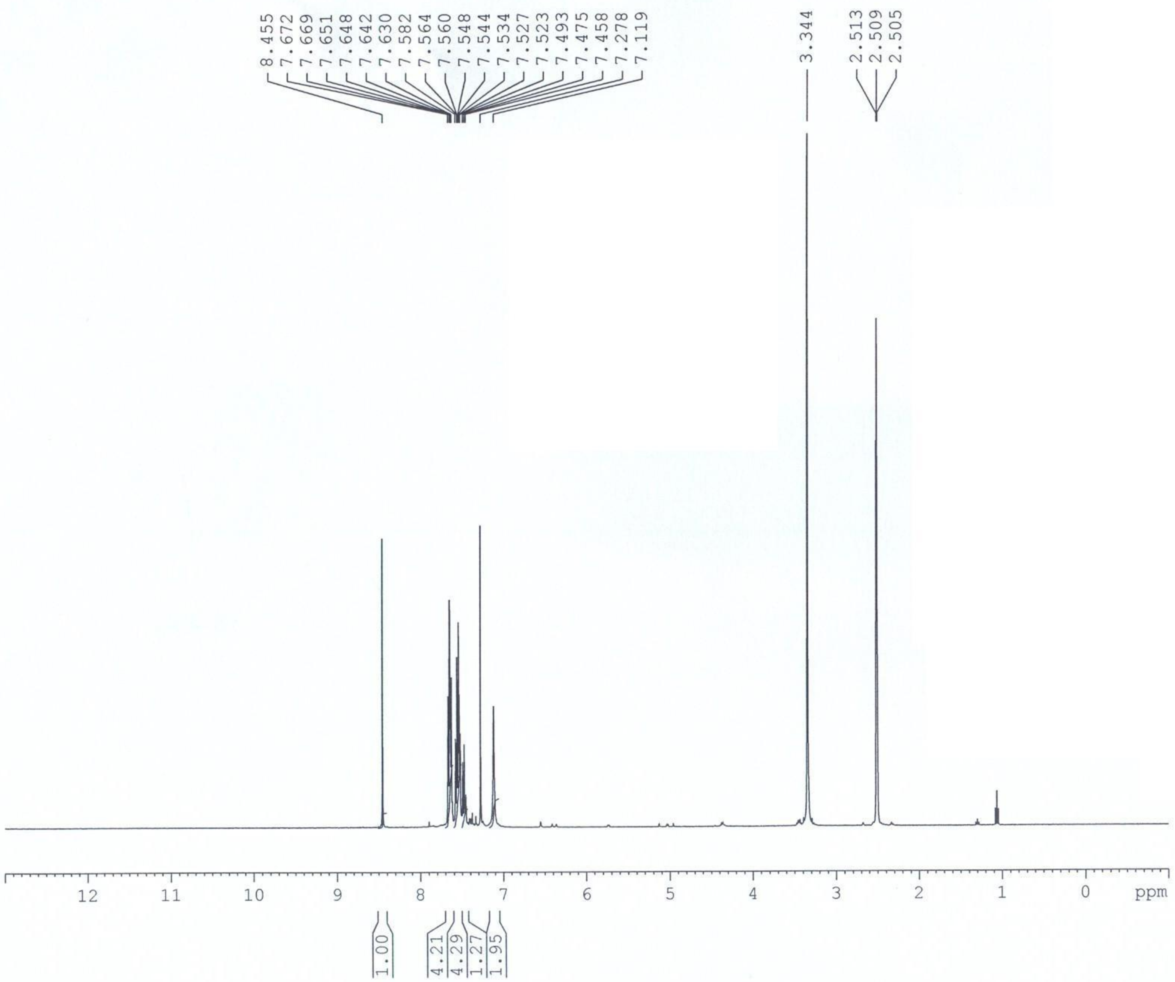

Compound 12b

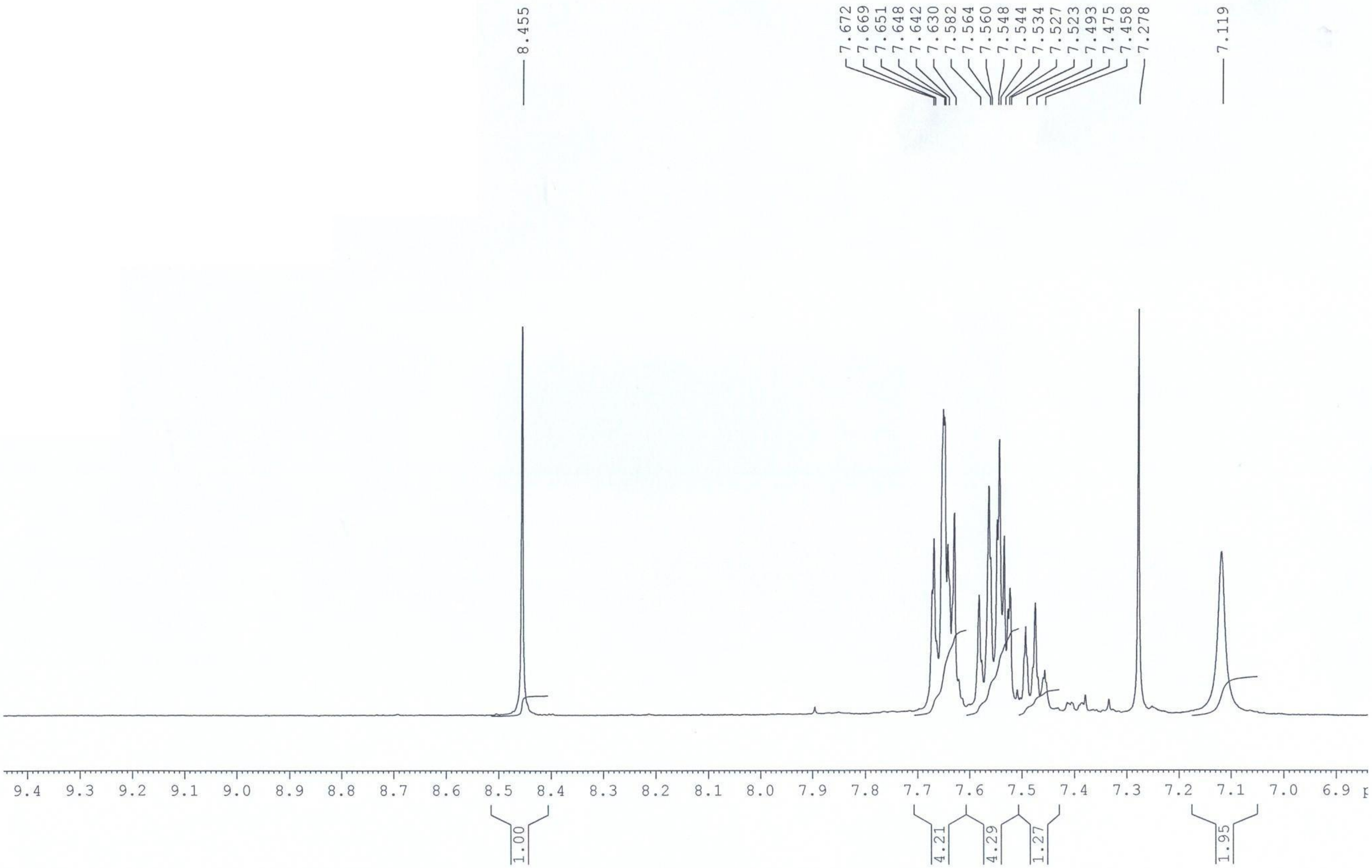

Compound 12b

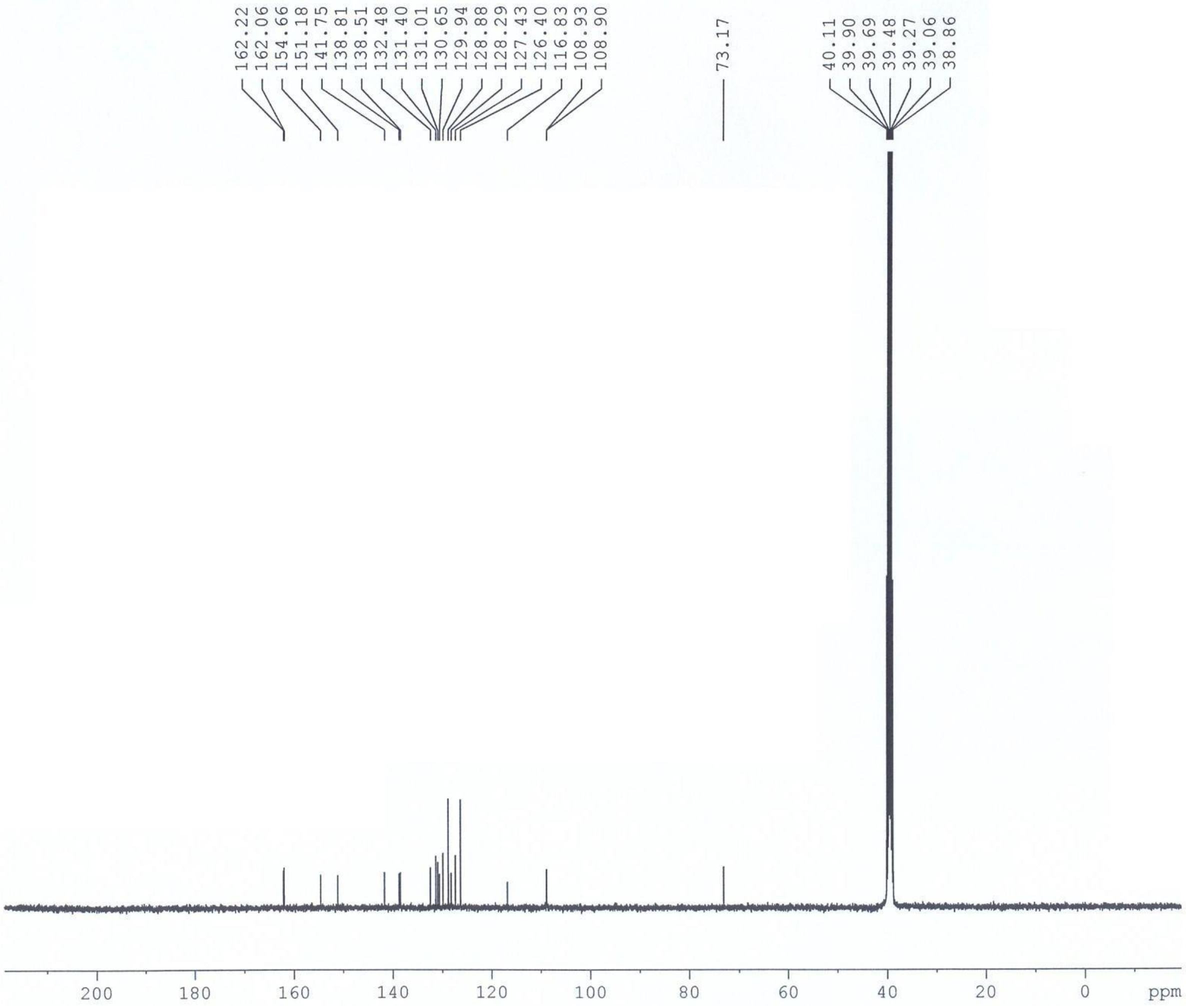

Compound 12b

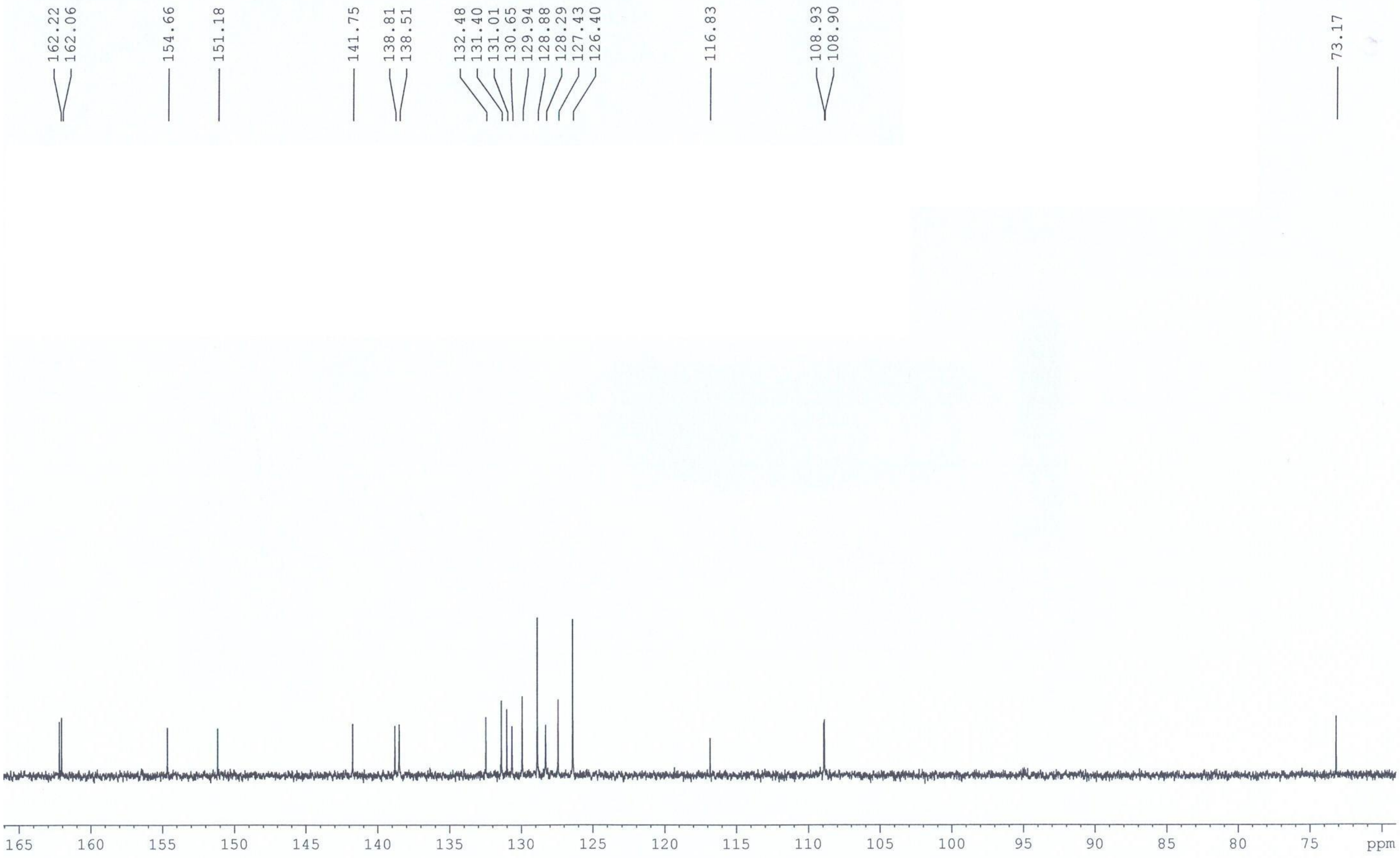

Compound 12c

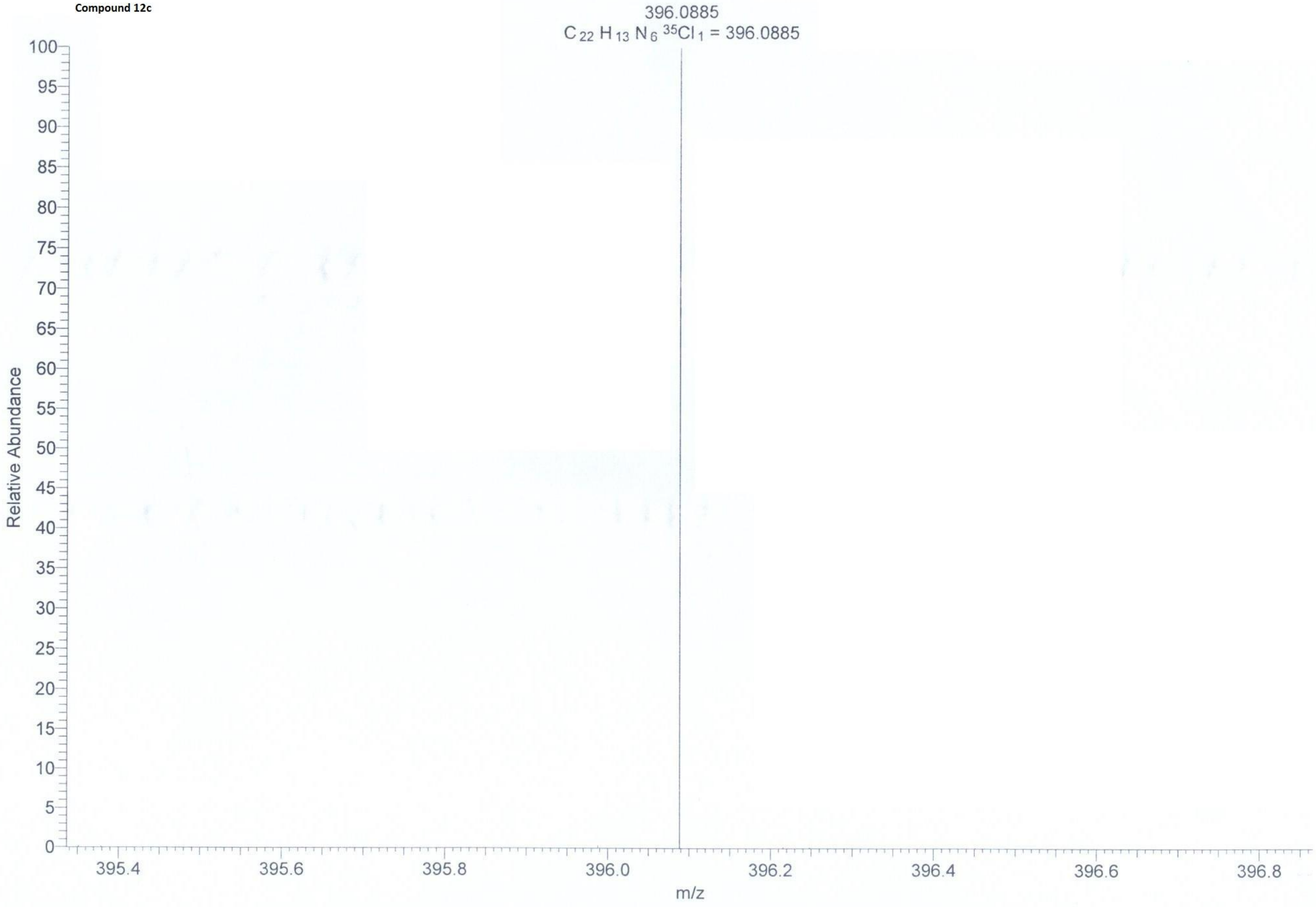

Compound 12d

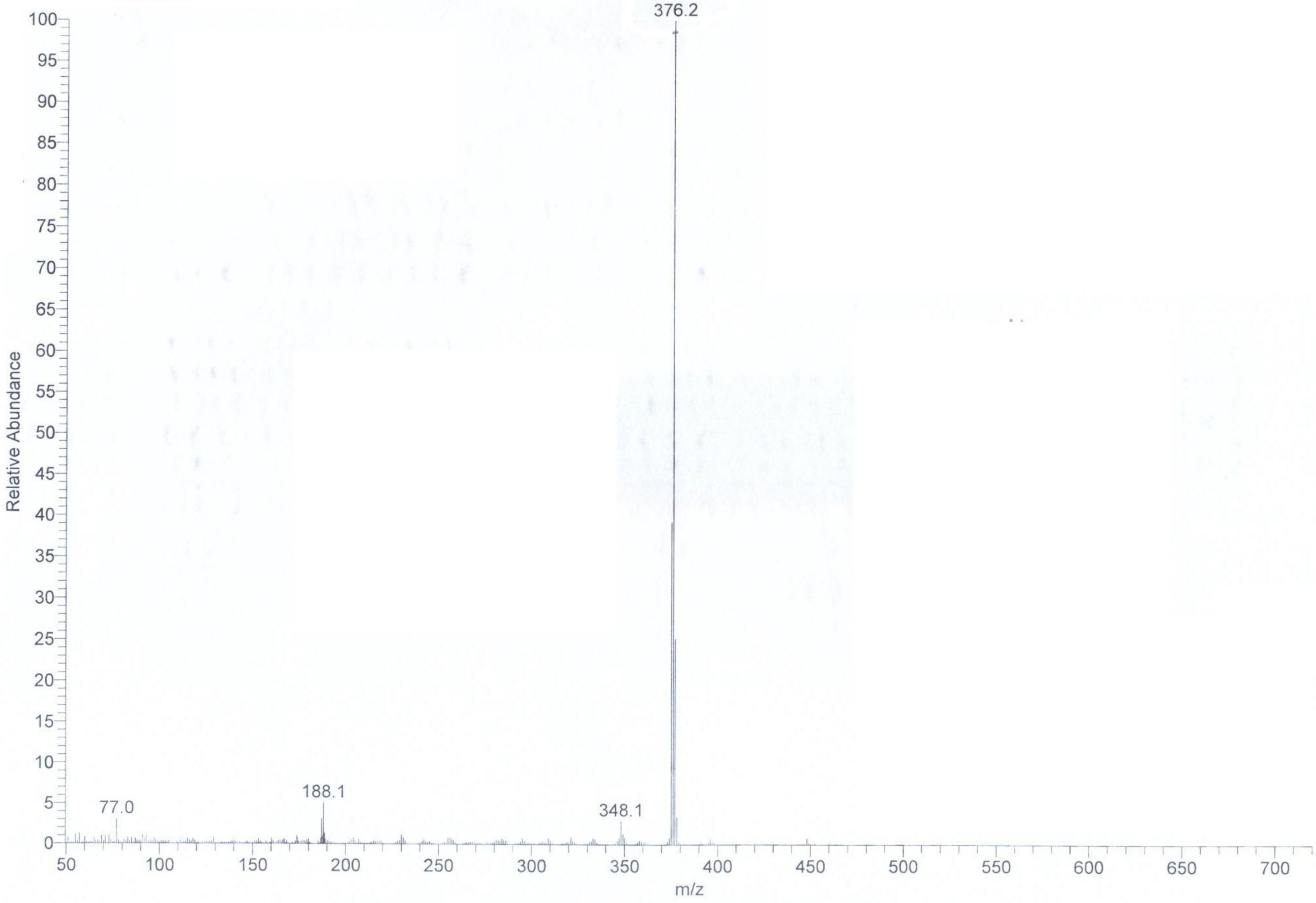

Compound 12d

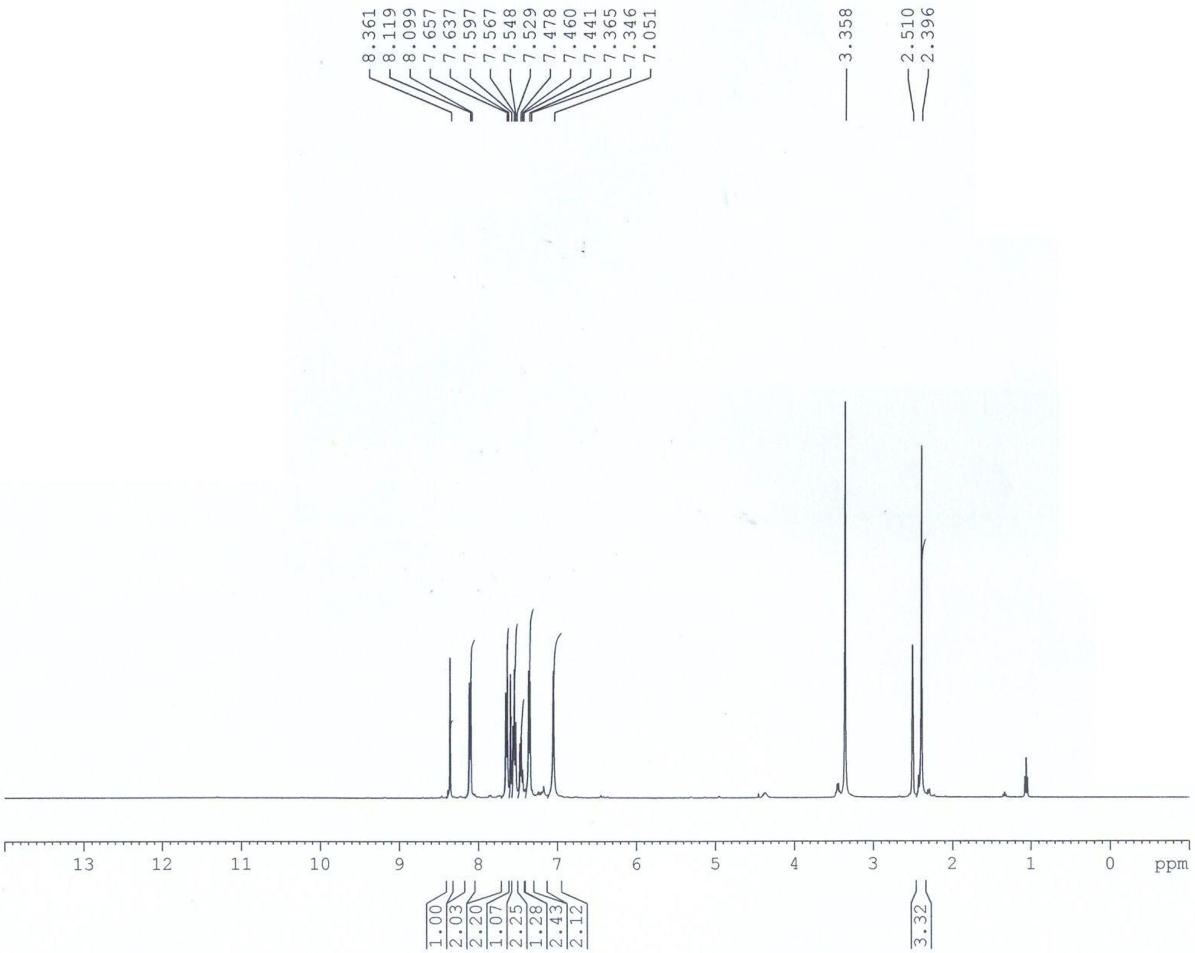

Compound 12d

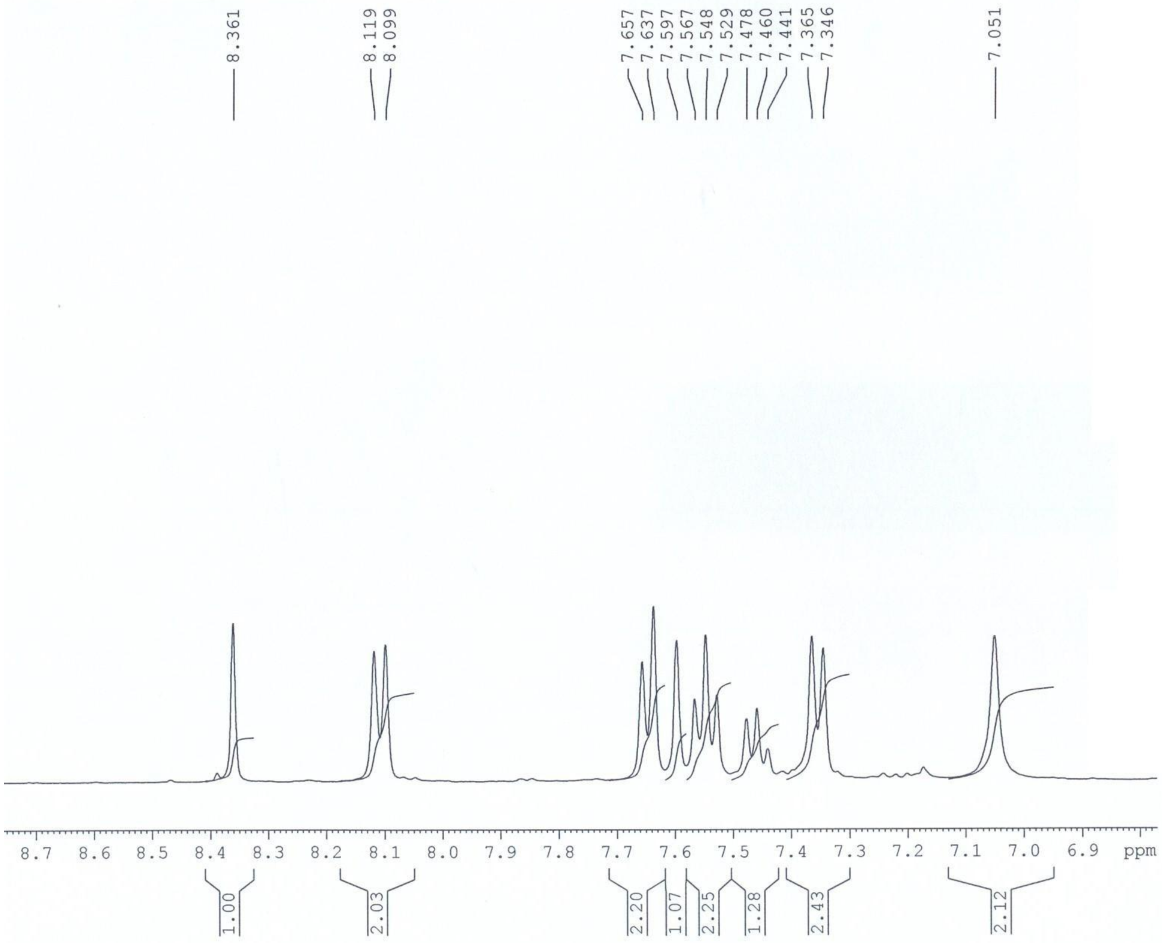

Compound 12d

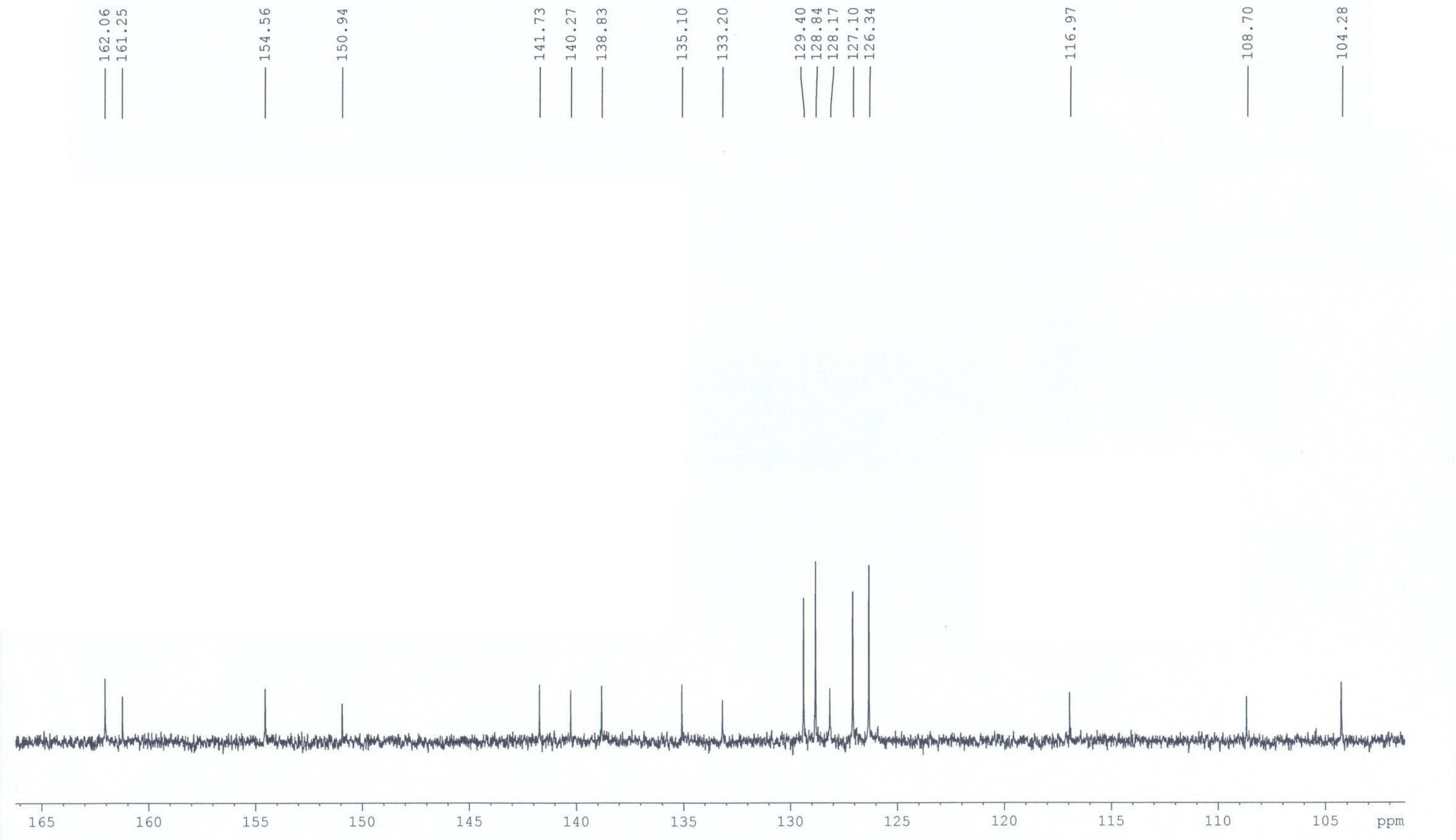

Compound 12d

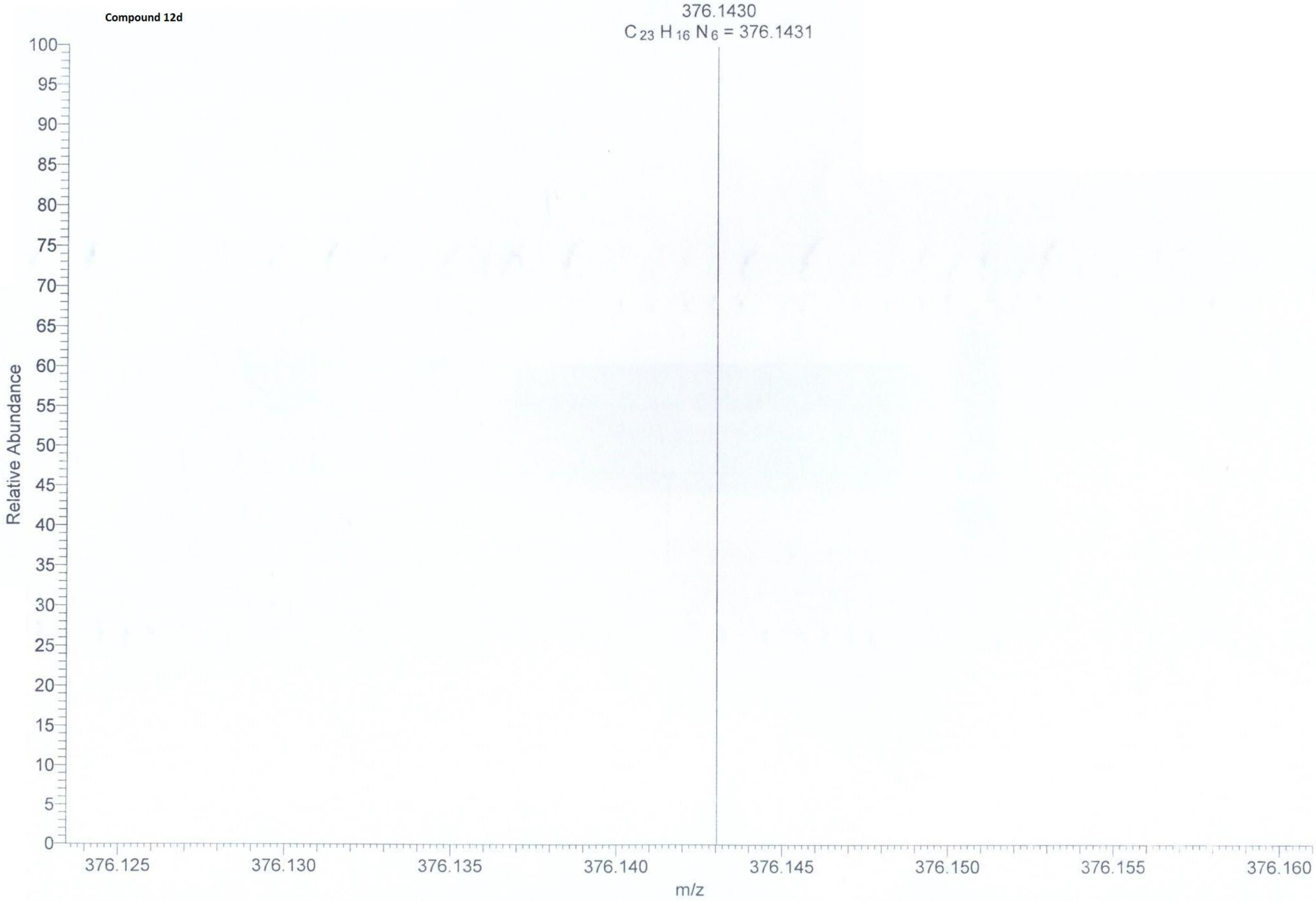

Compound 12d

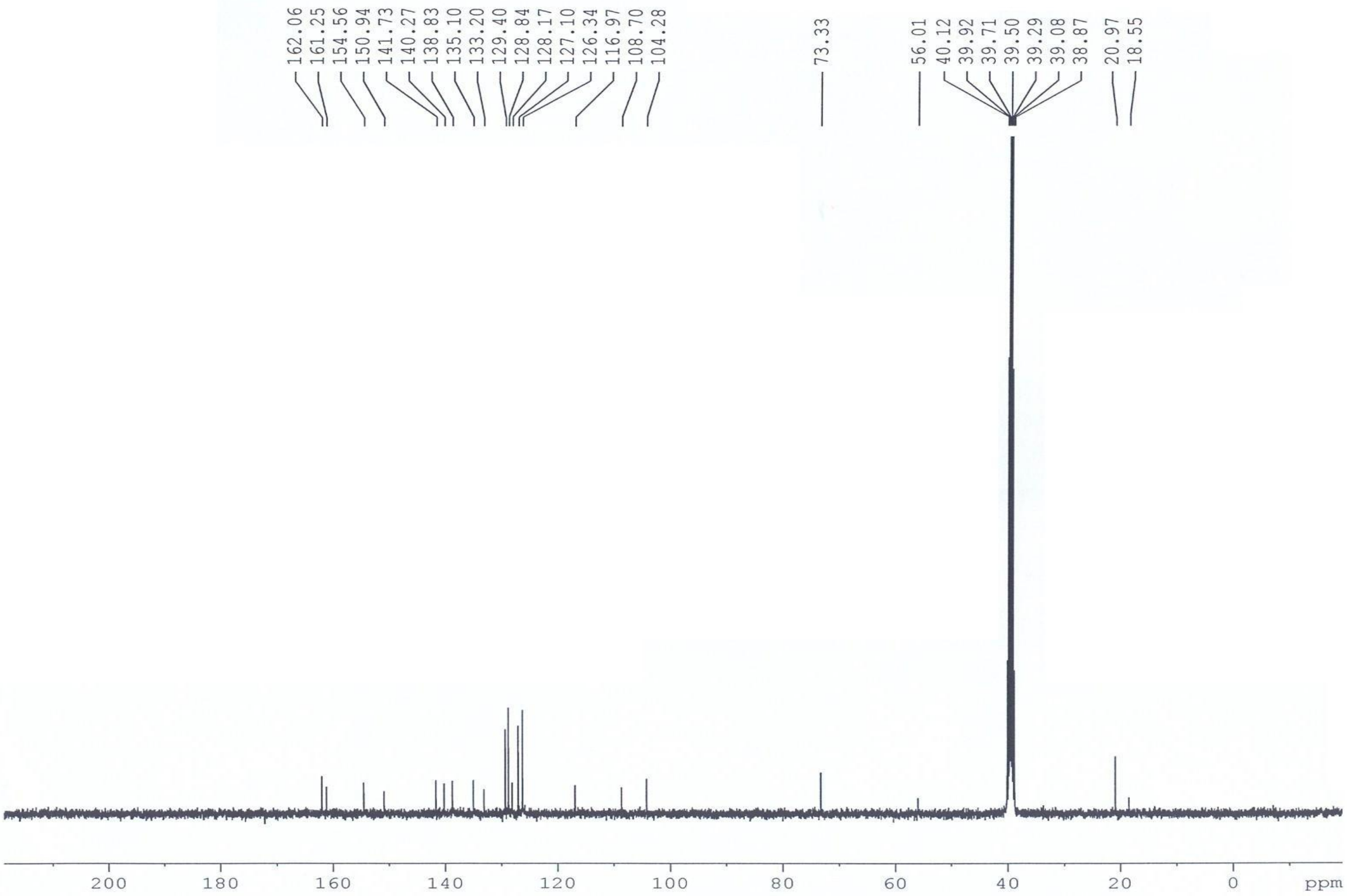

Compound 12e

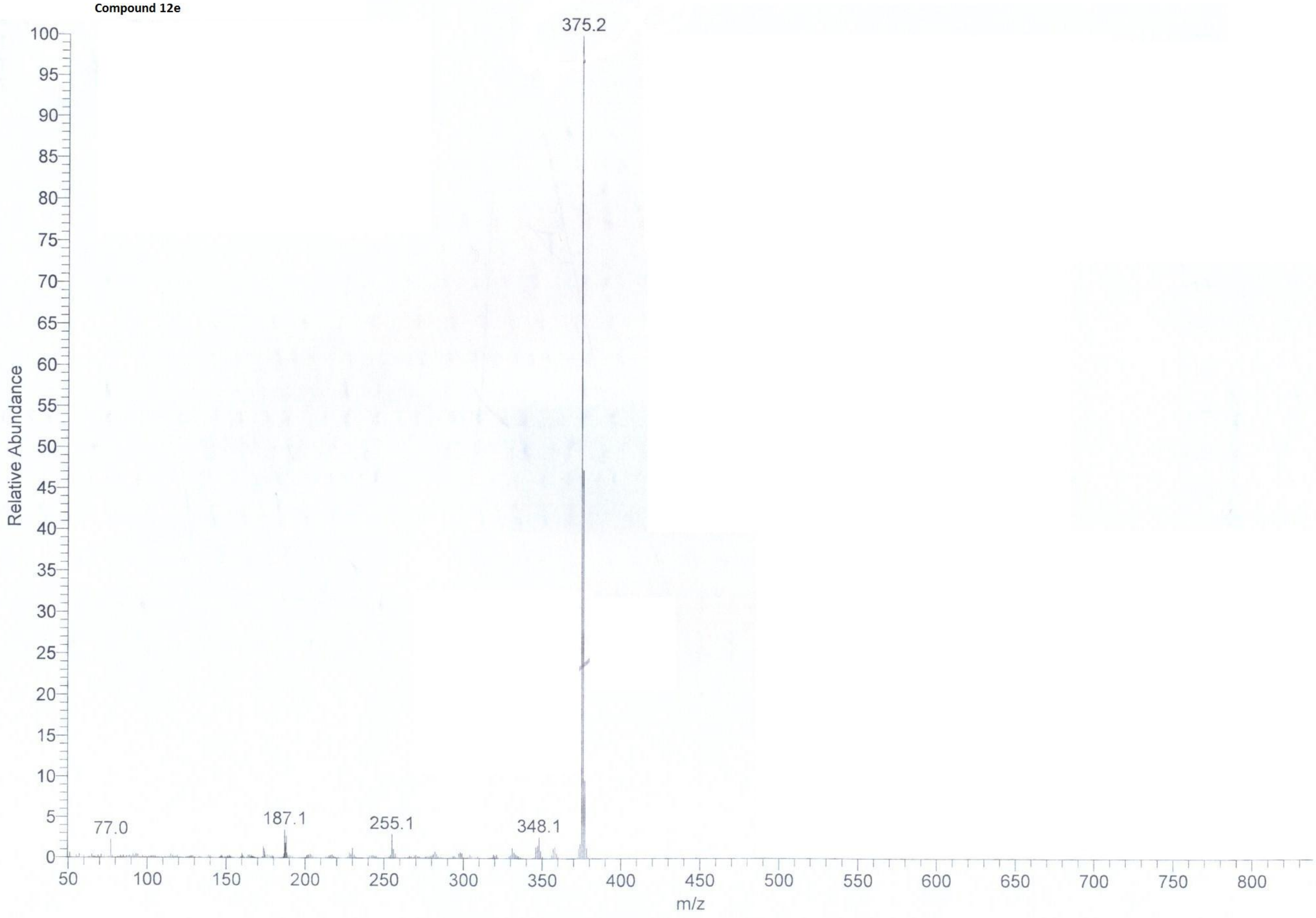

Compound 12e

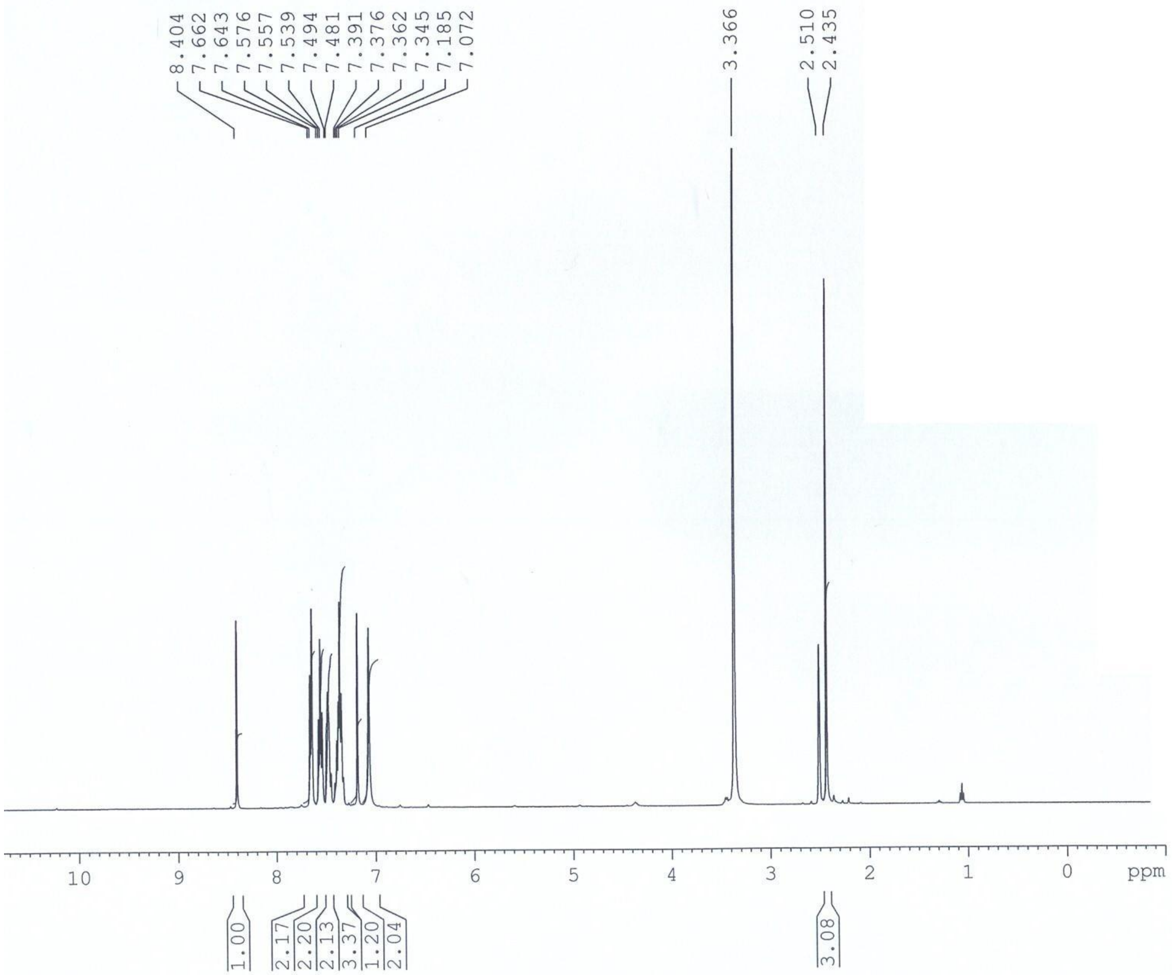

Compound 12e

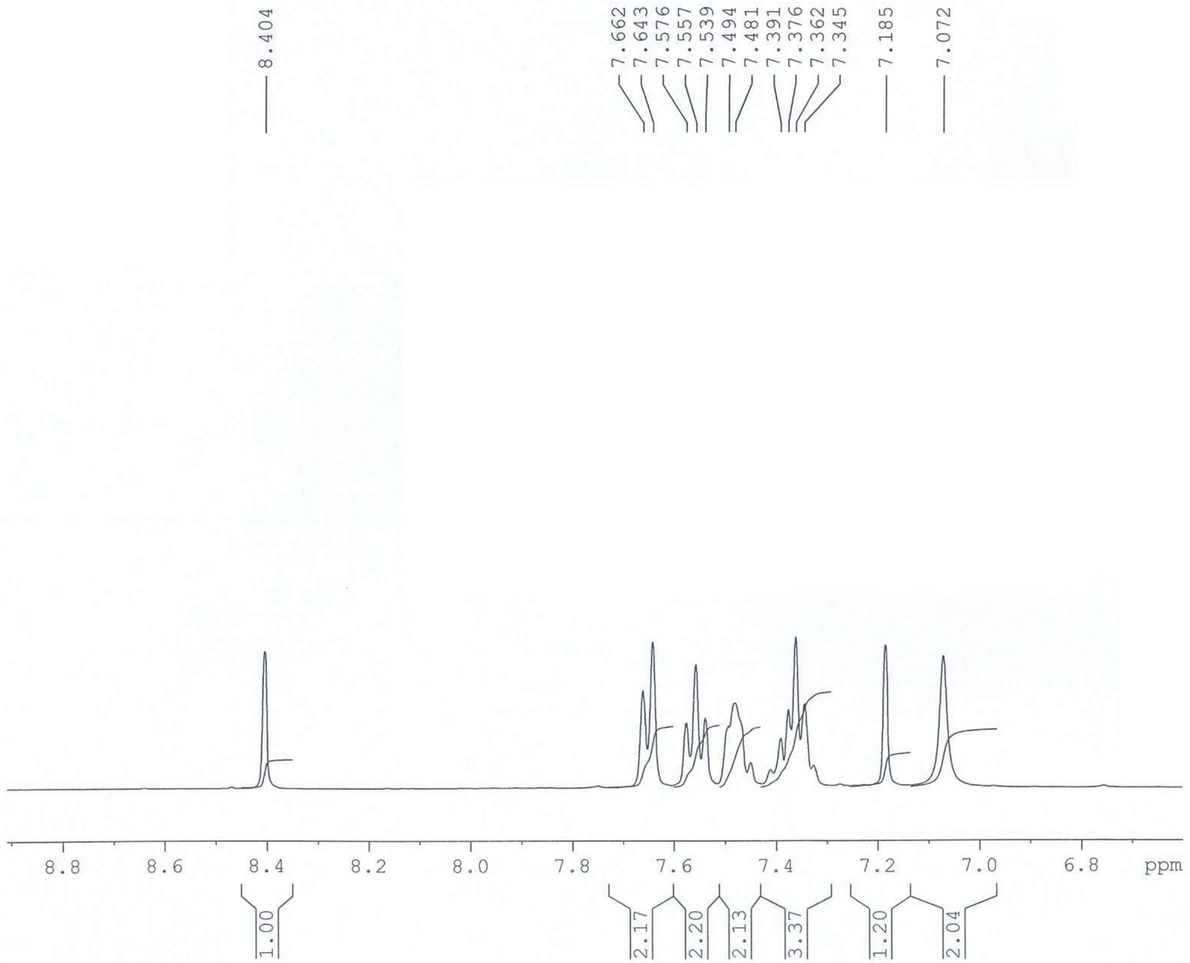

Compound 12e

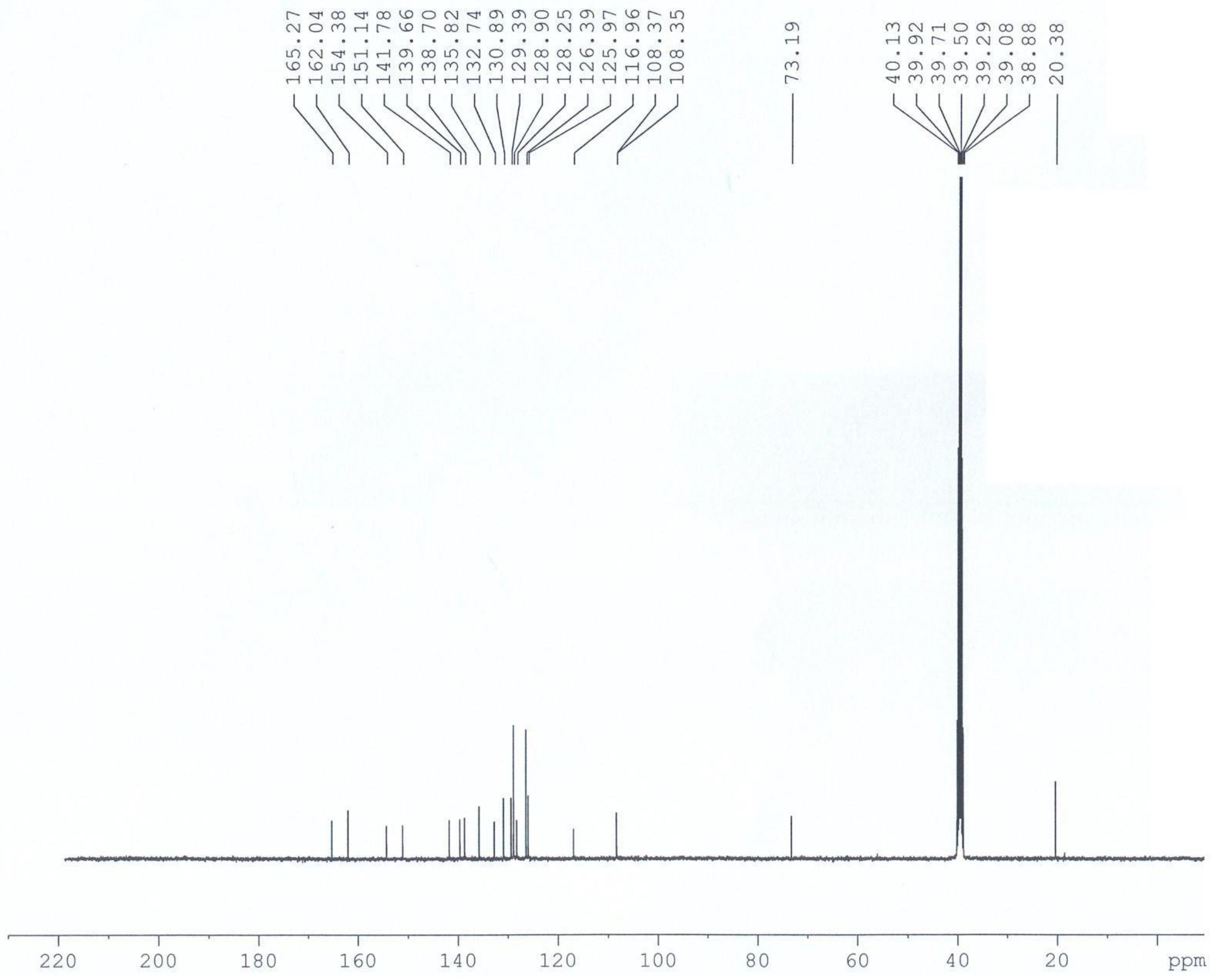

Compound 12e

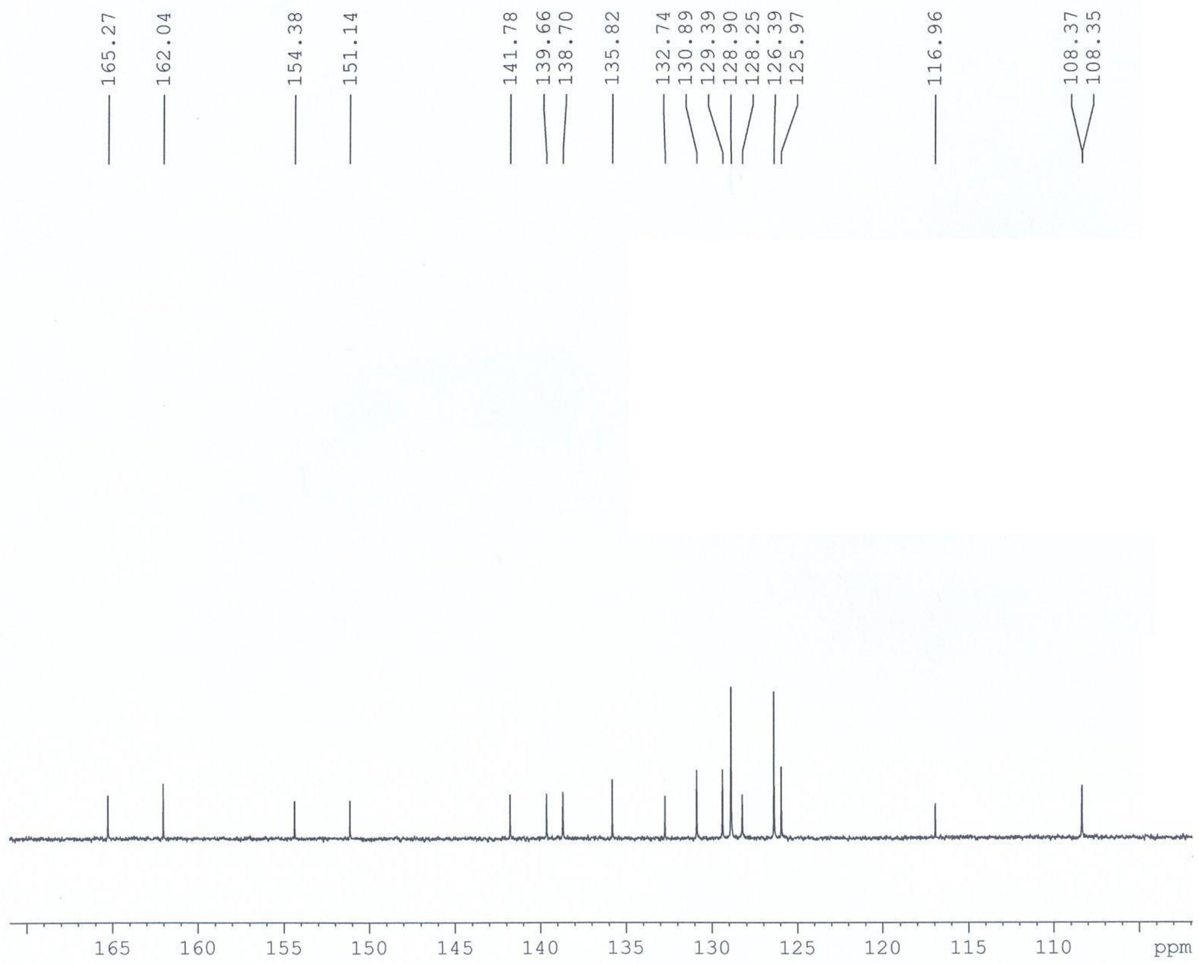

Compound 12e

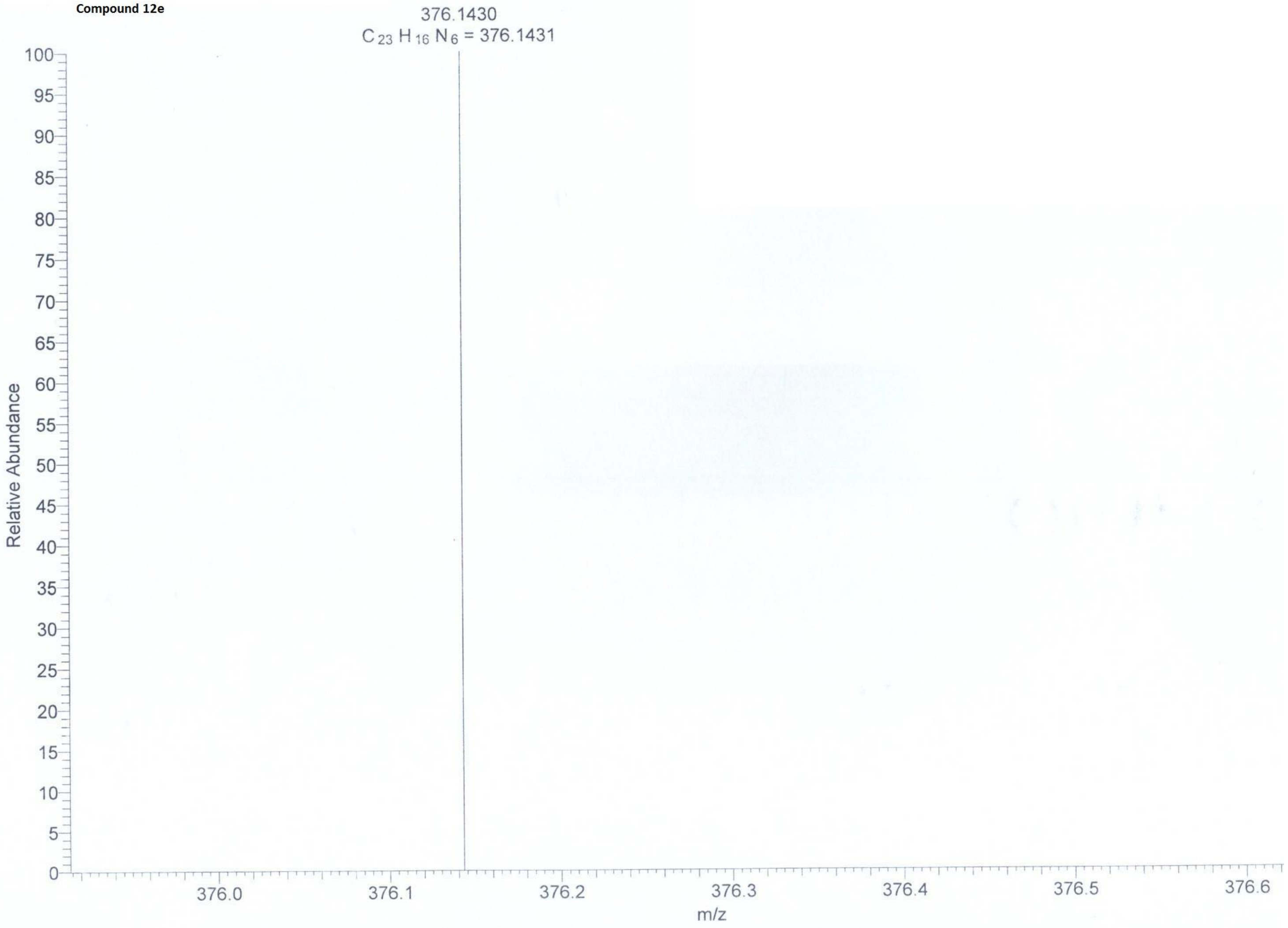

Compound 12f

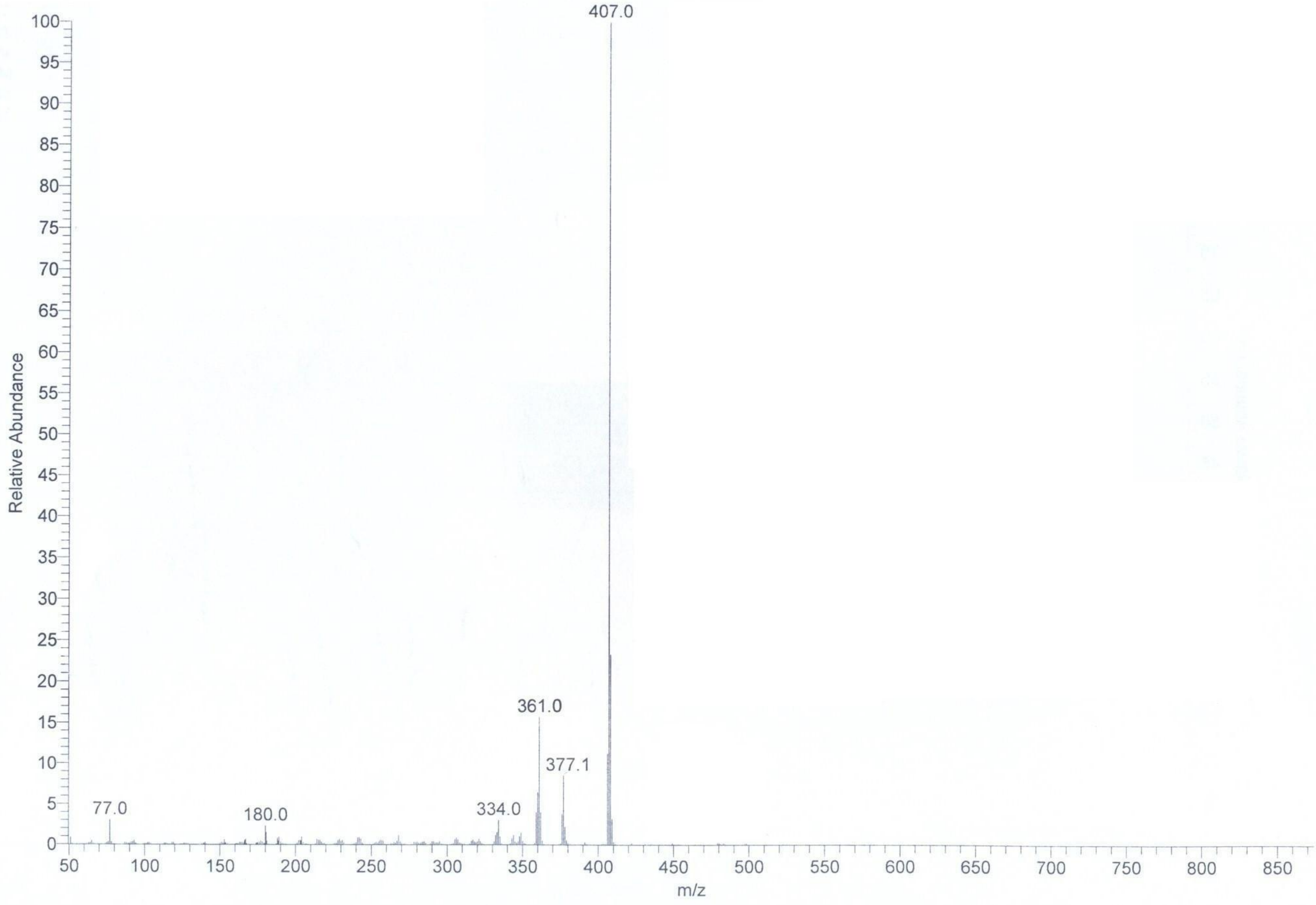

Compound 12f

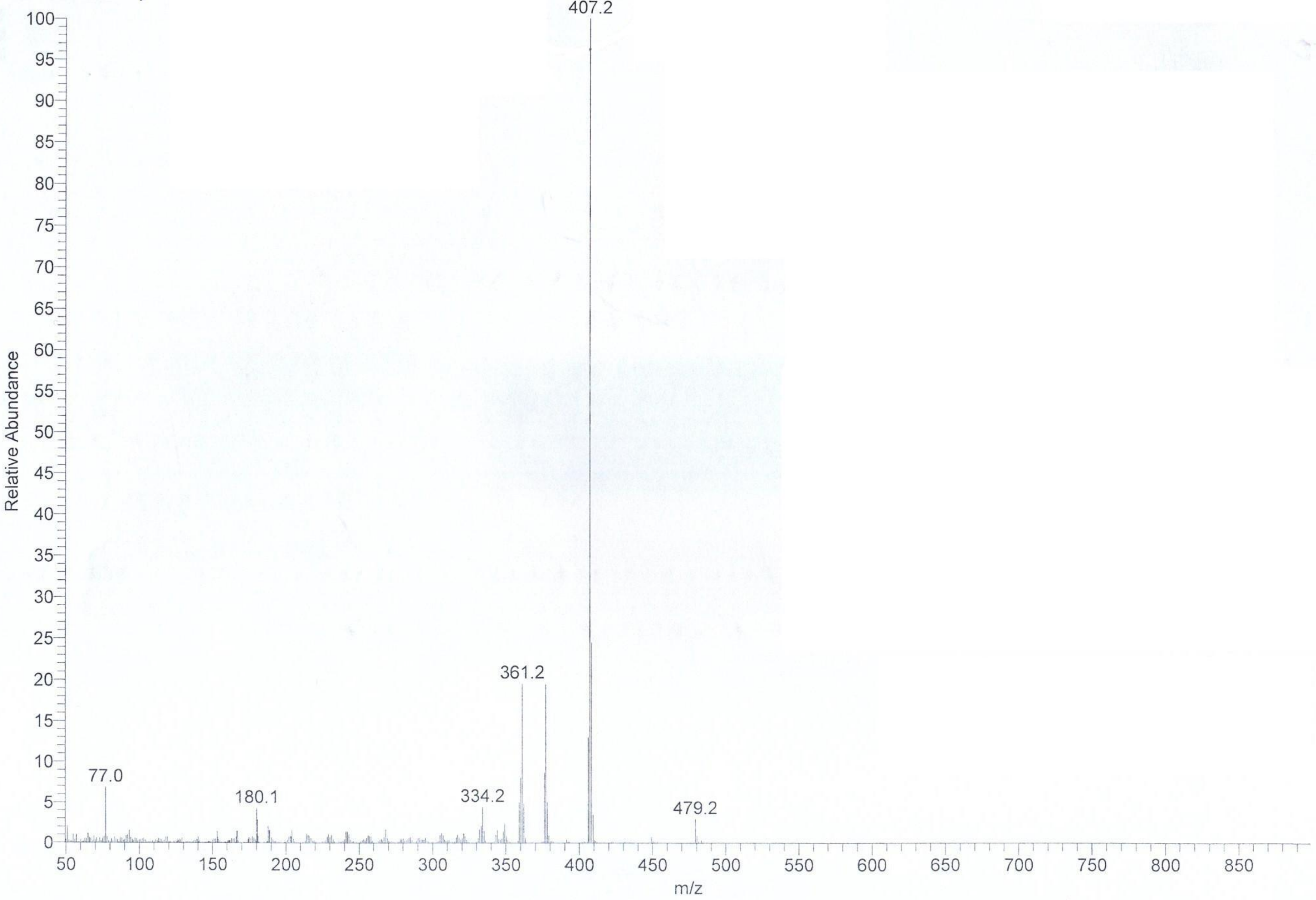

Compound 12f

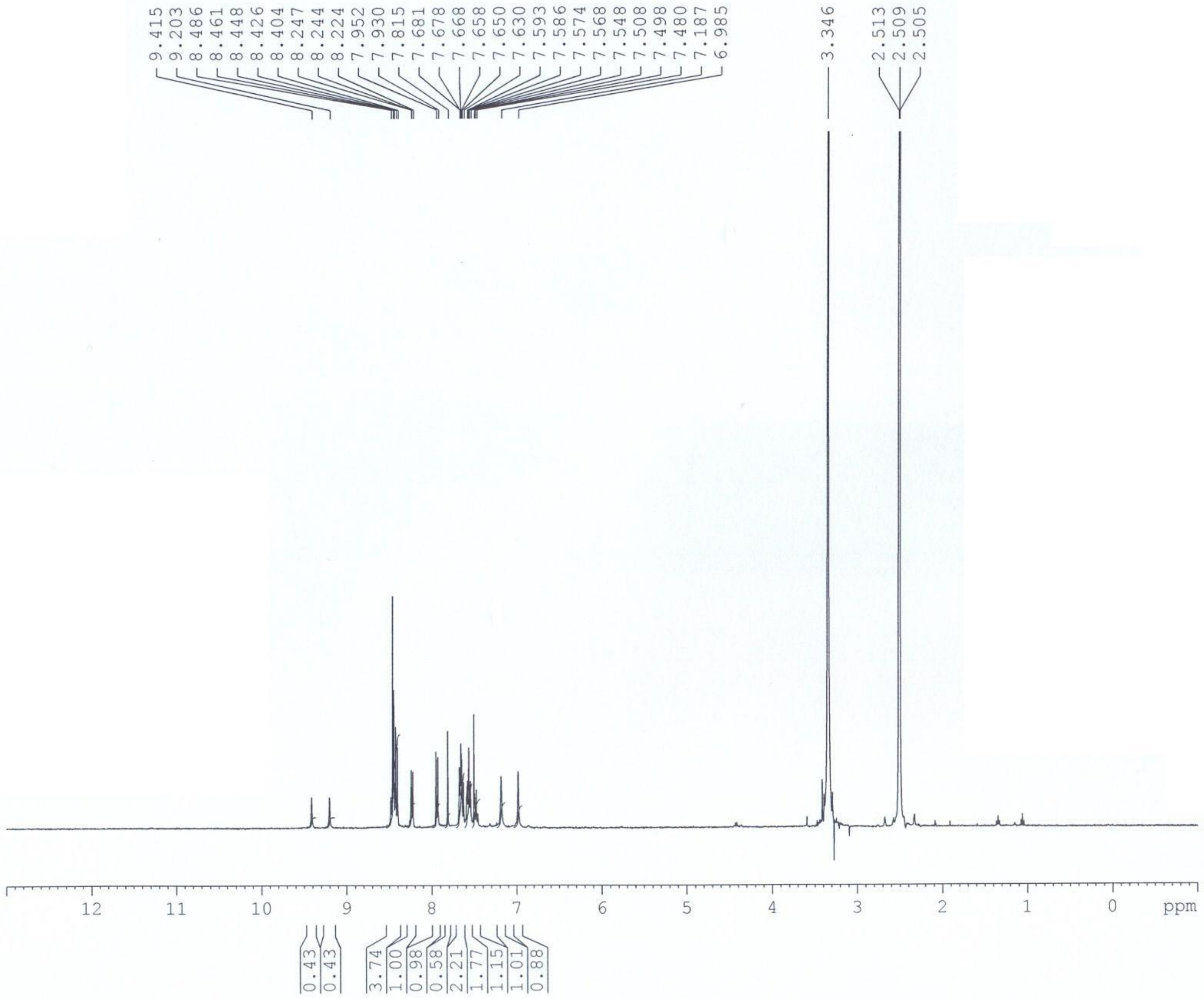

Compound 12f

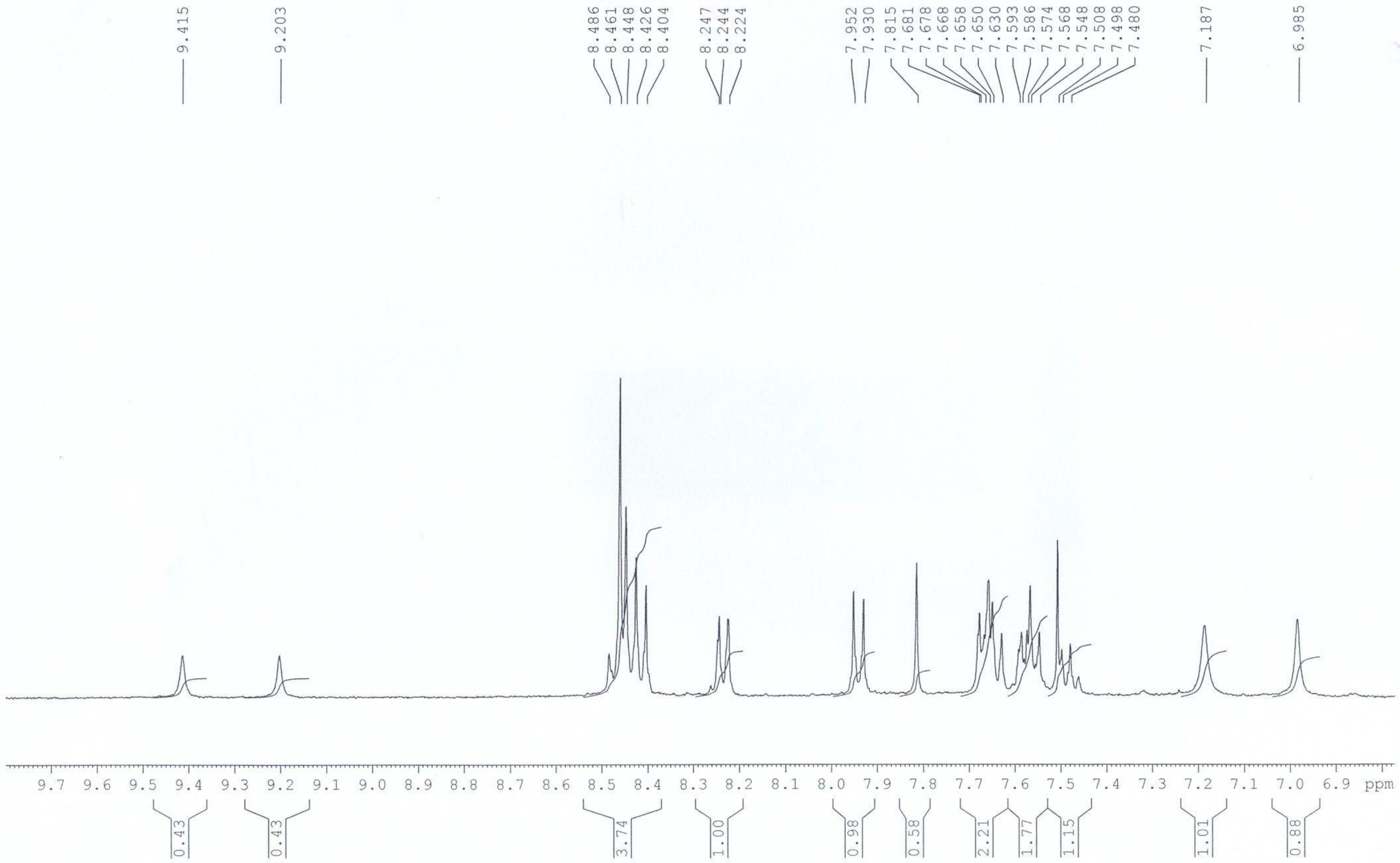

Compound 12f

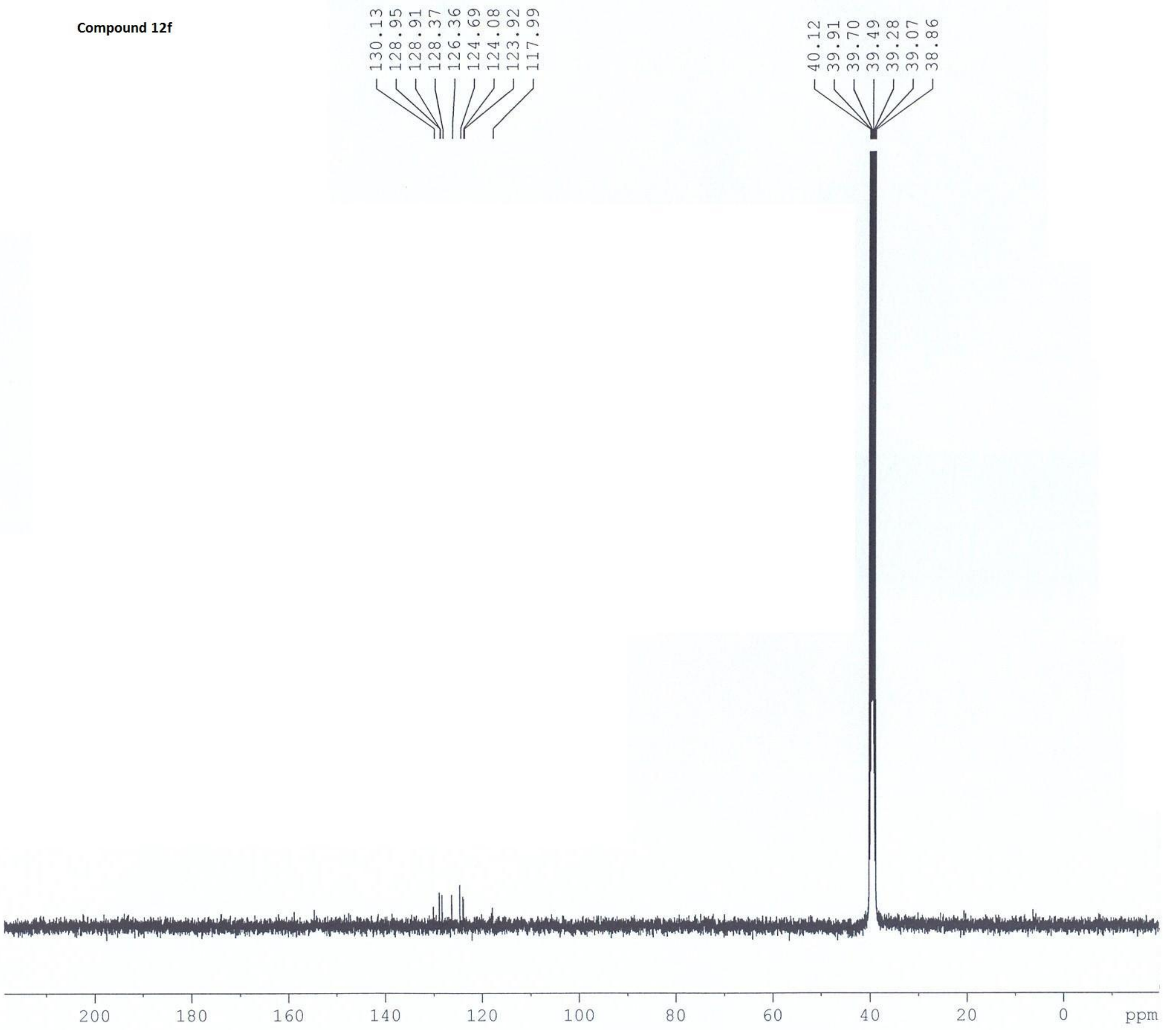

Compound 12f

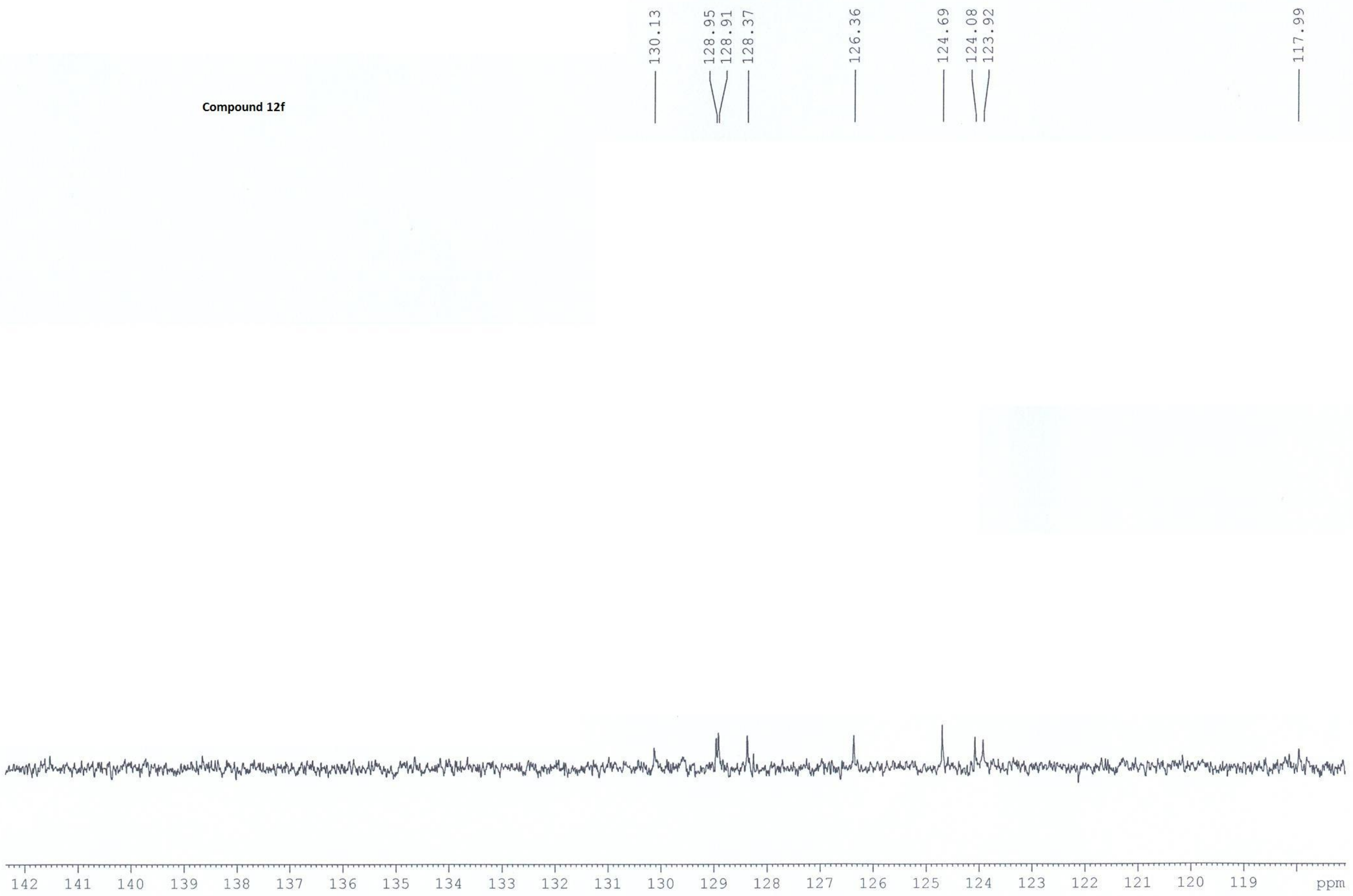

Compound 12f

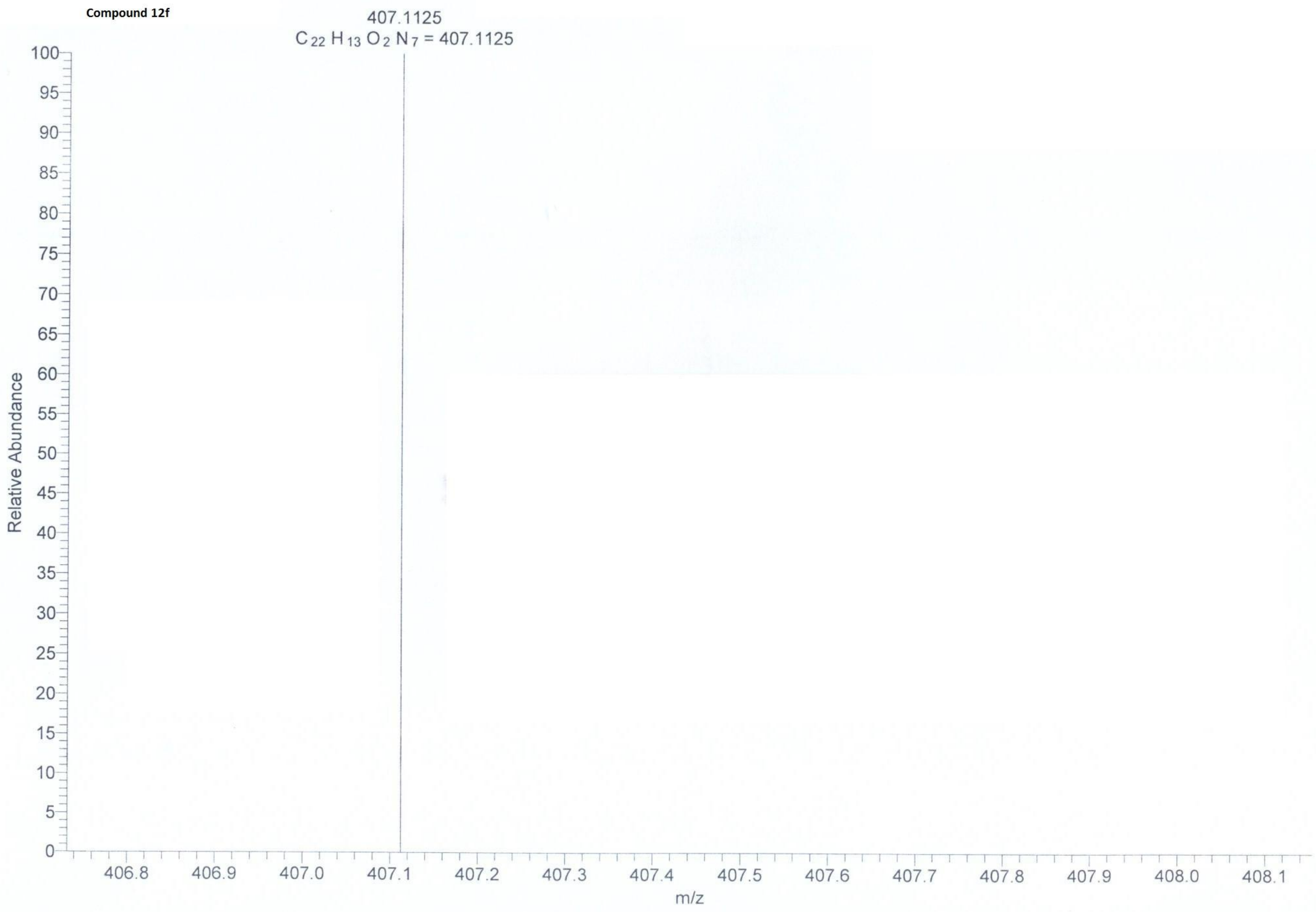

Compound 12g

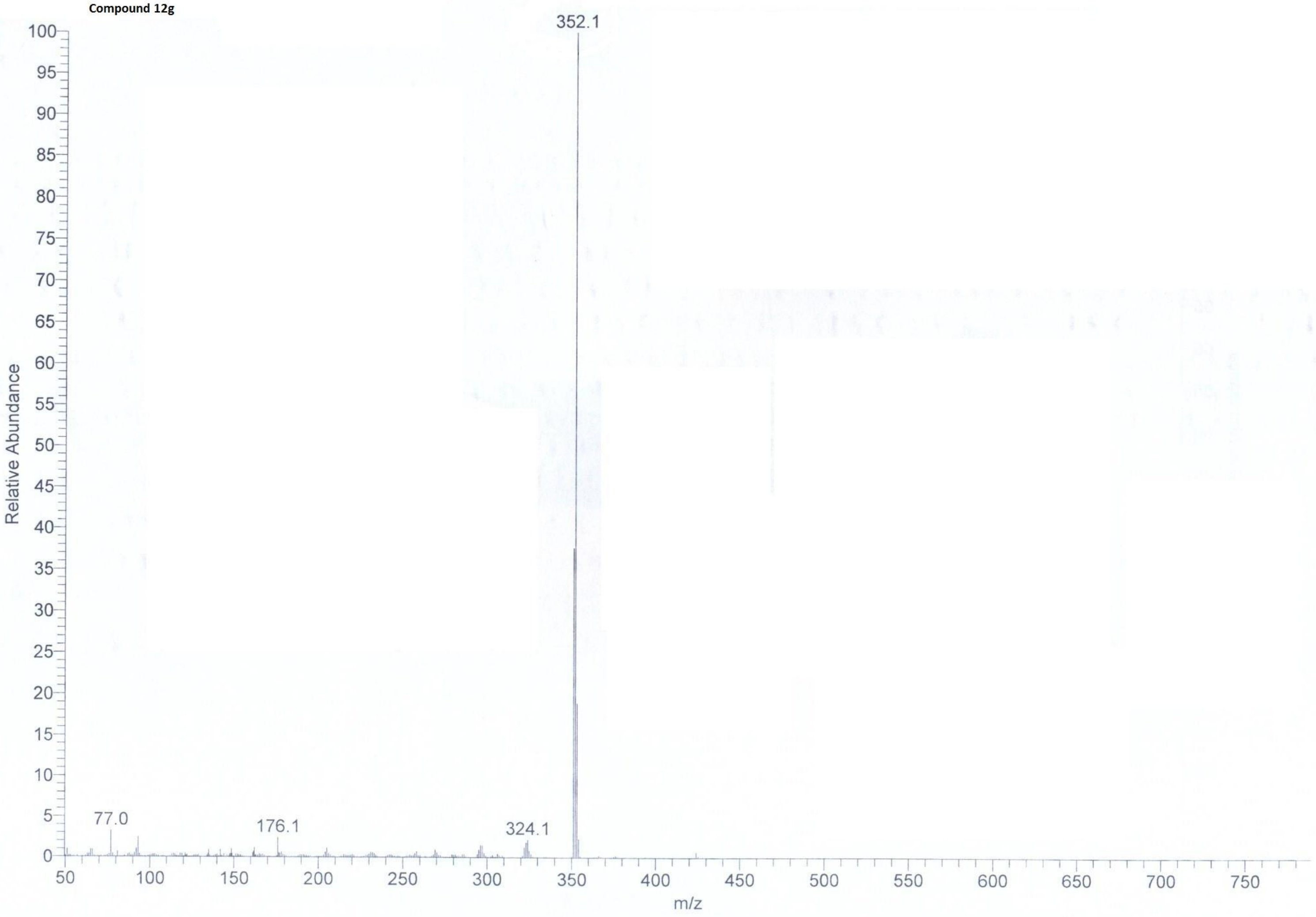

Compound 12g

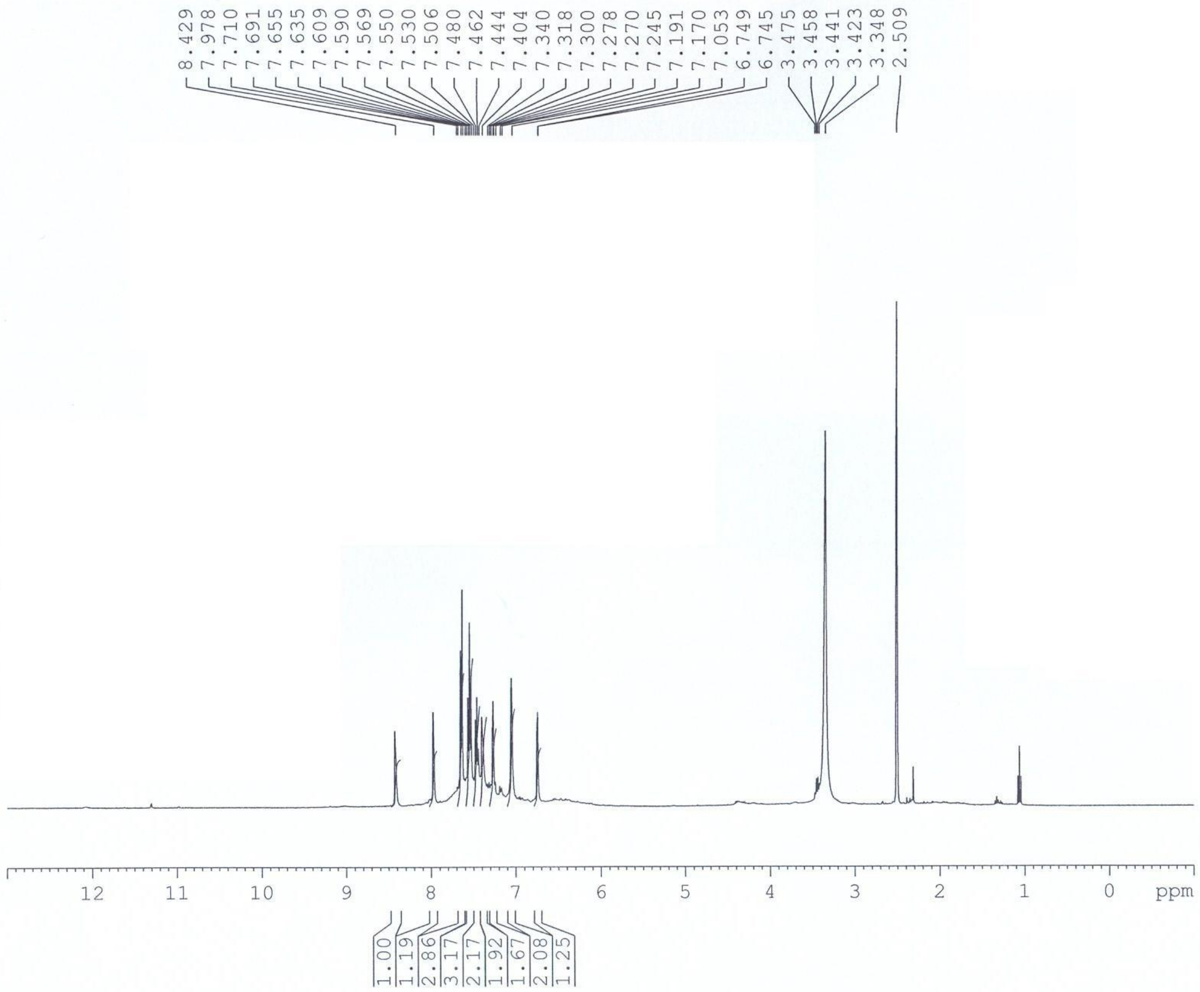

Compound 12g

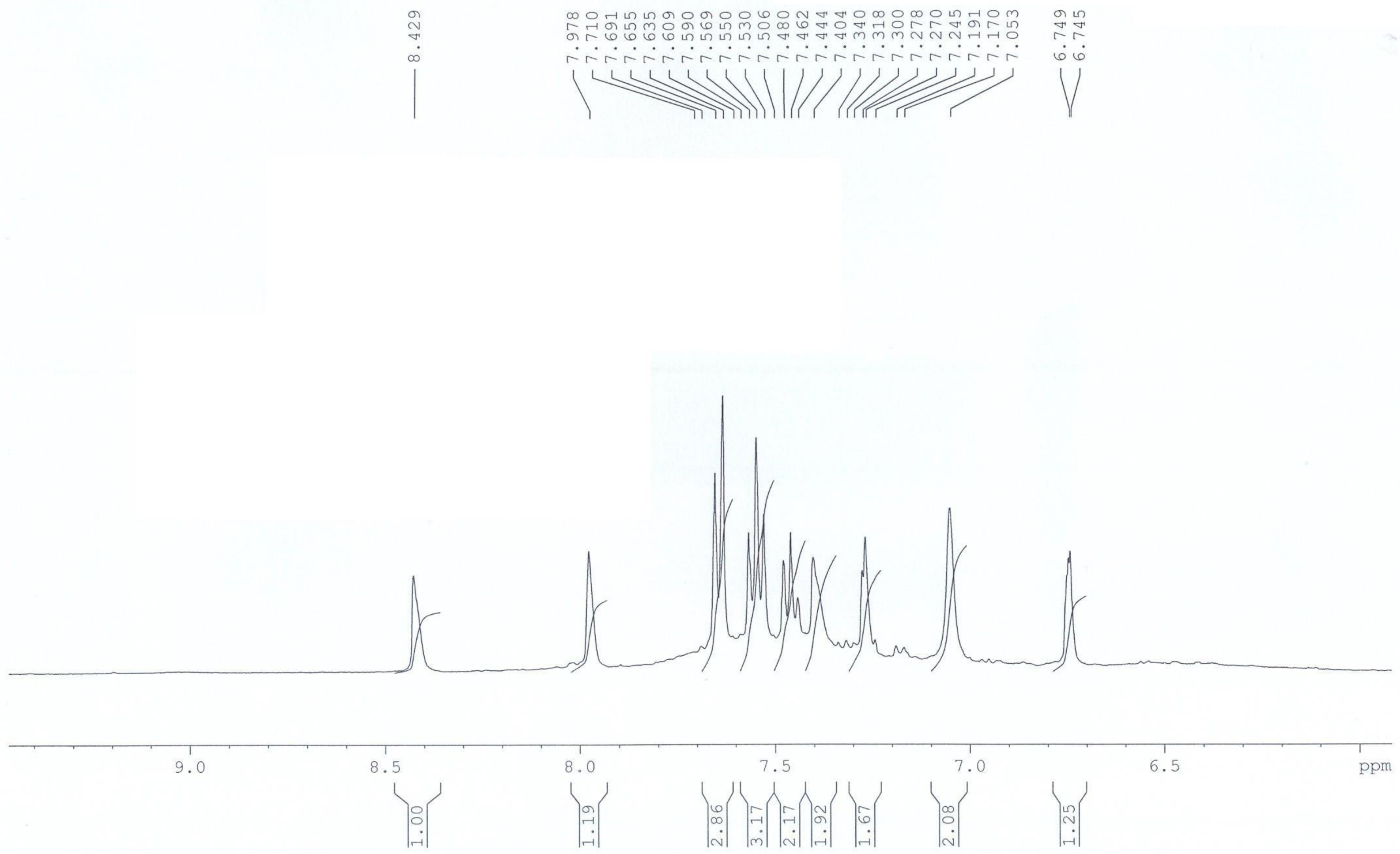

Compound 12g

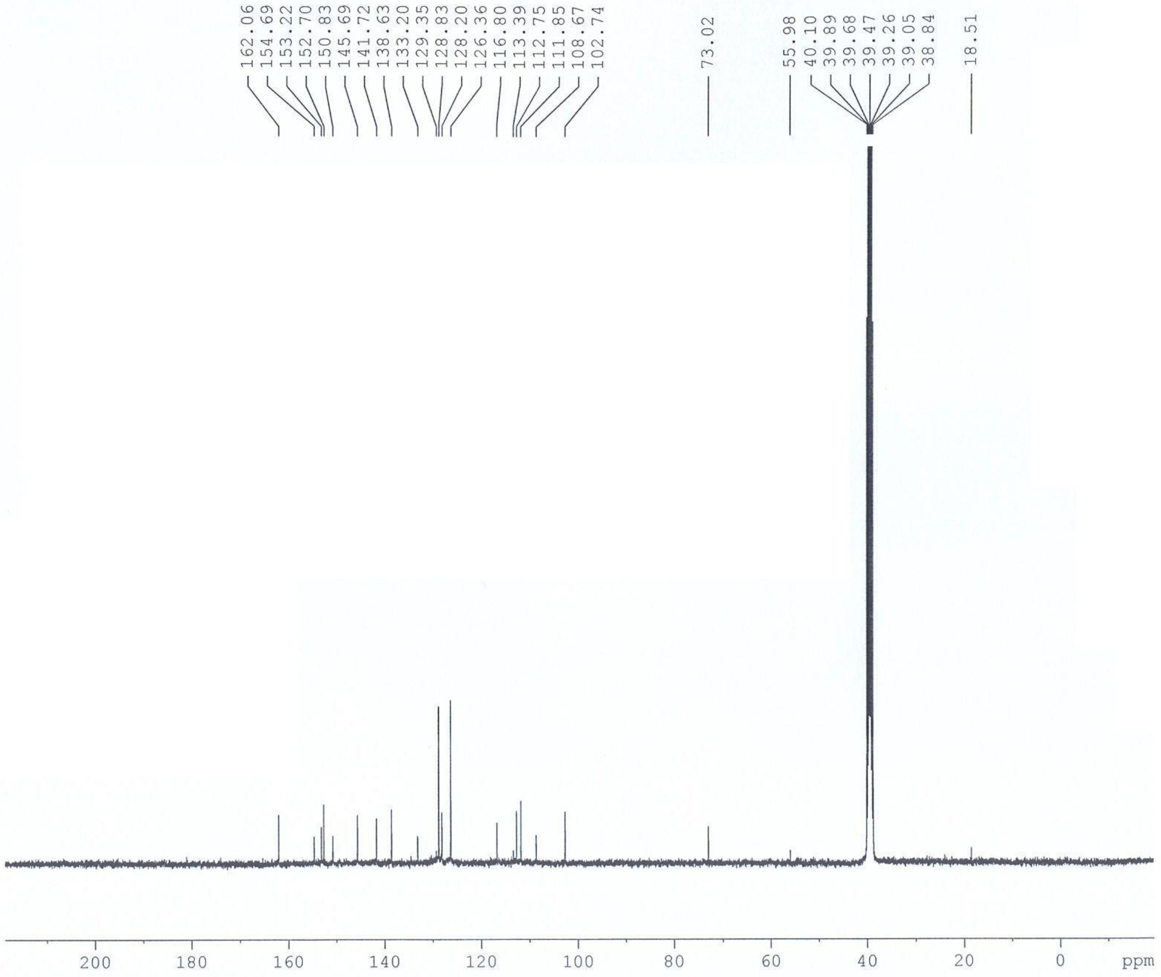

Compound 12g

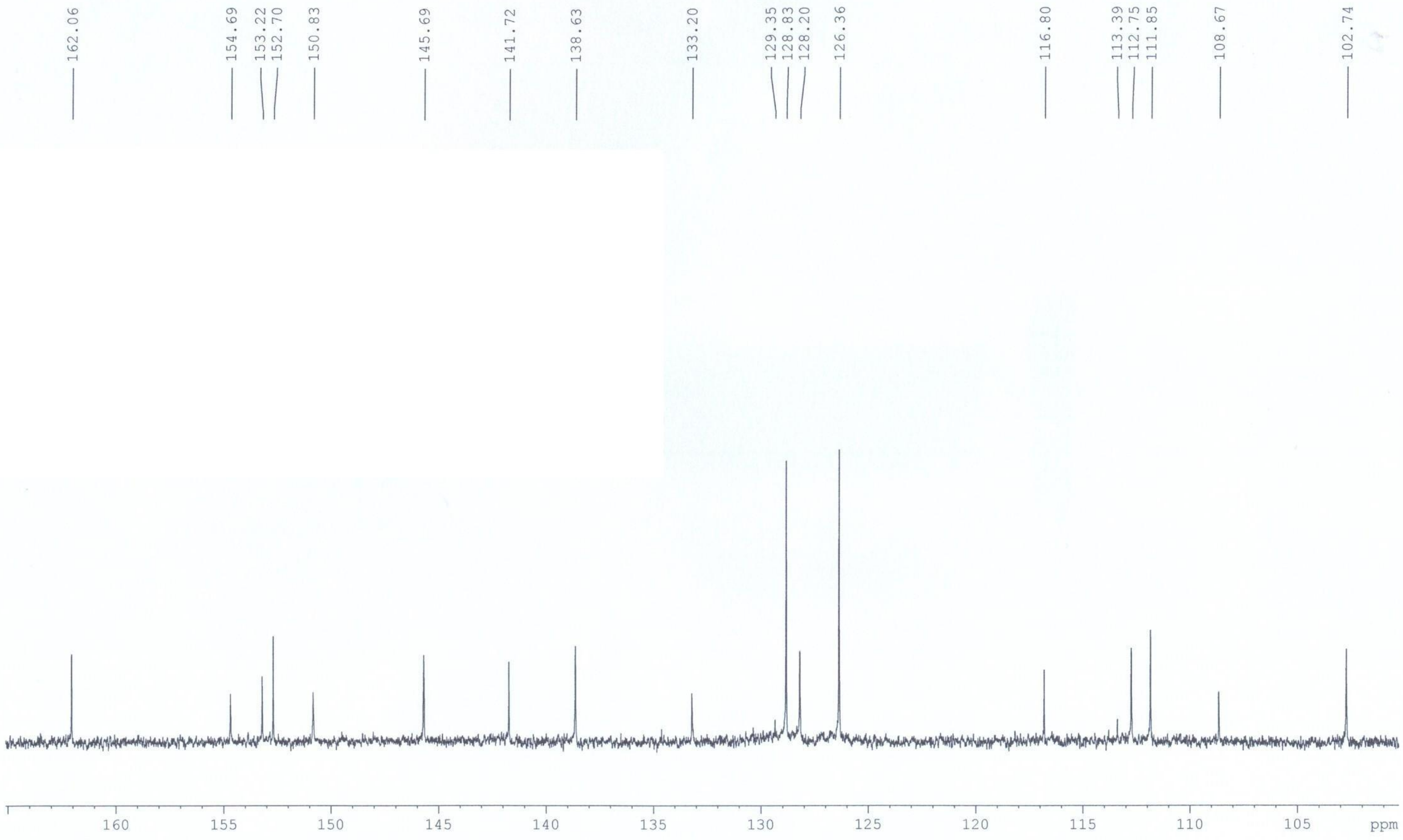

Compound 12g

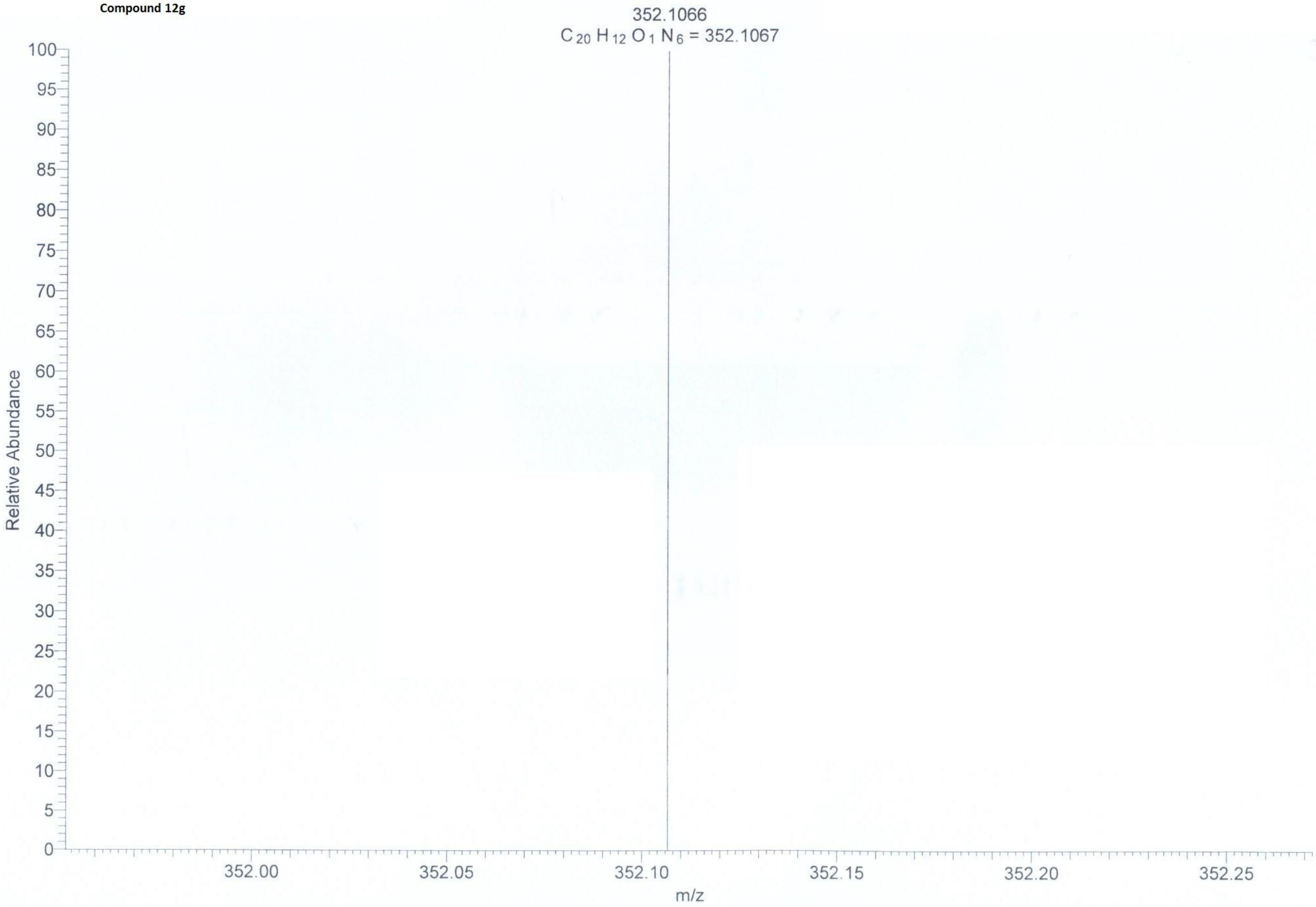

Compound 12h

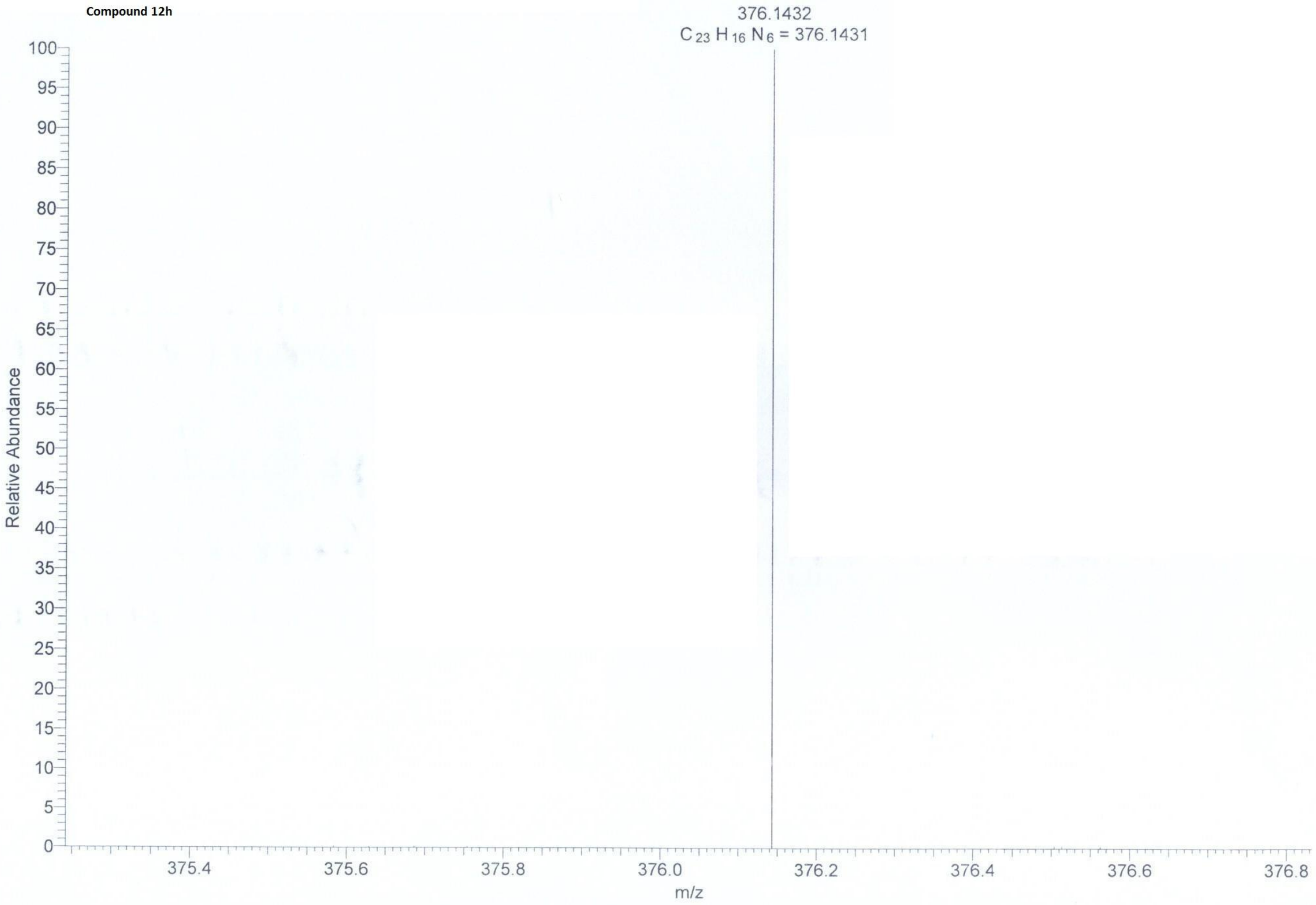

Compound 12h

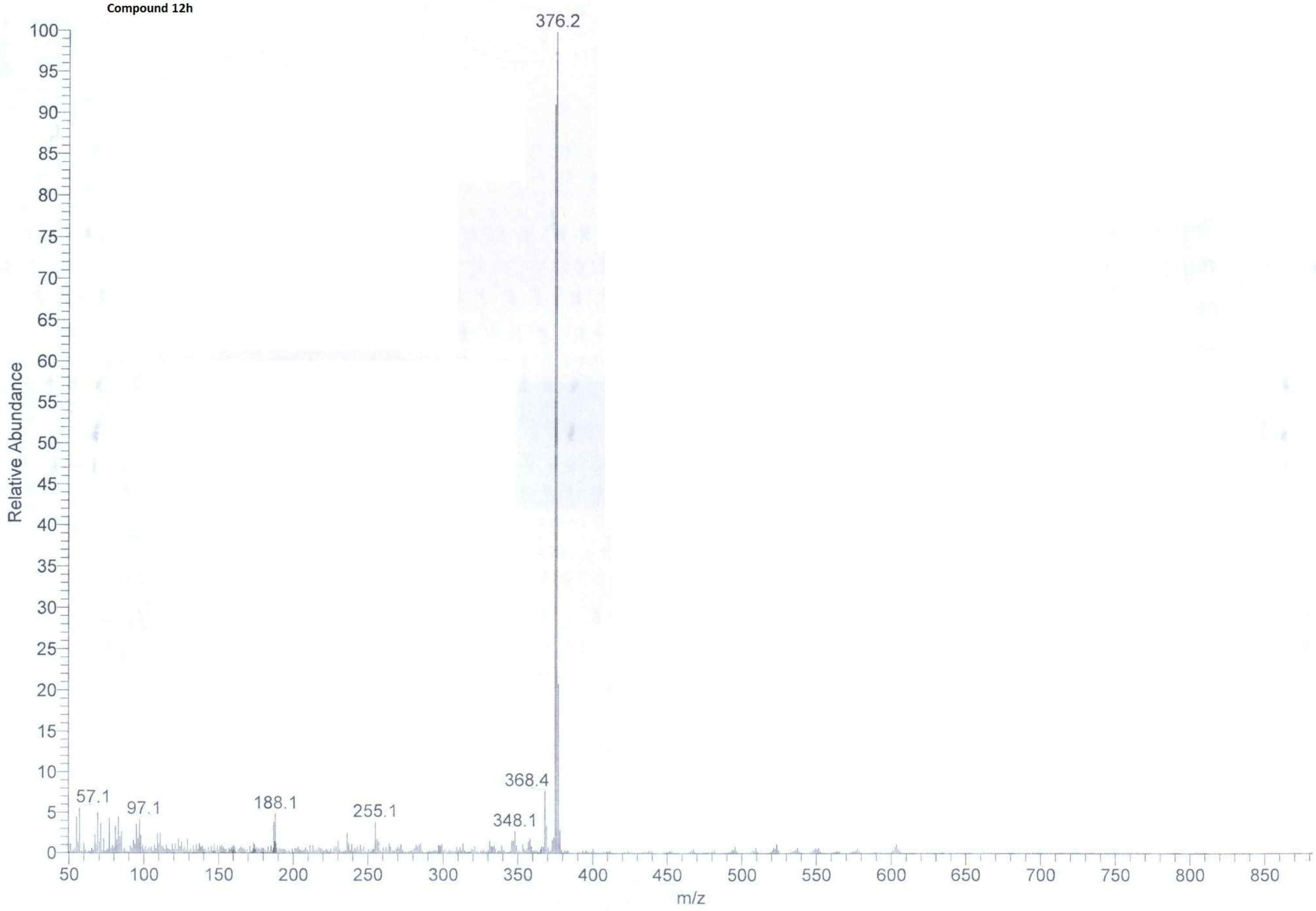

Compound 12h

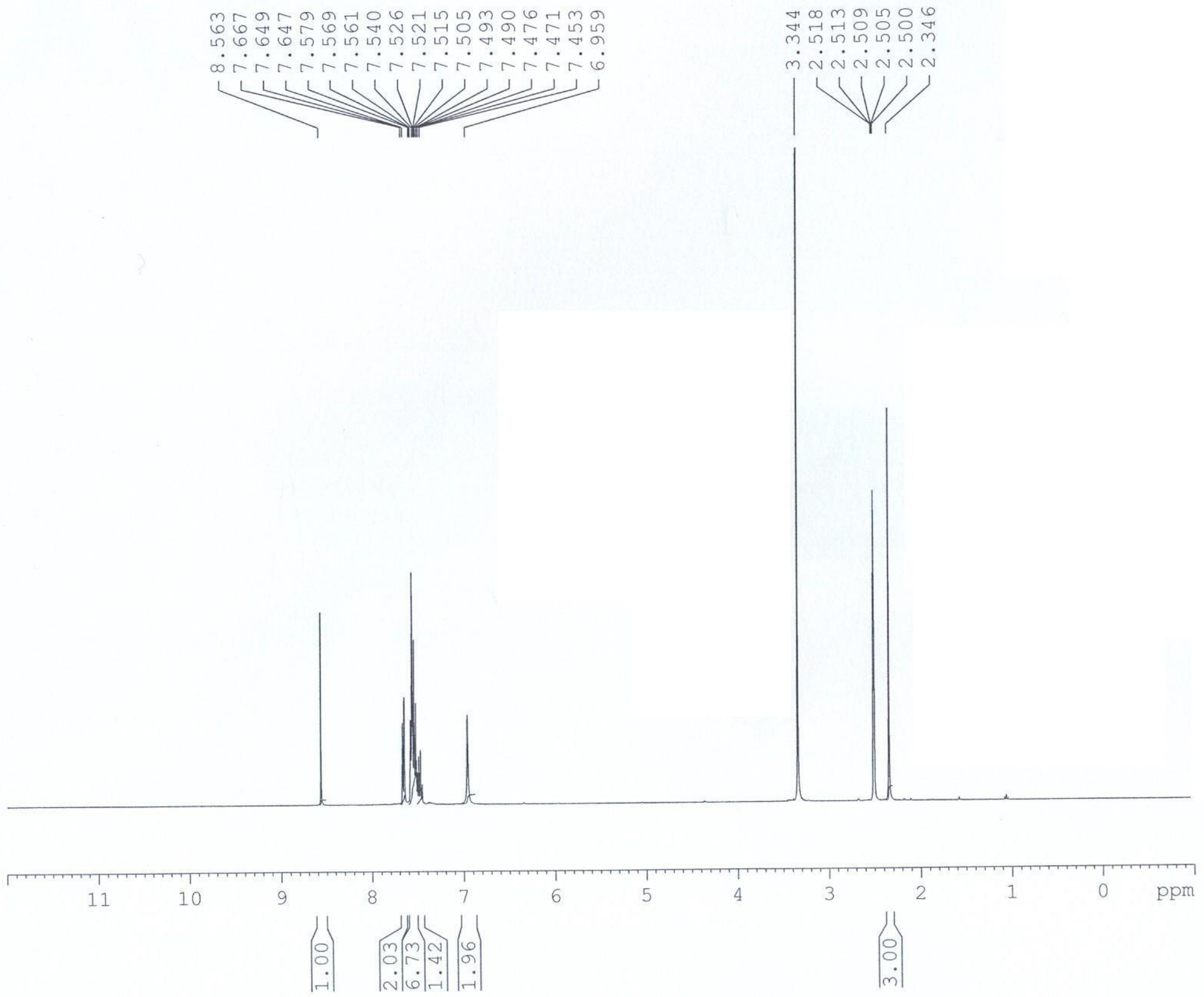

Compound 12h

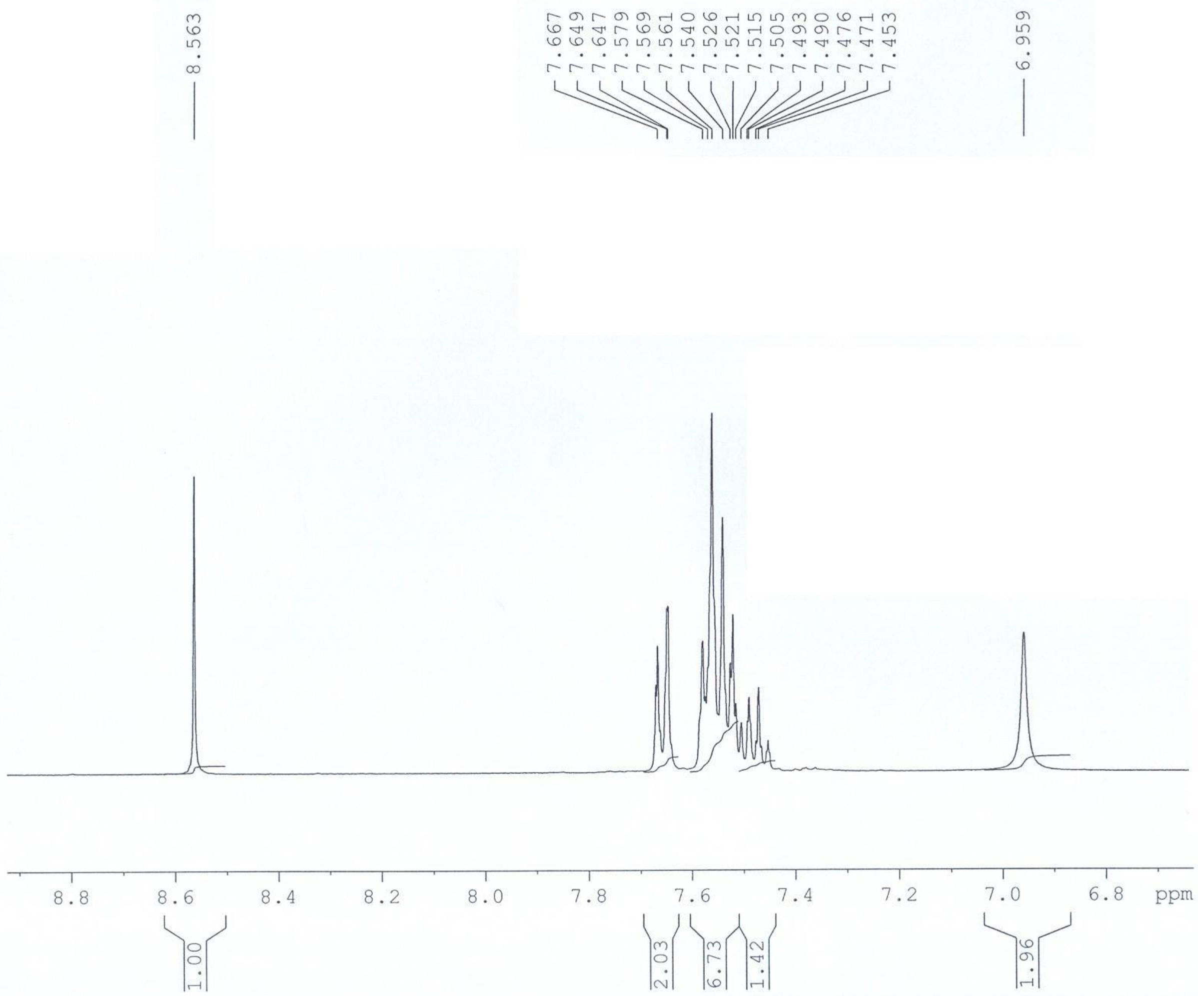

Compound 12h

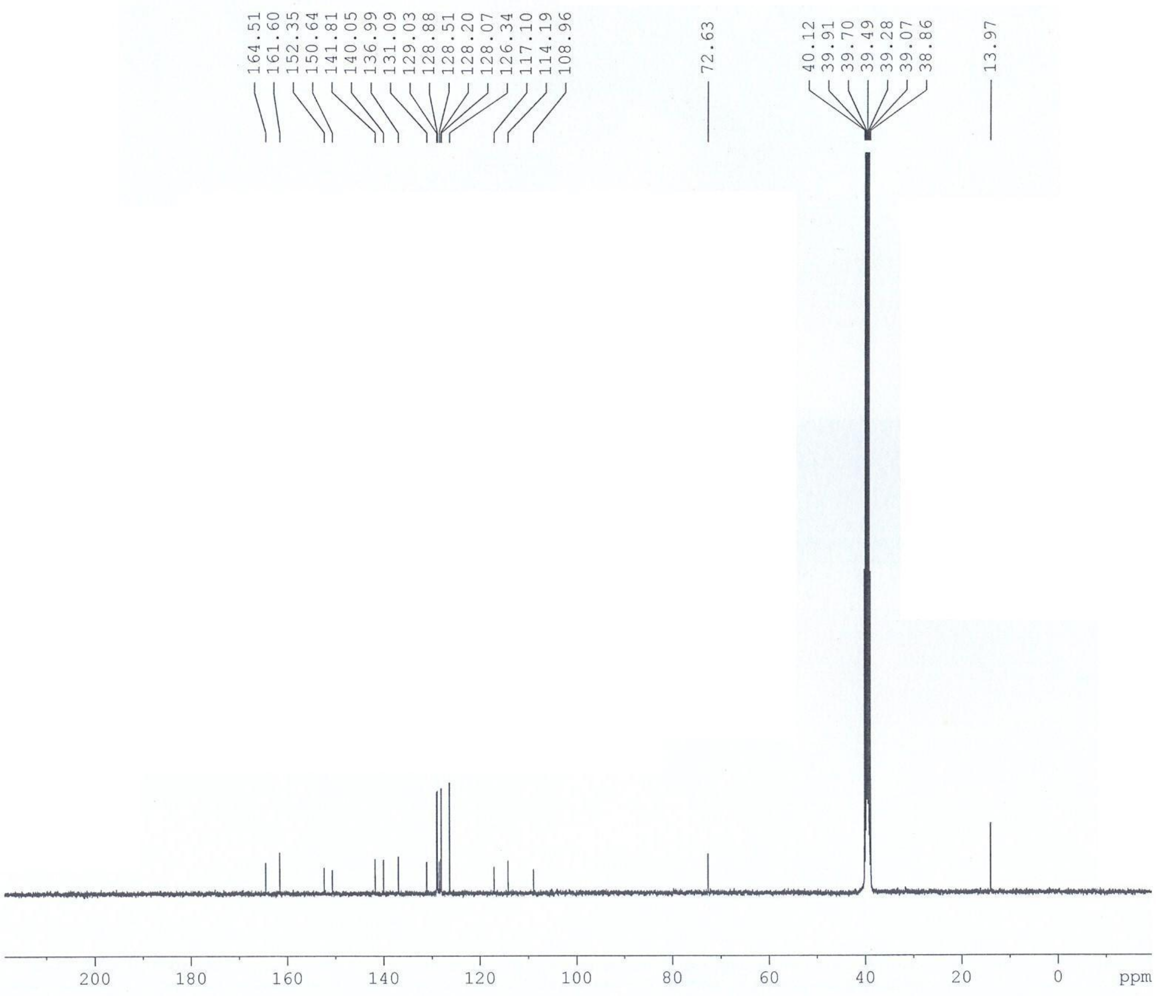

Compound 12h

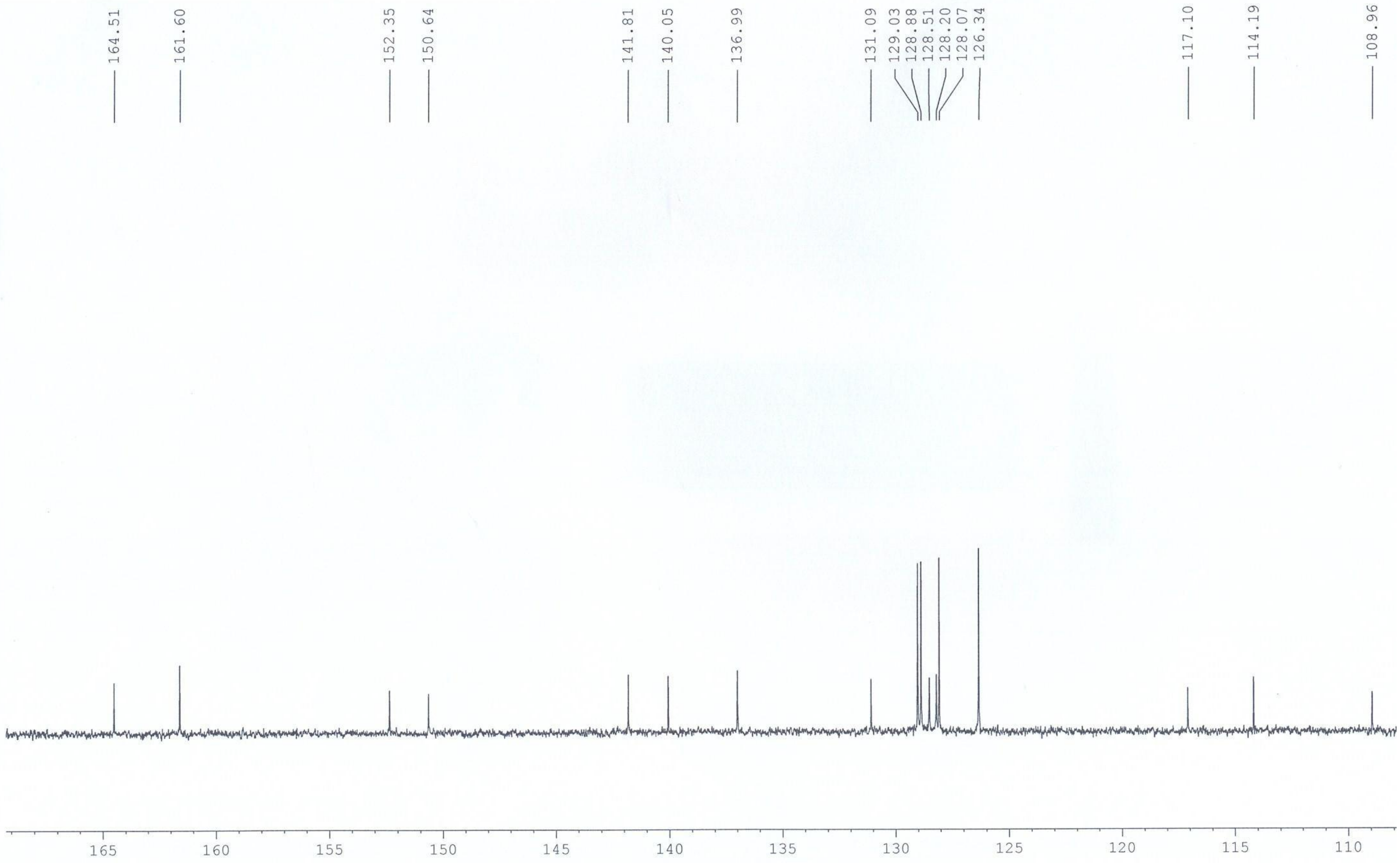

Compound 12j

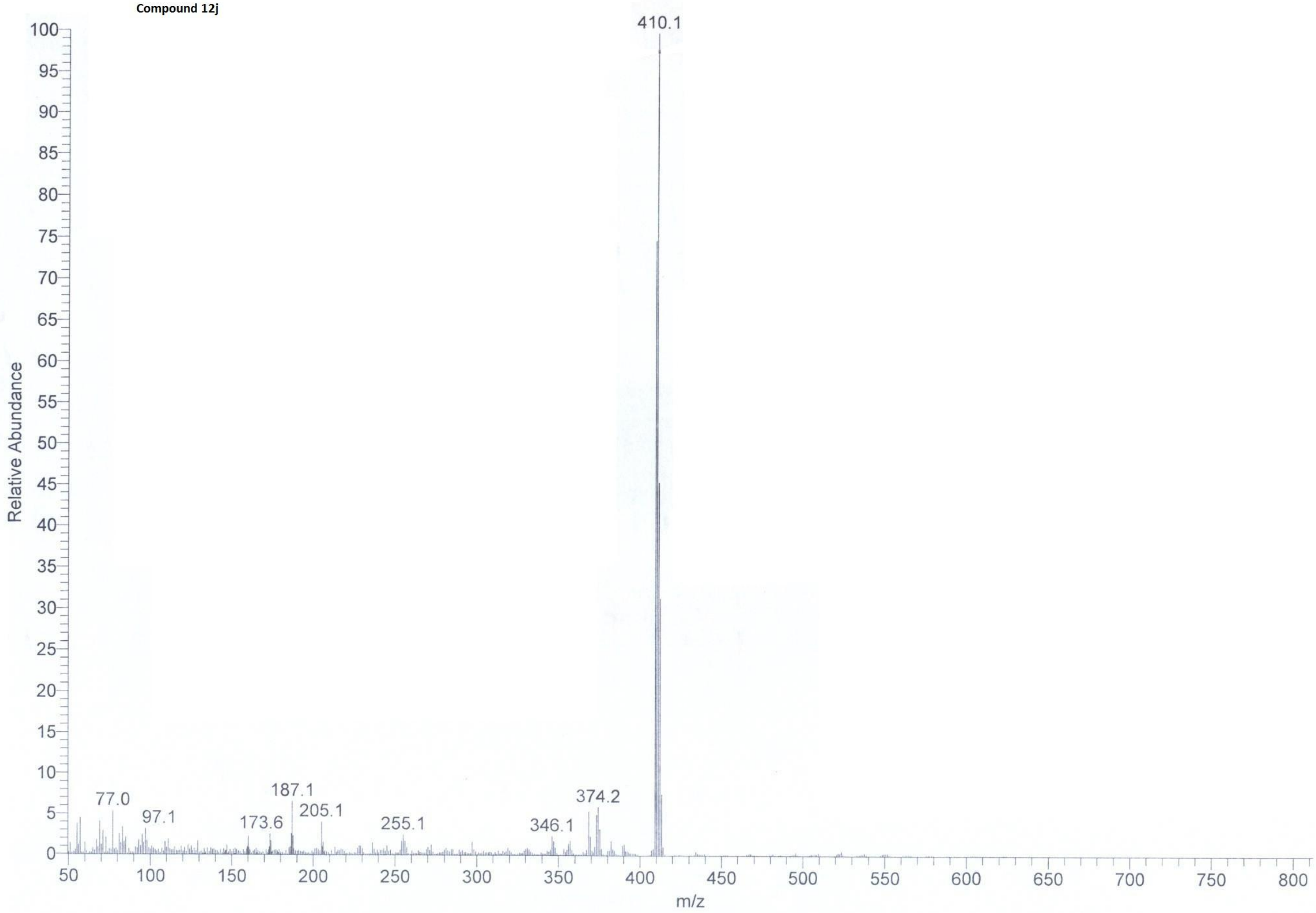

Compound 12j

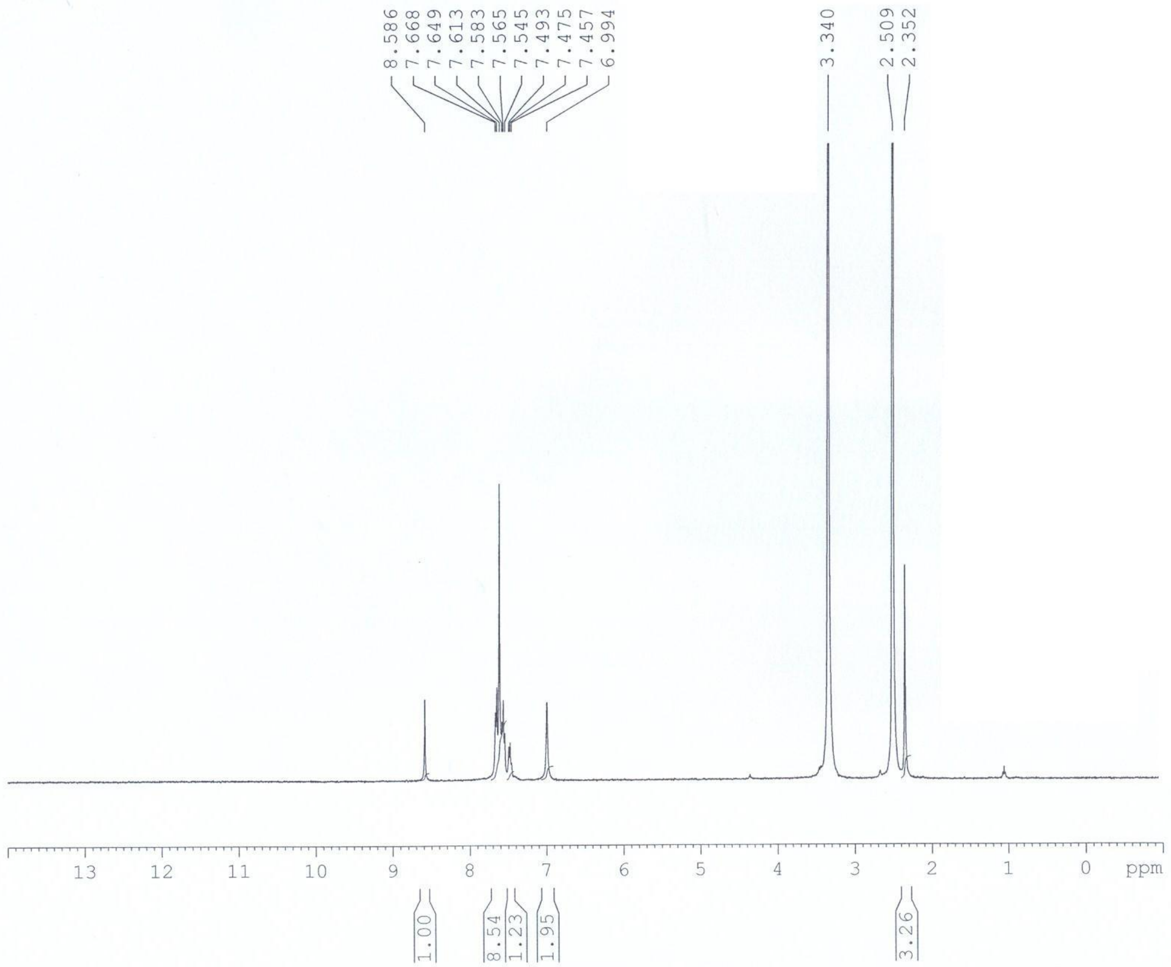

Compound 12j

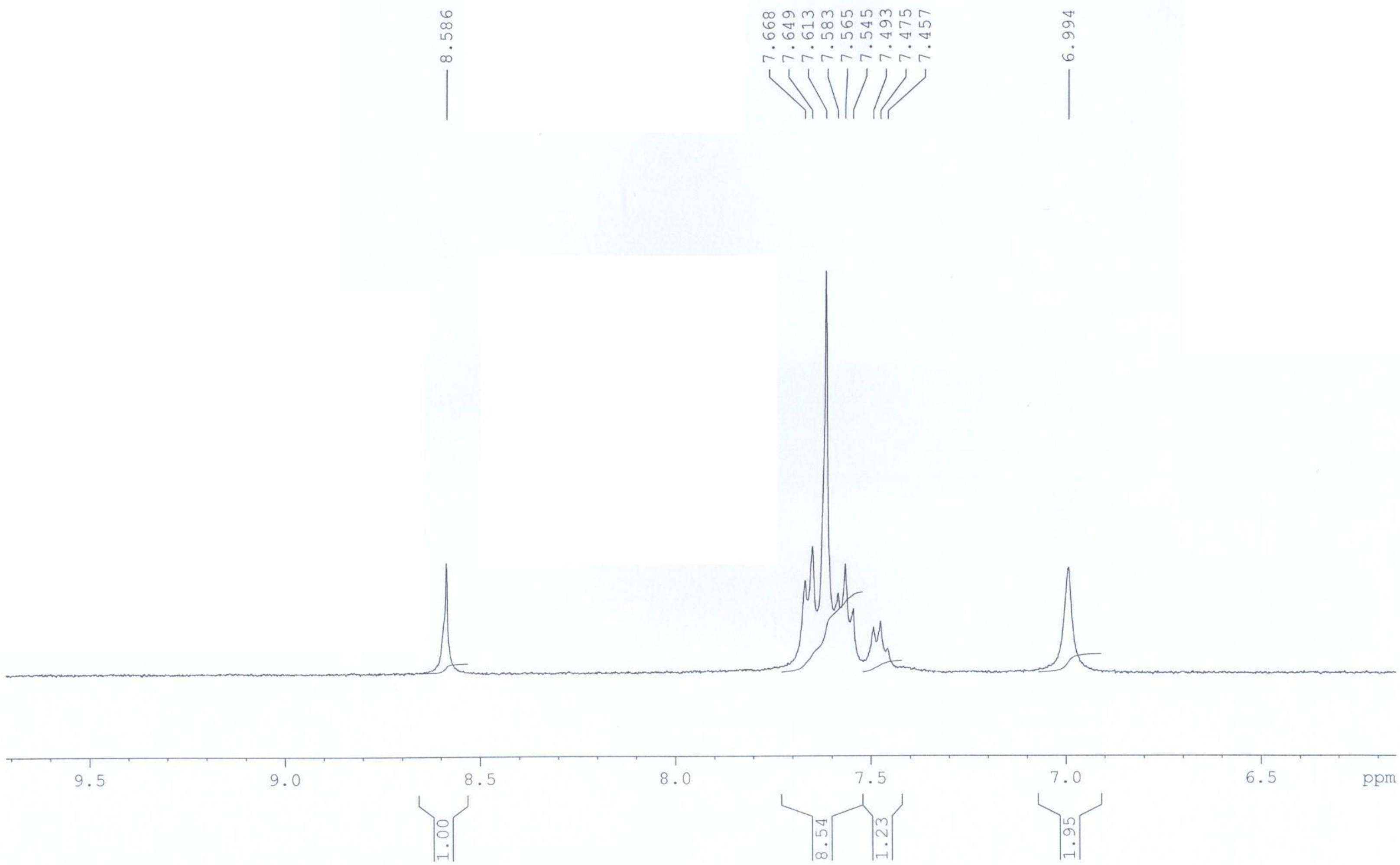

Compound 12j

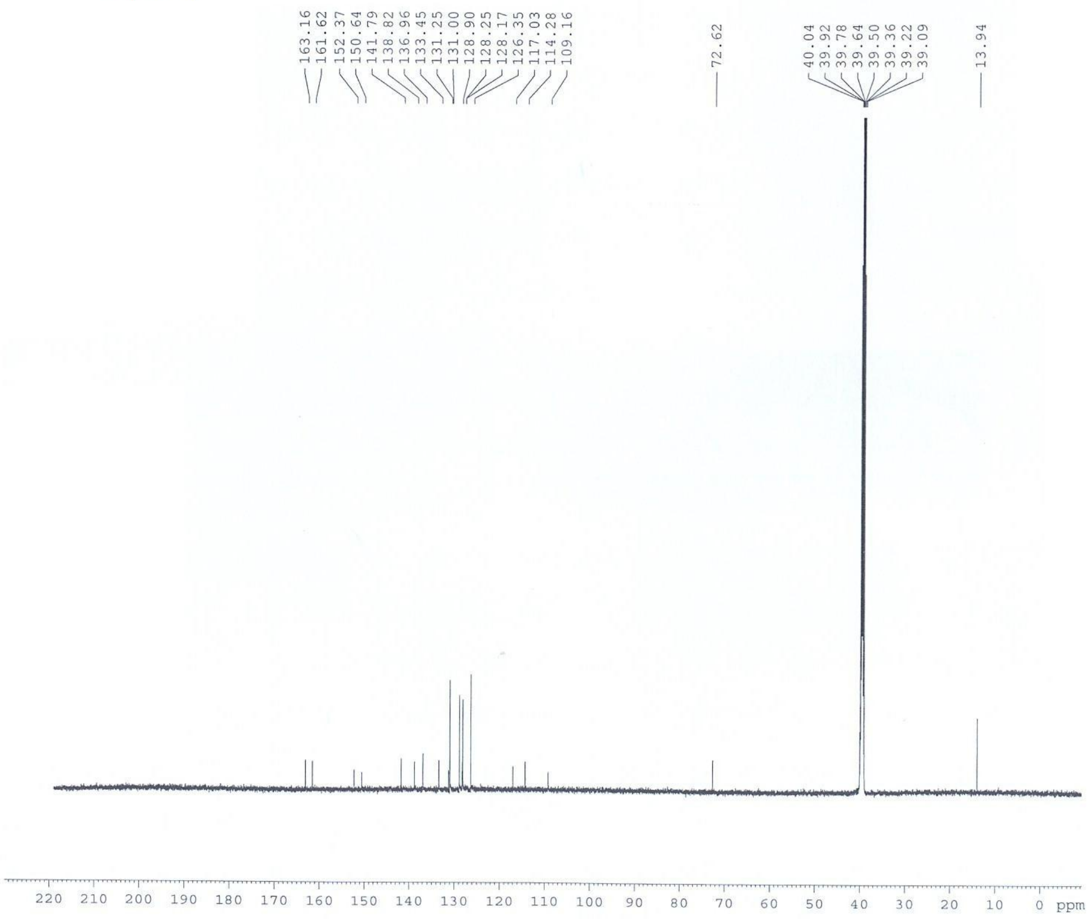

Compound 12j

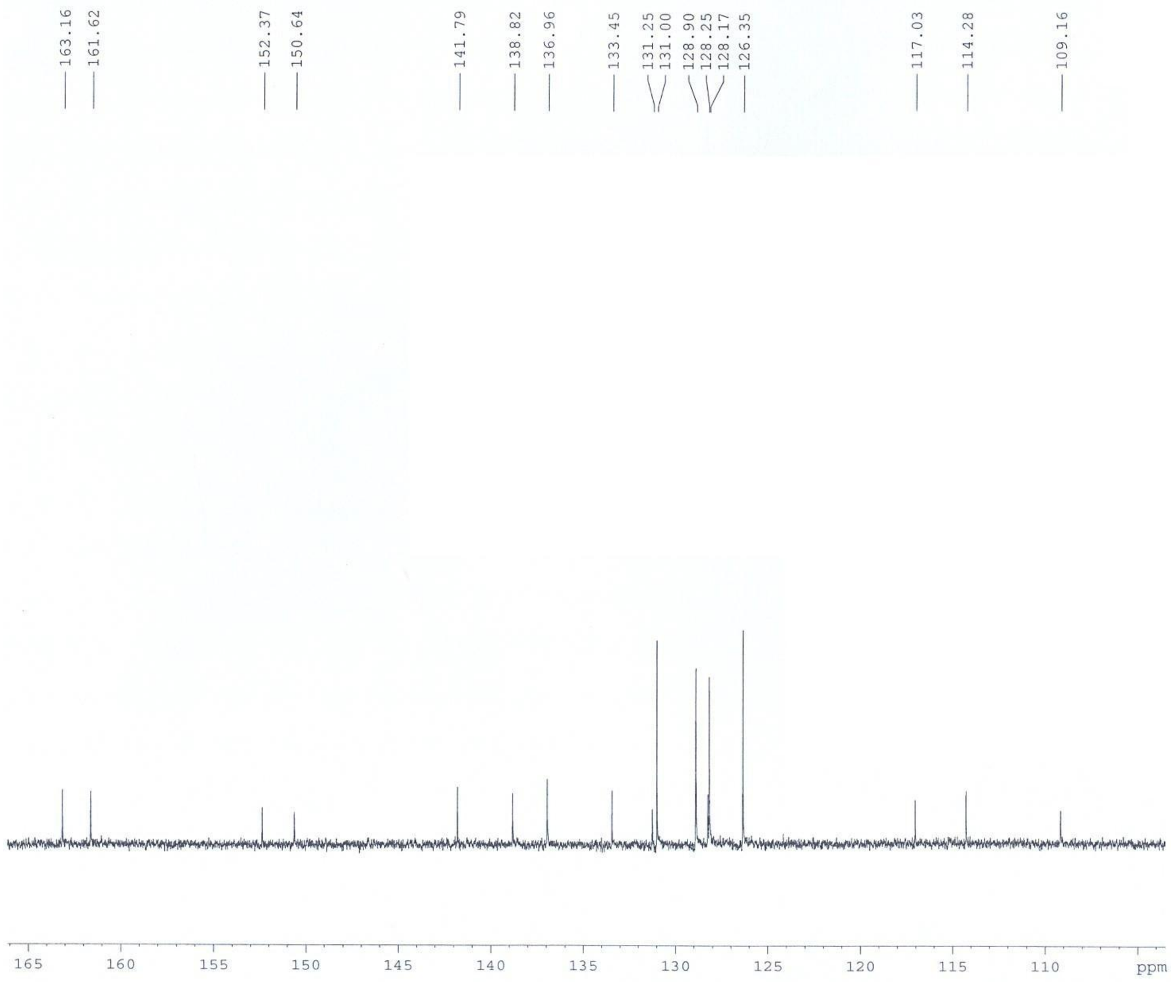

Compound 12j

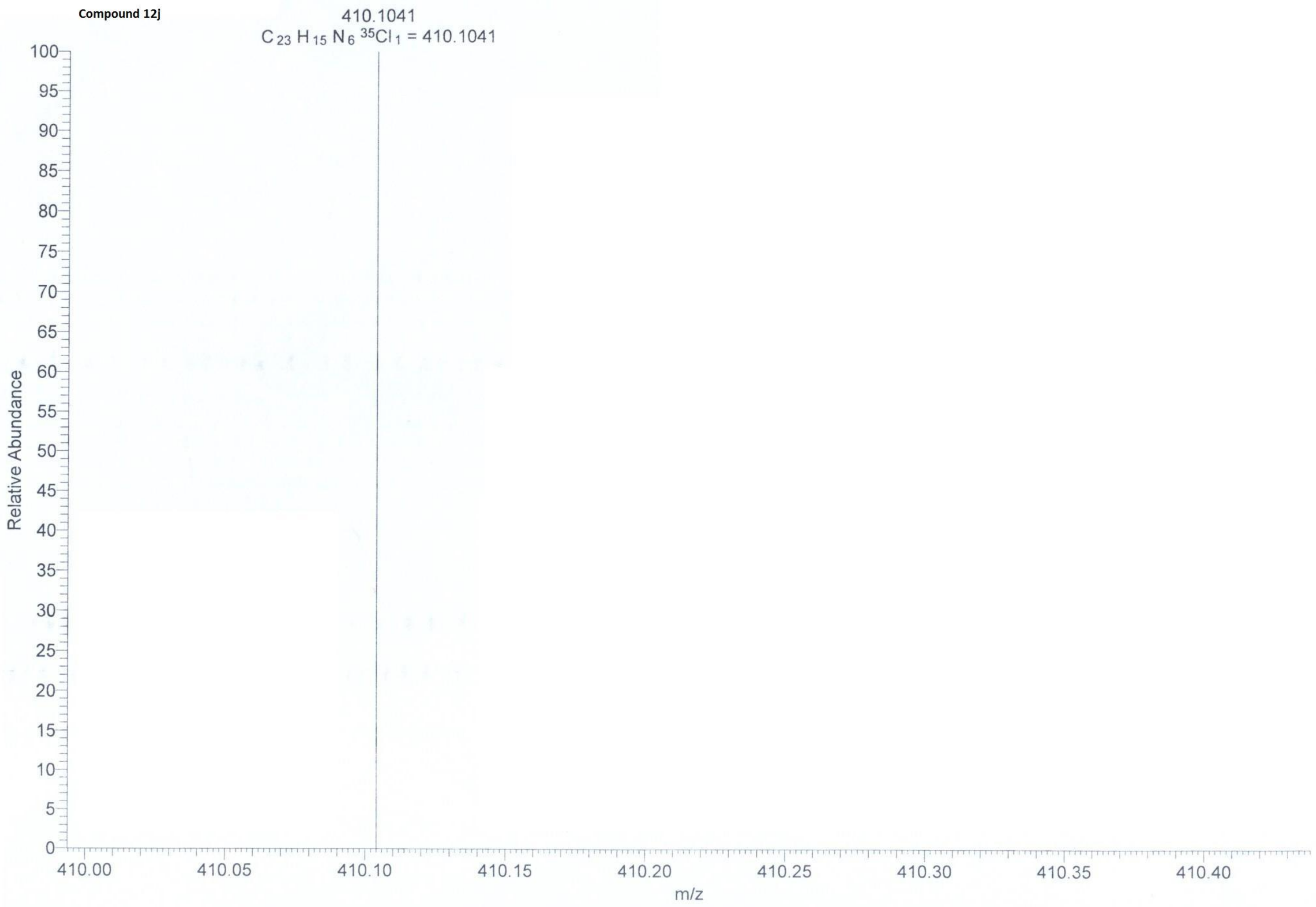

Compound 12k

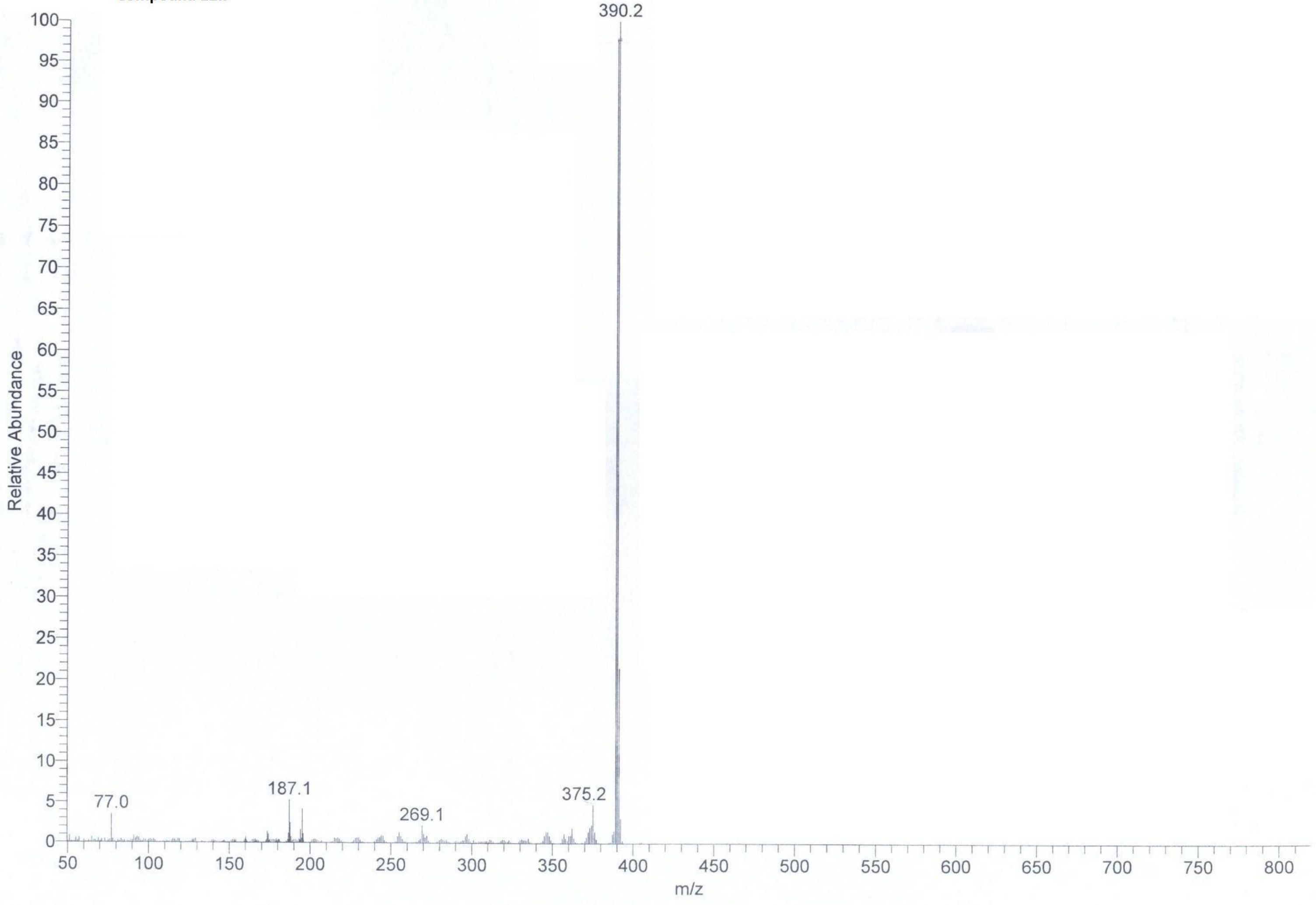

Compound 12k

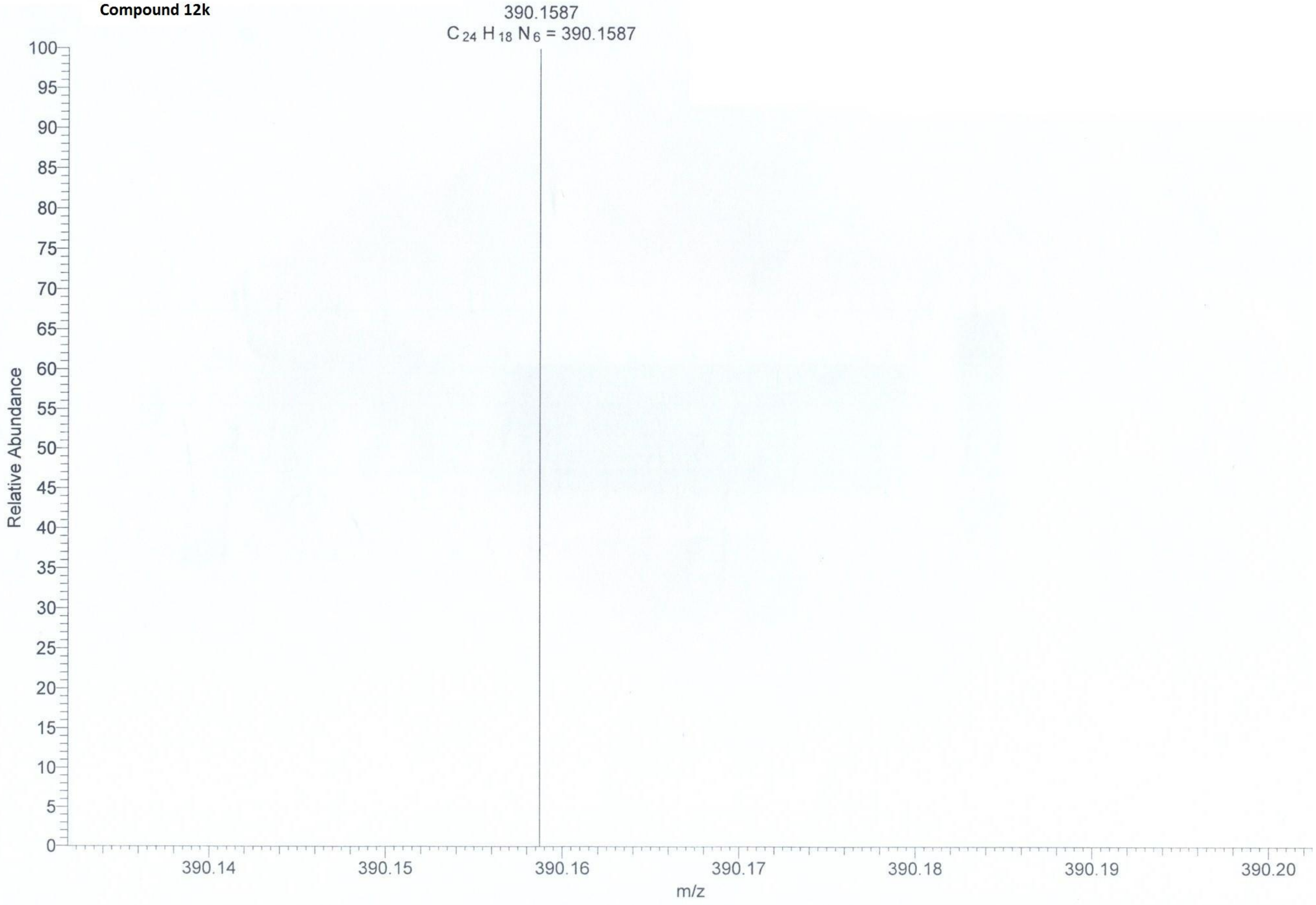

Compound 12k

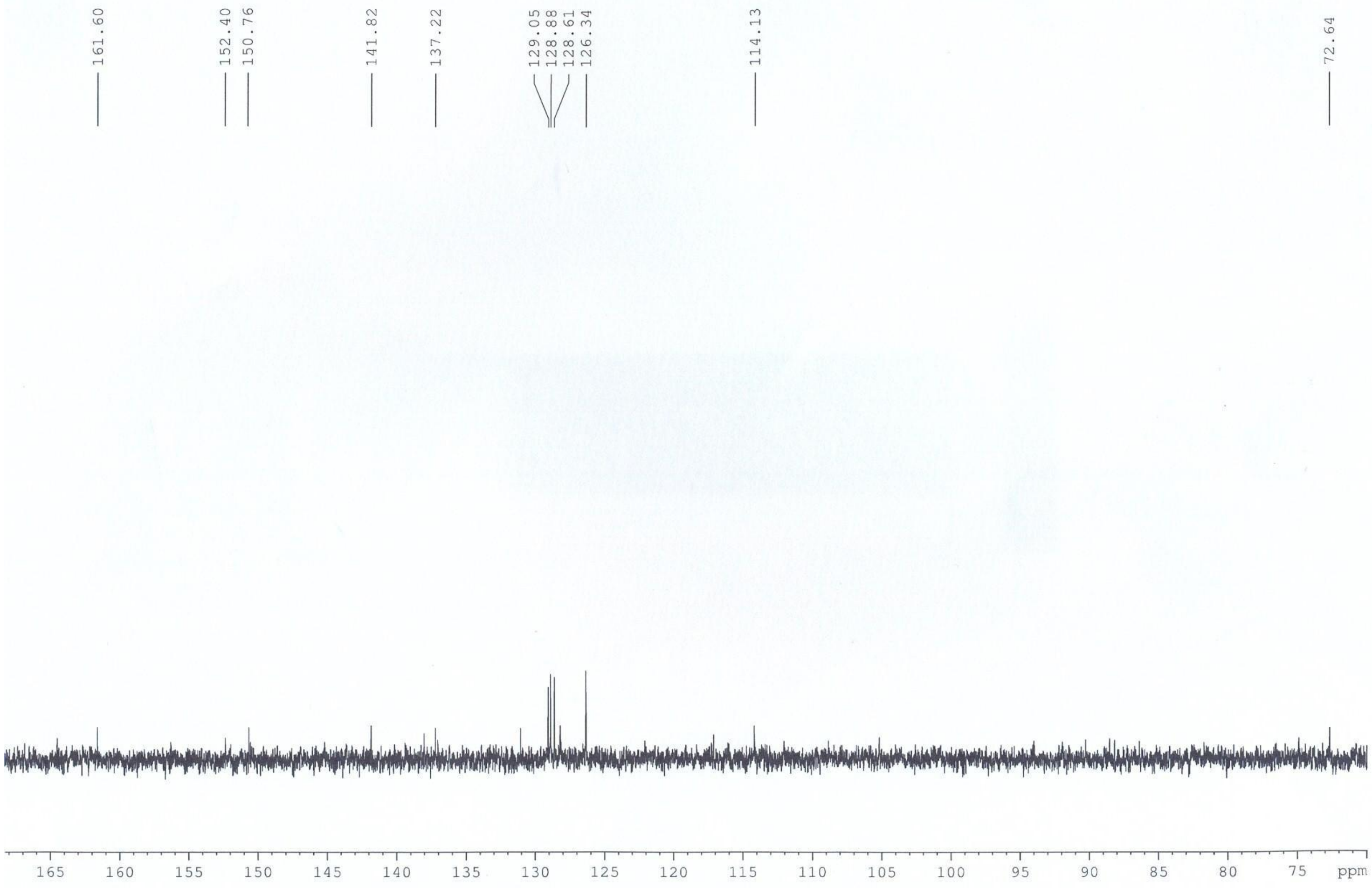

Compound 12k

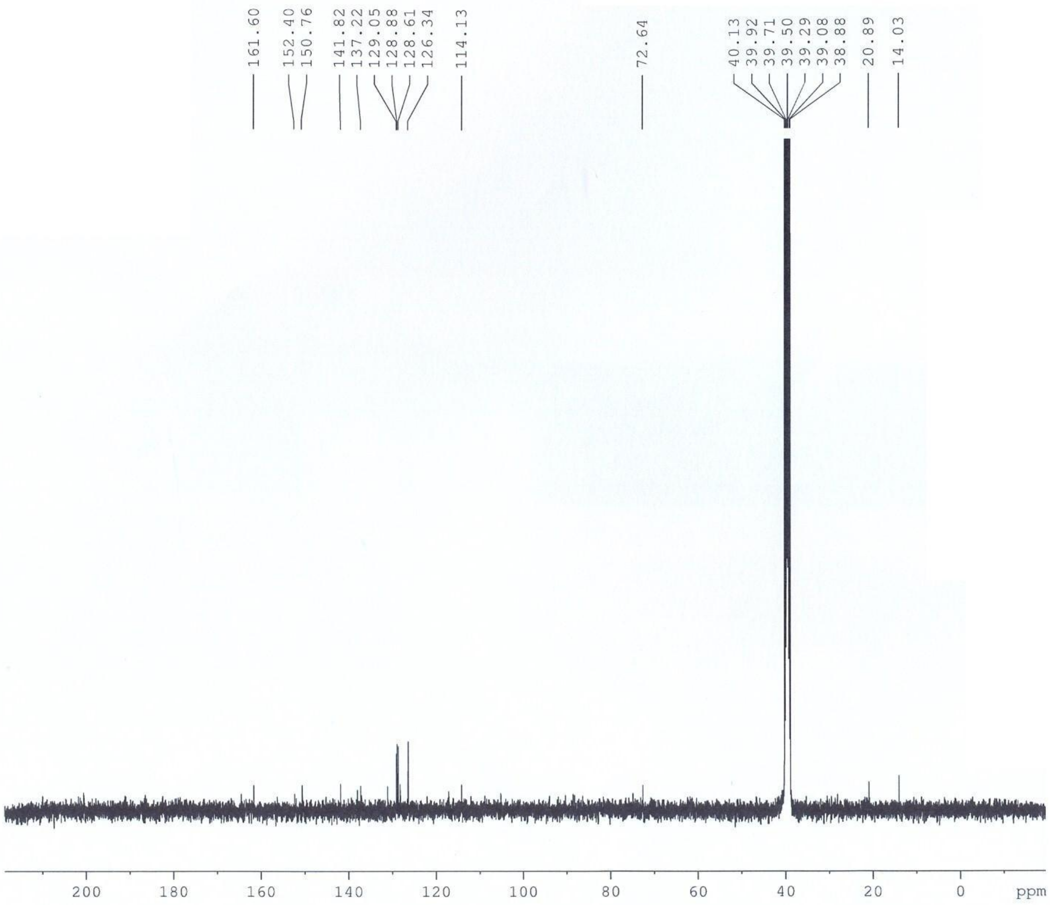

Compound 12k

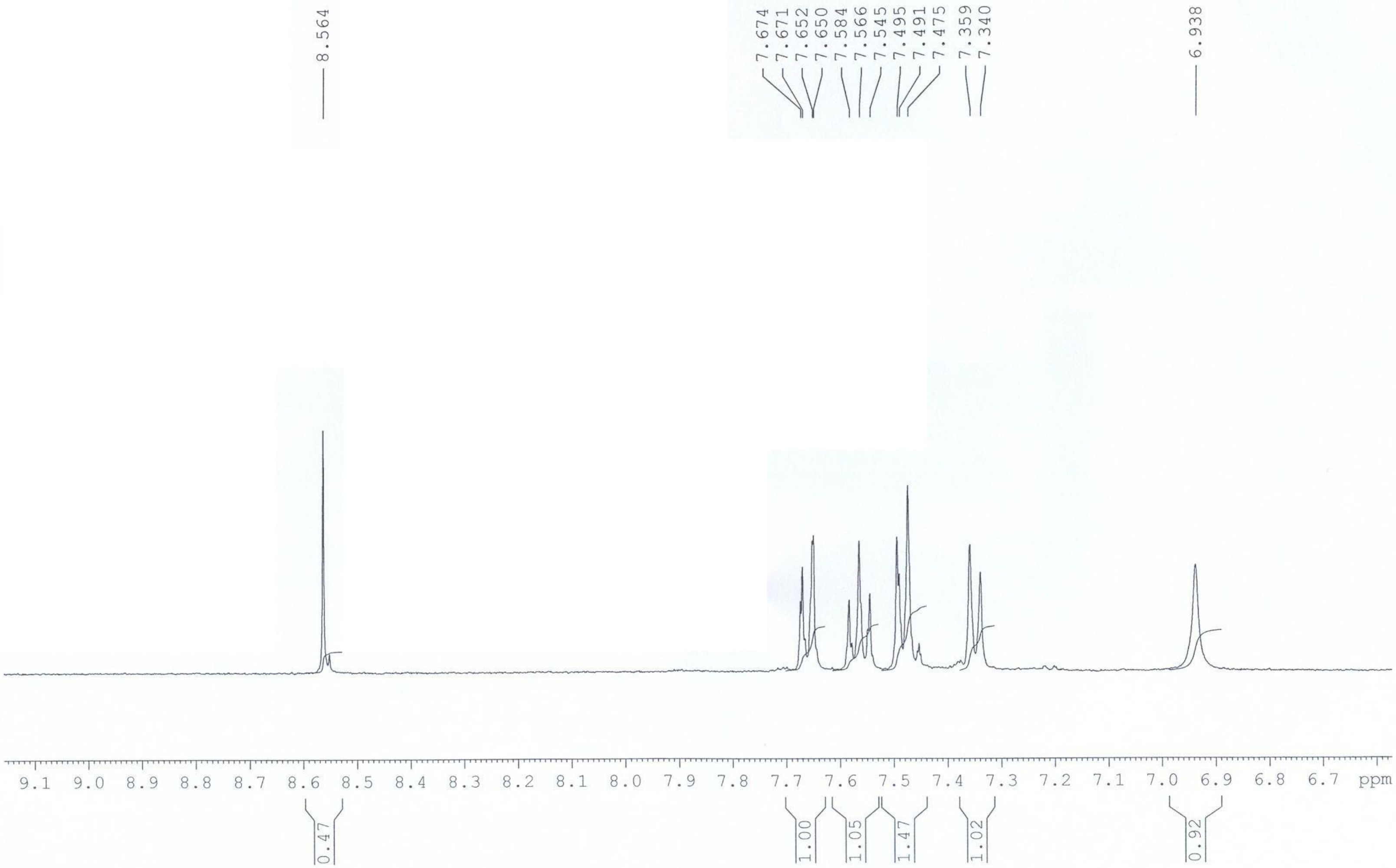

Compound 12k

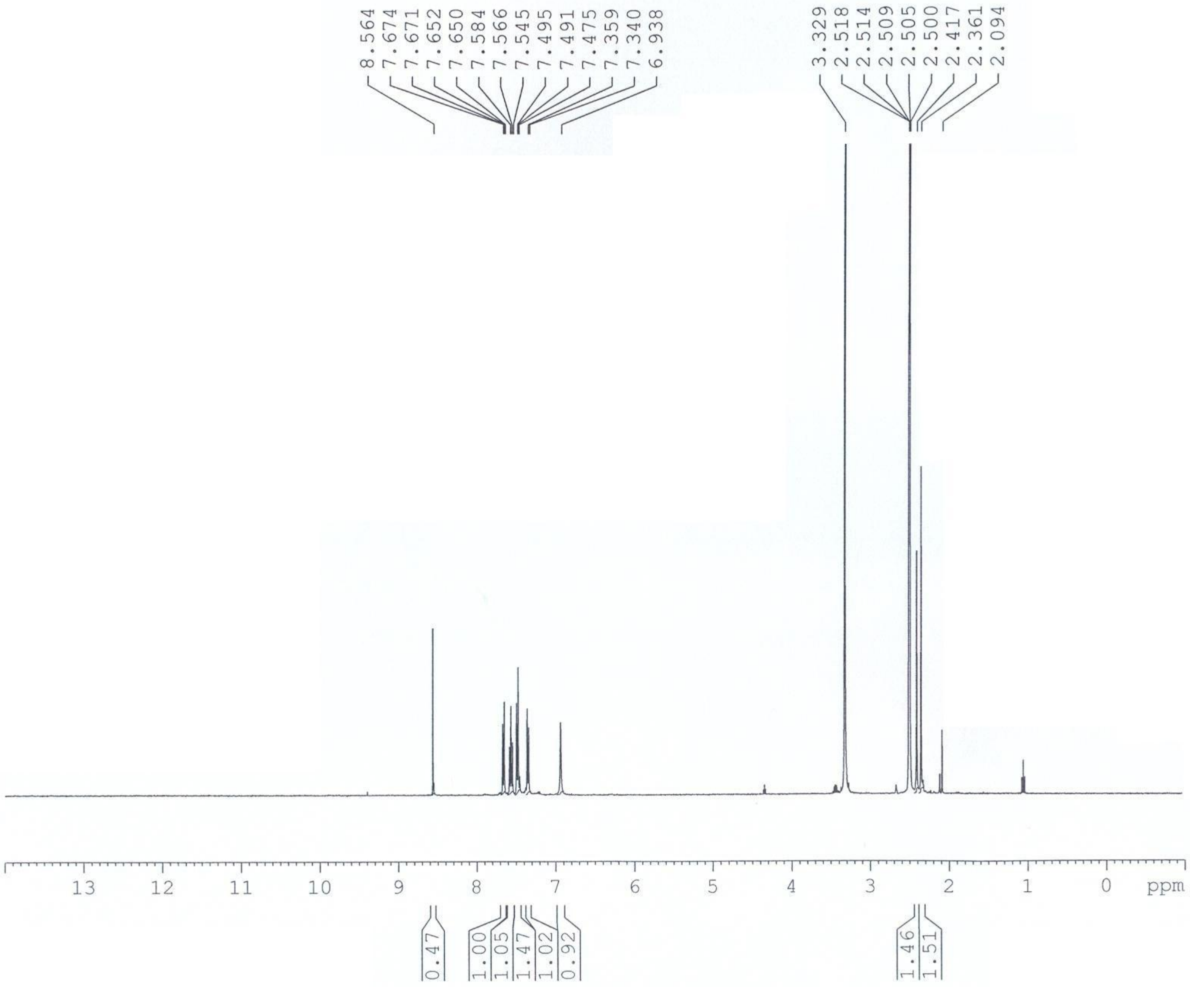

Compound 12L

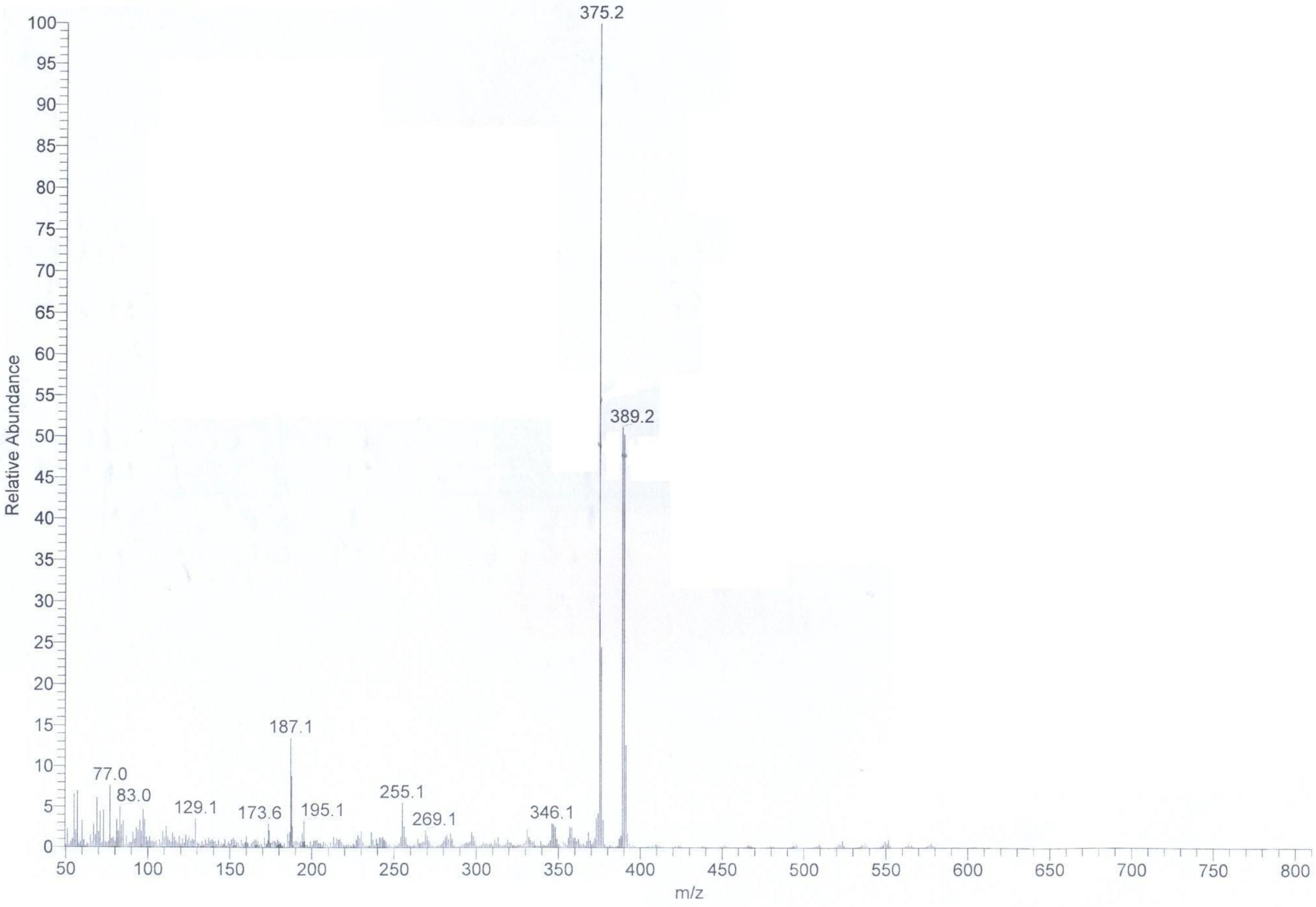

Compound 12L

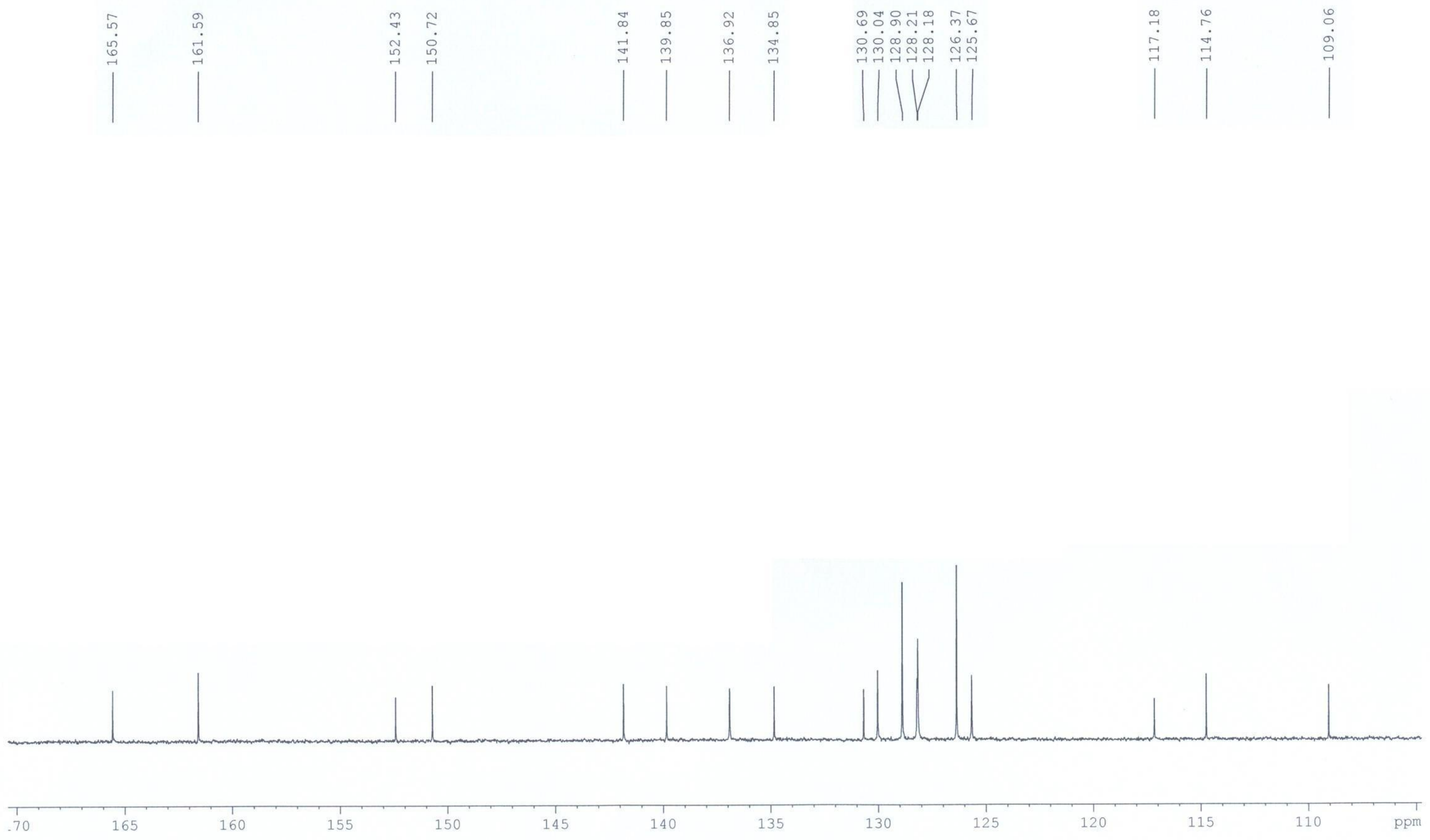

Compound 12L

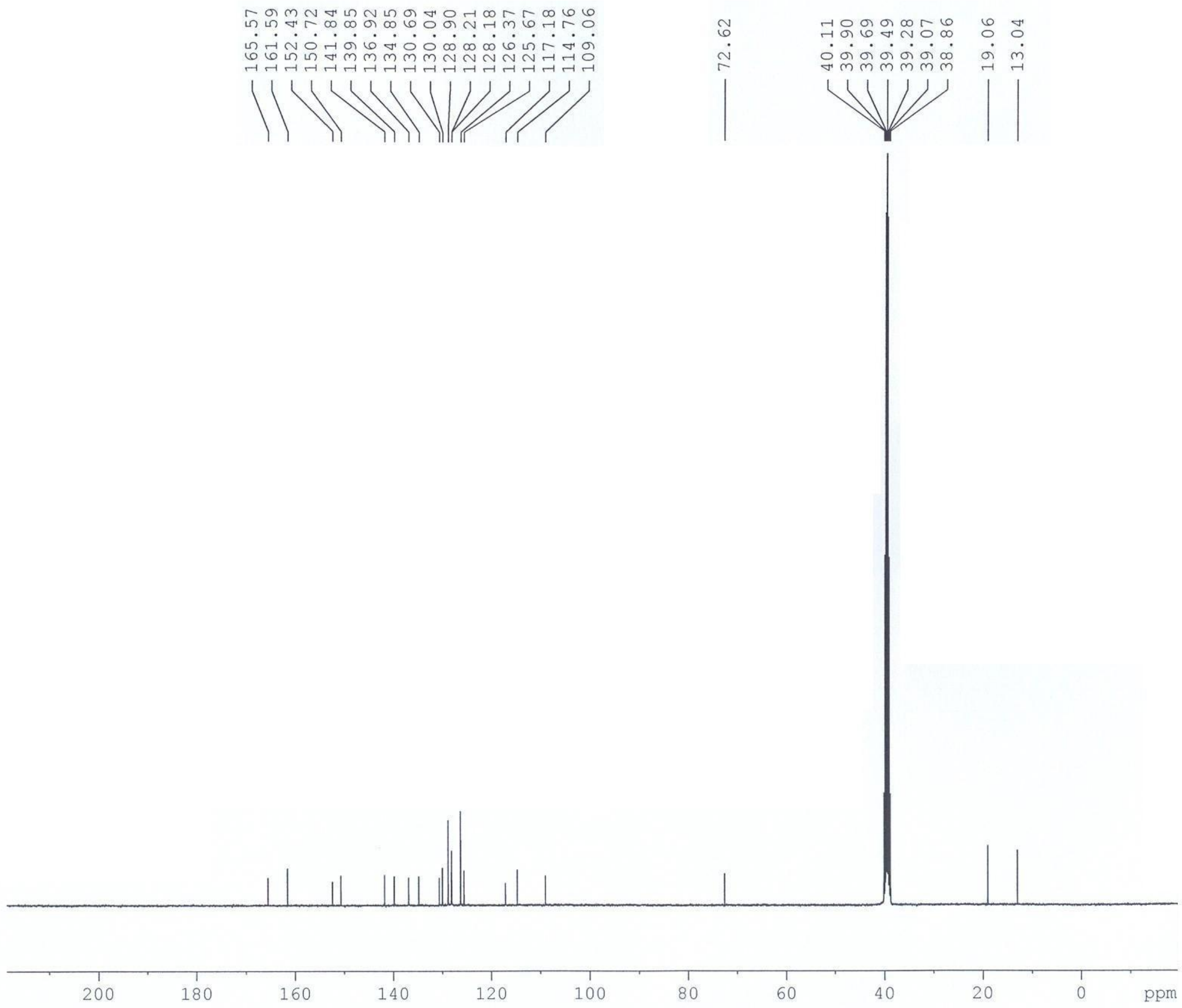

Compound 12L

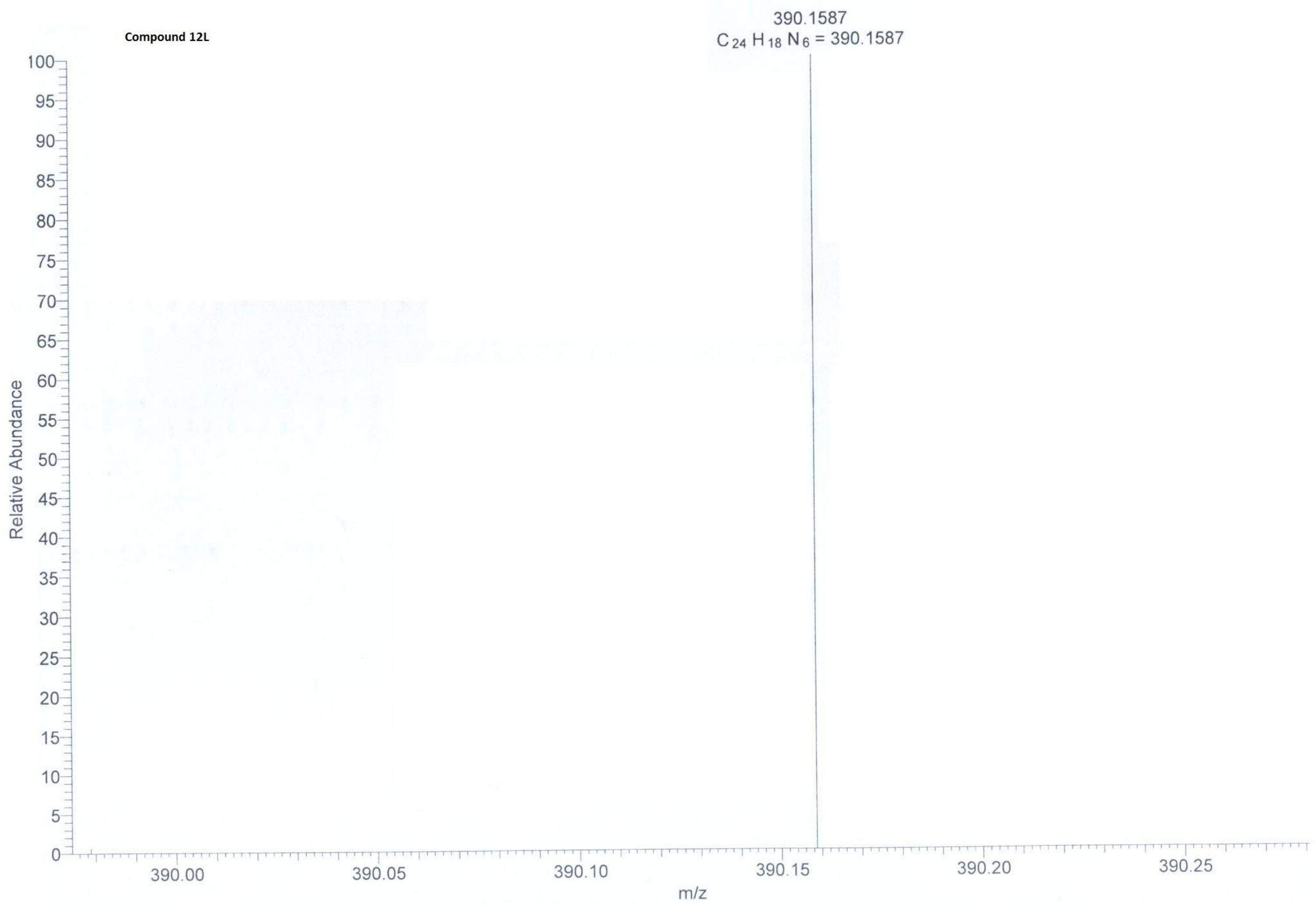

Compound 12L

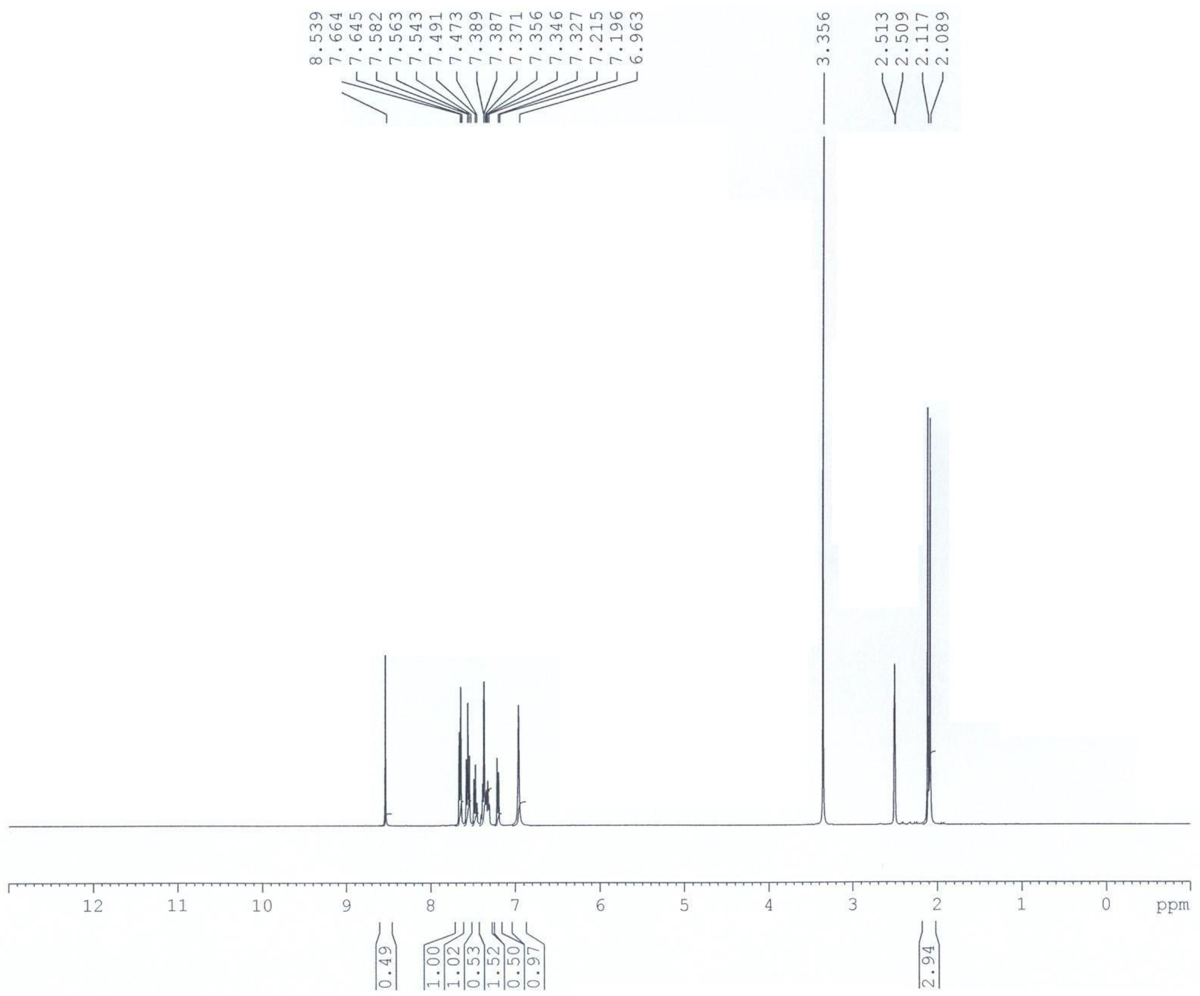

Compound 12L

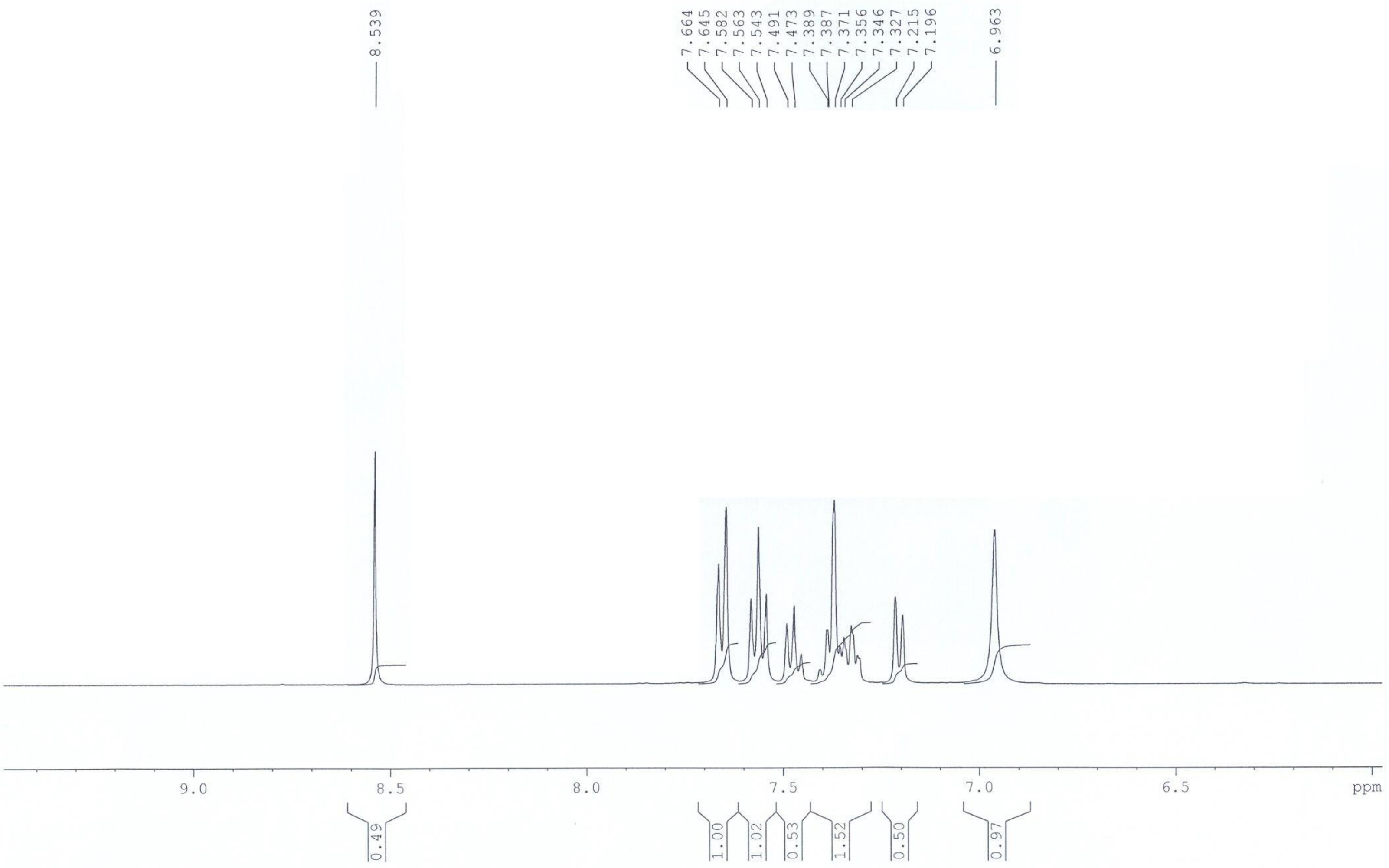

Compound 12m

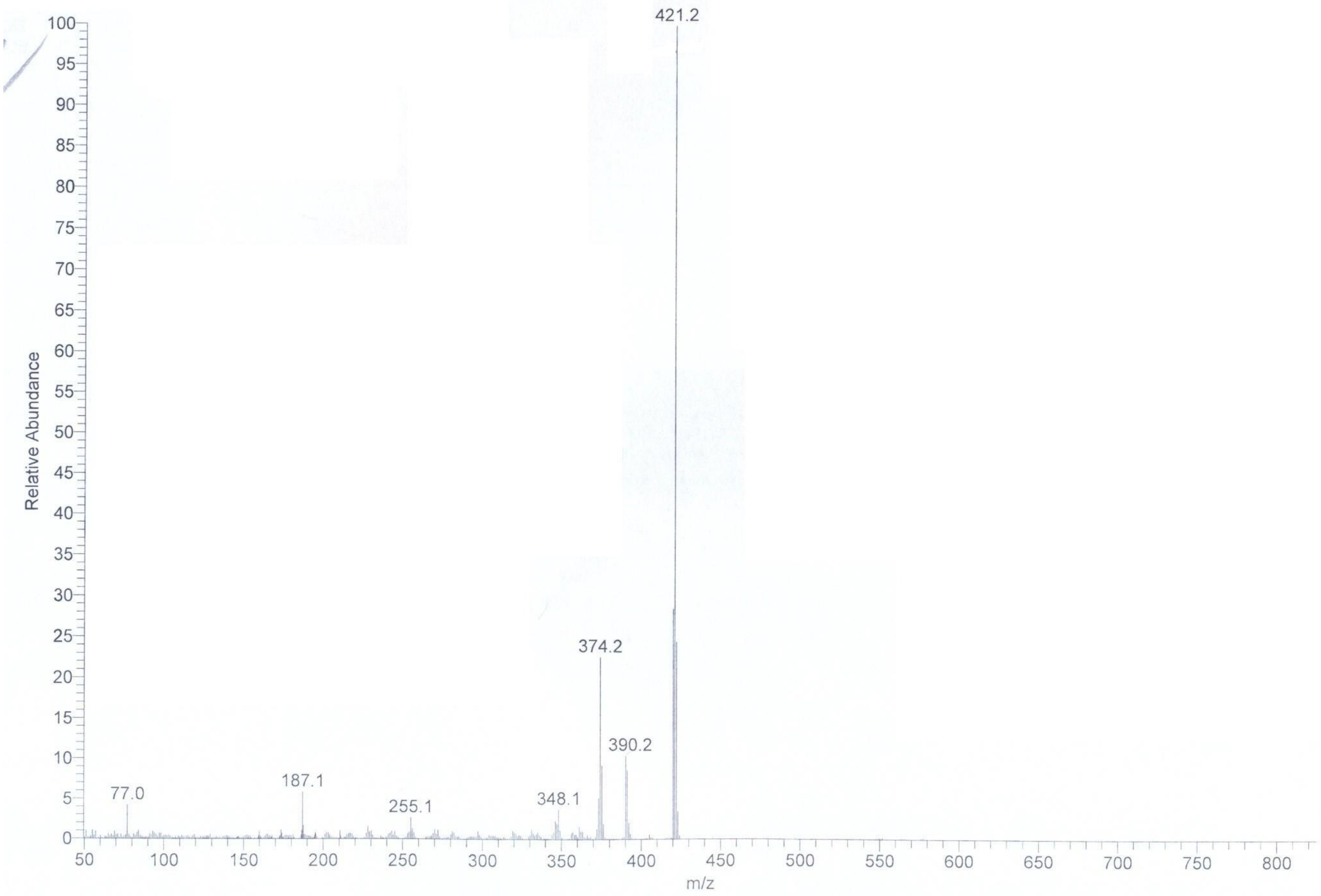

Agilent Technologies

Compound 12m

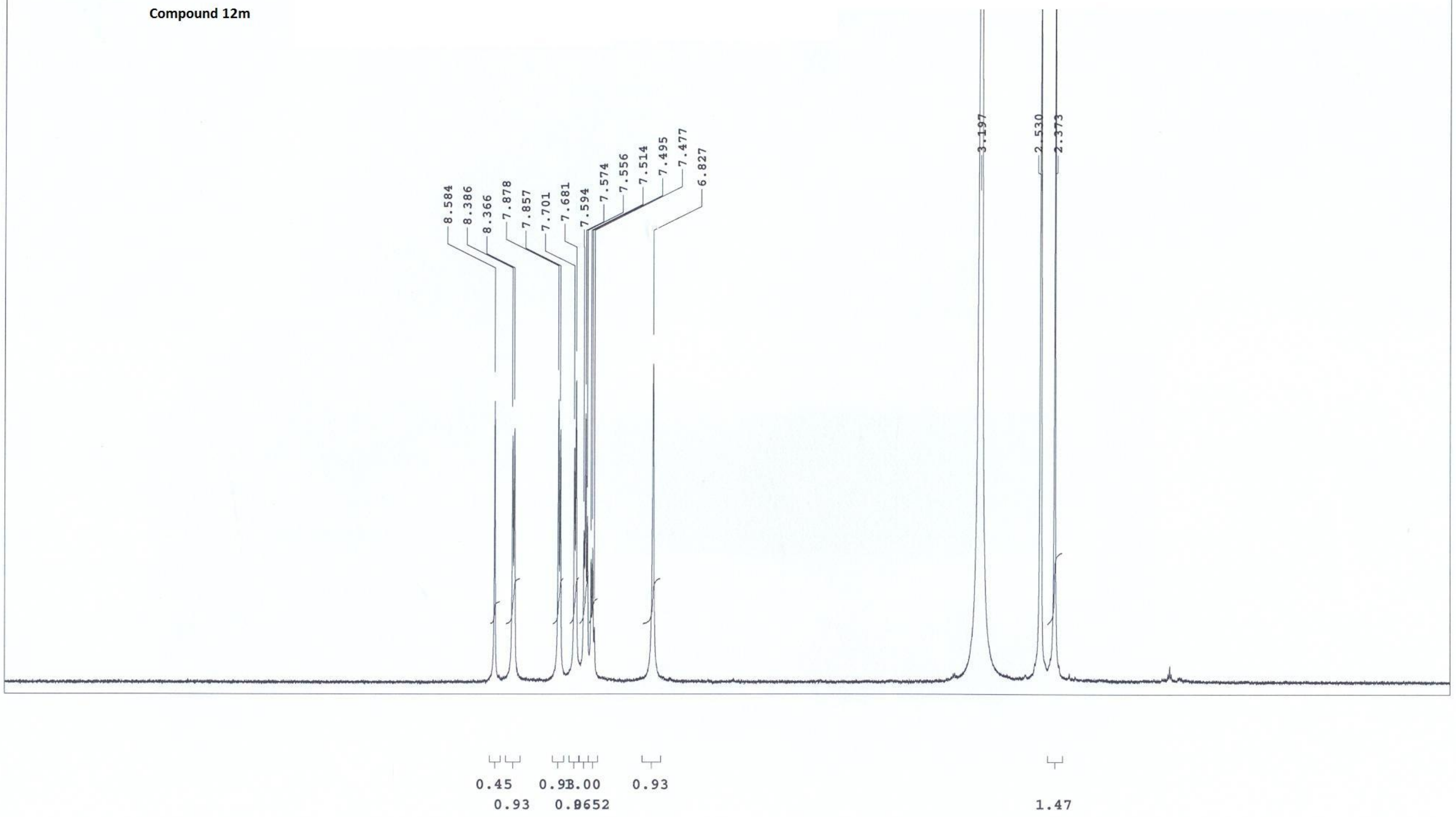

Compound 12m

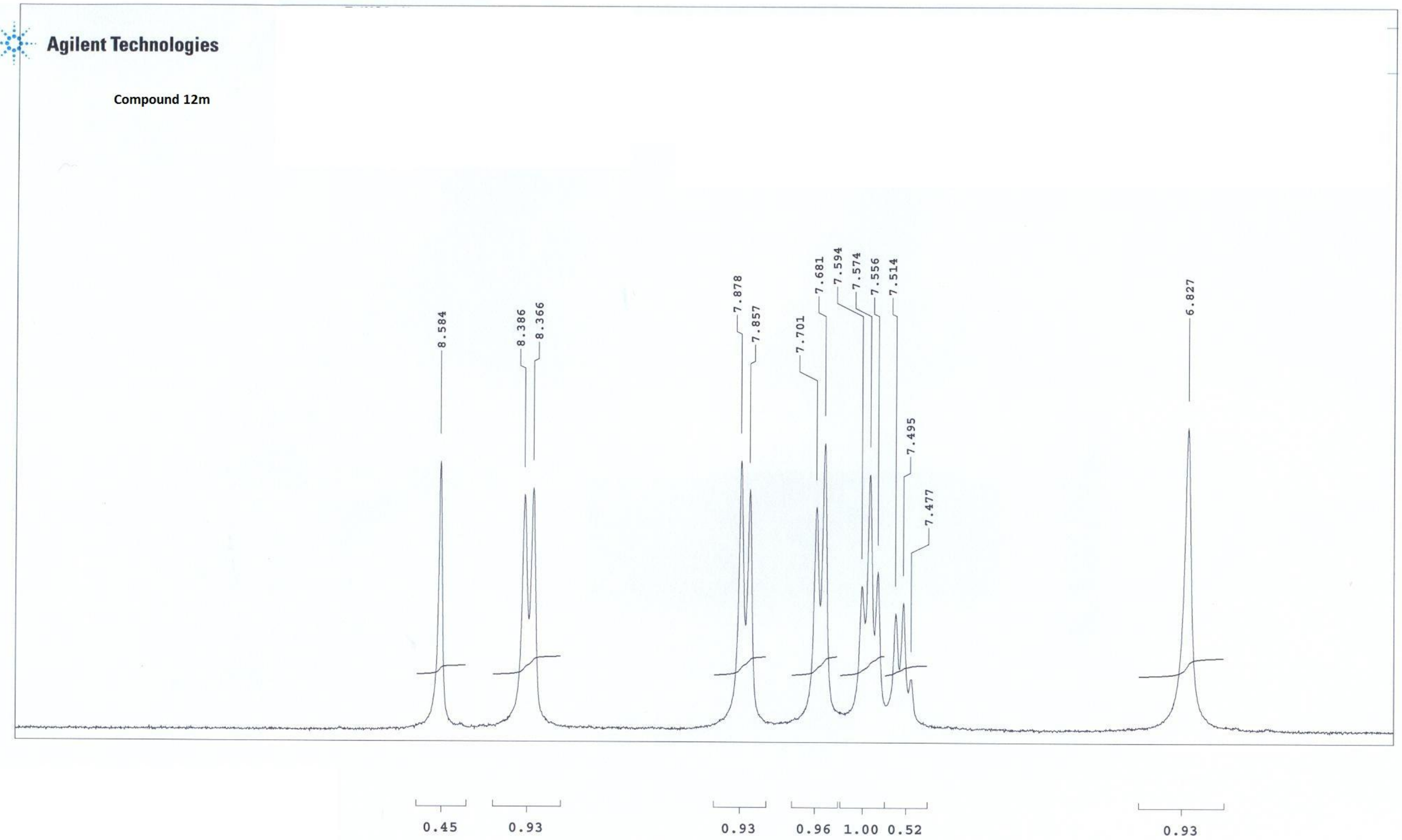

Agilent Technologies

Compound 12m

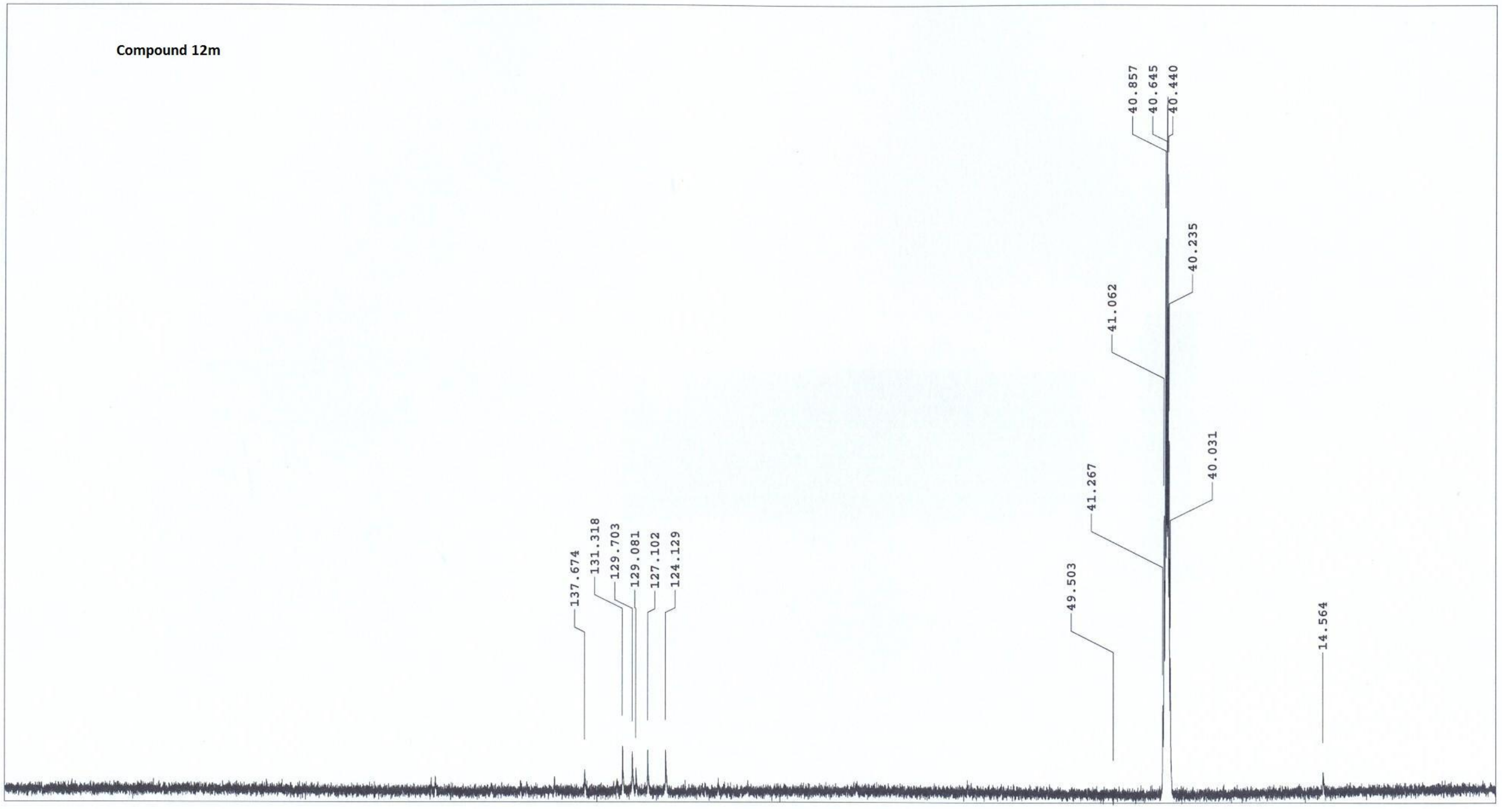

Agilent Technologies

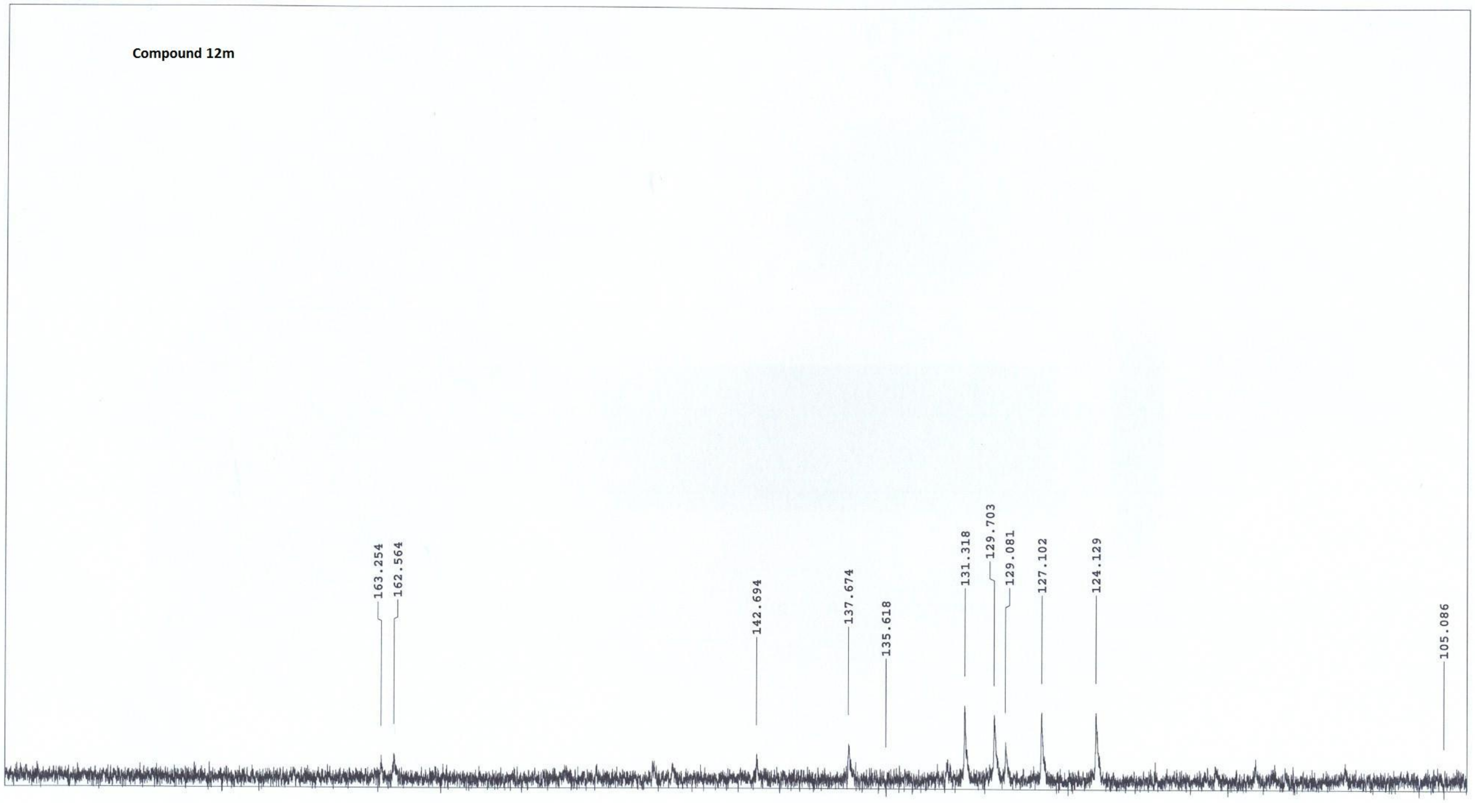

Compound 12m

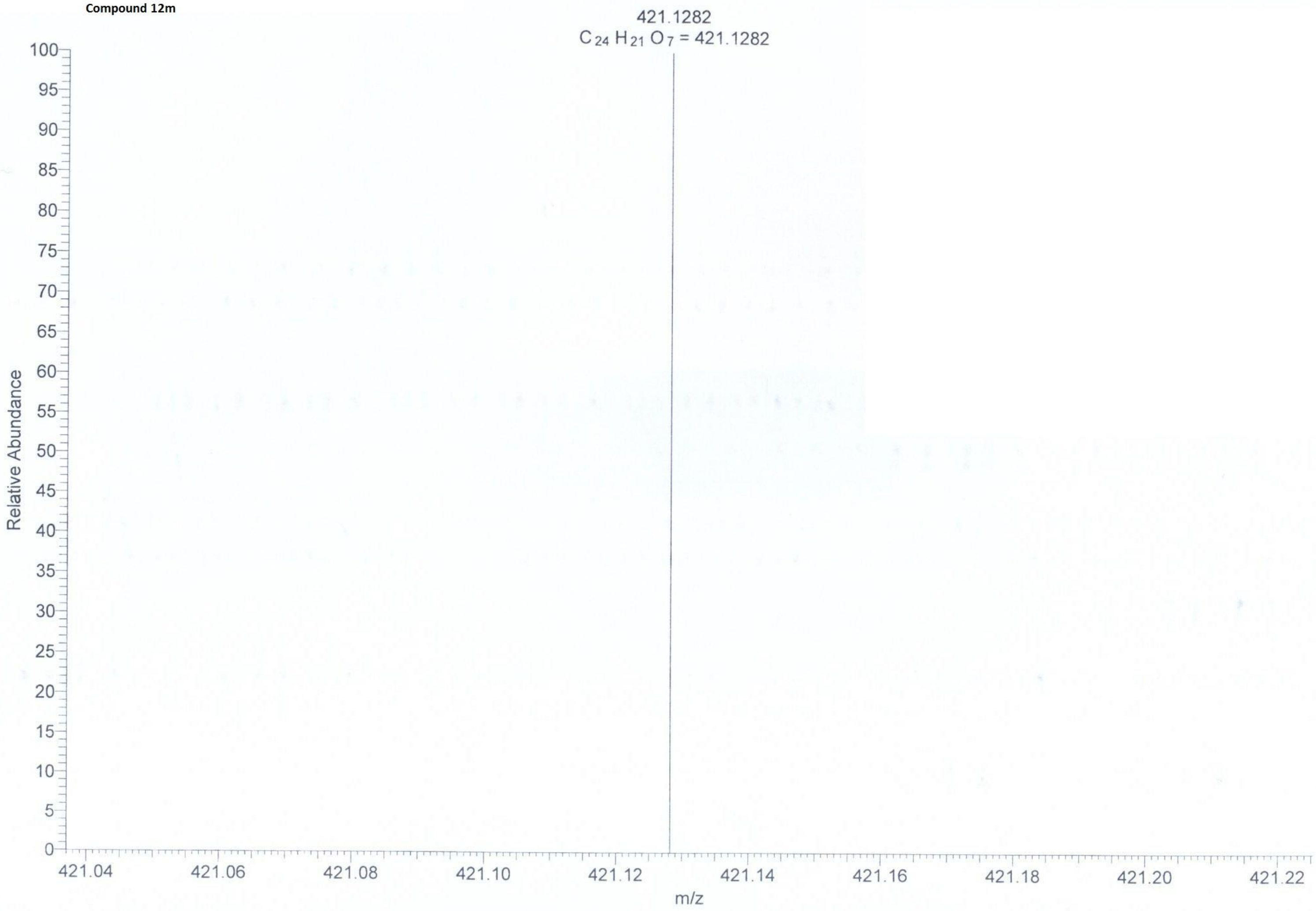

Compound 12n

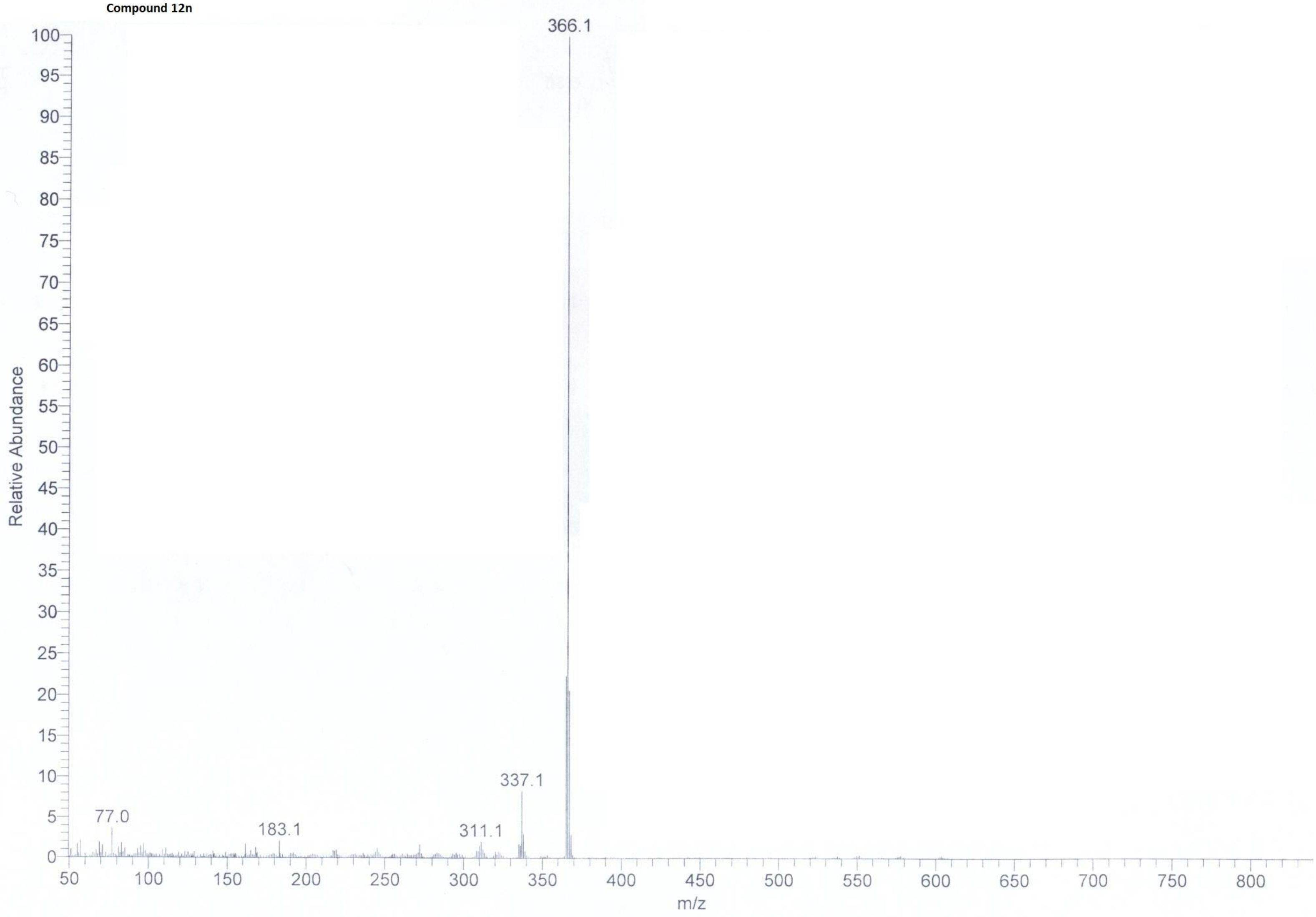

Compound 12n

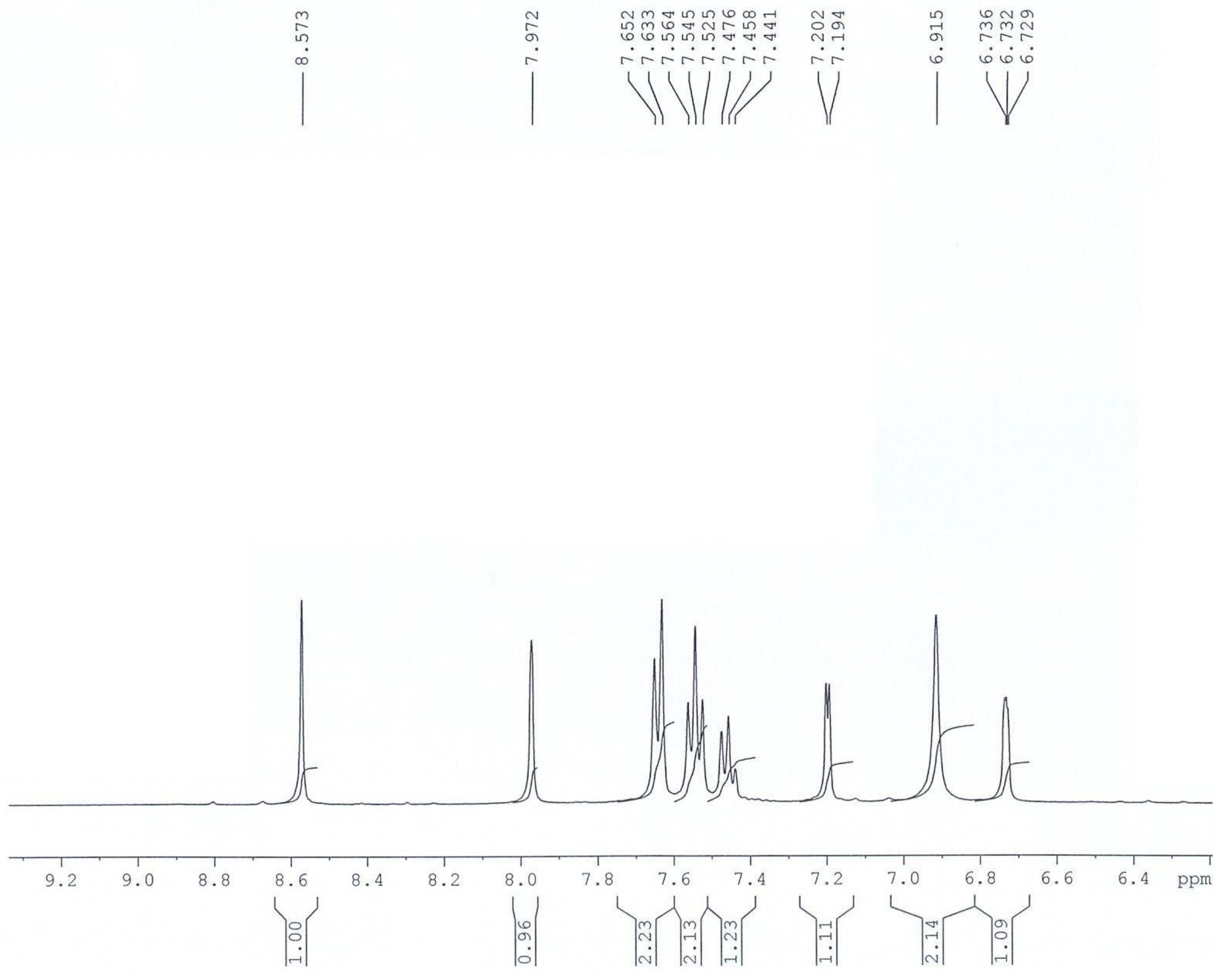

Compound 12n

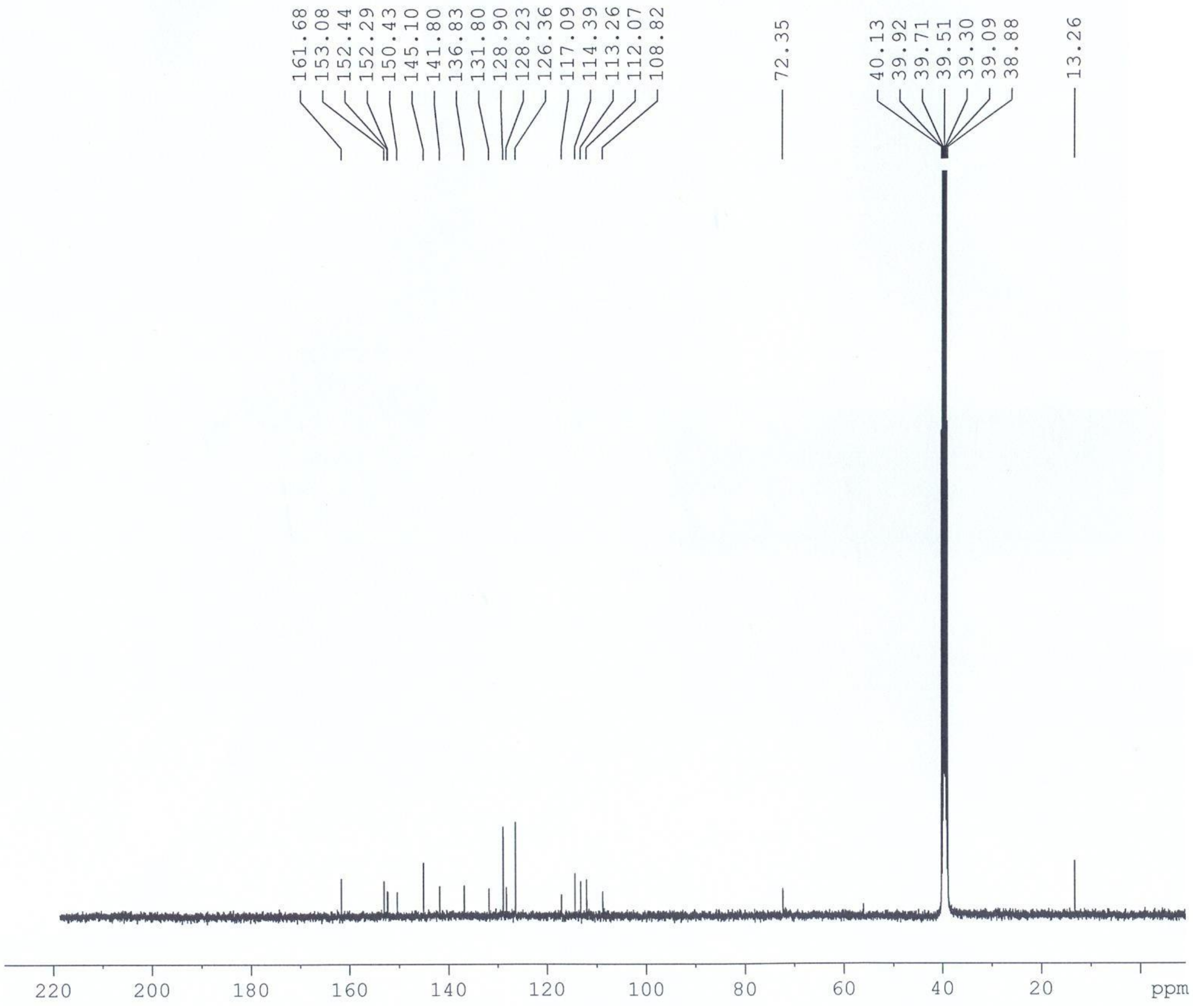

Compound 12n

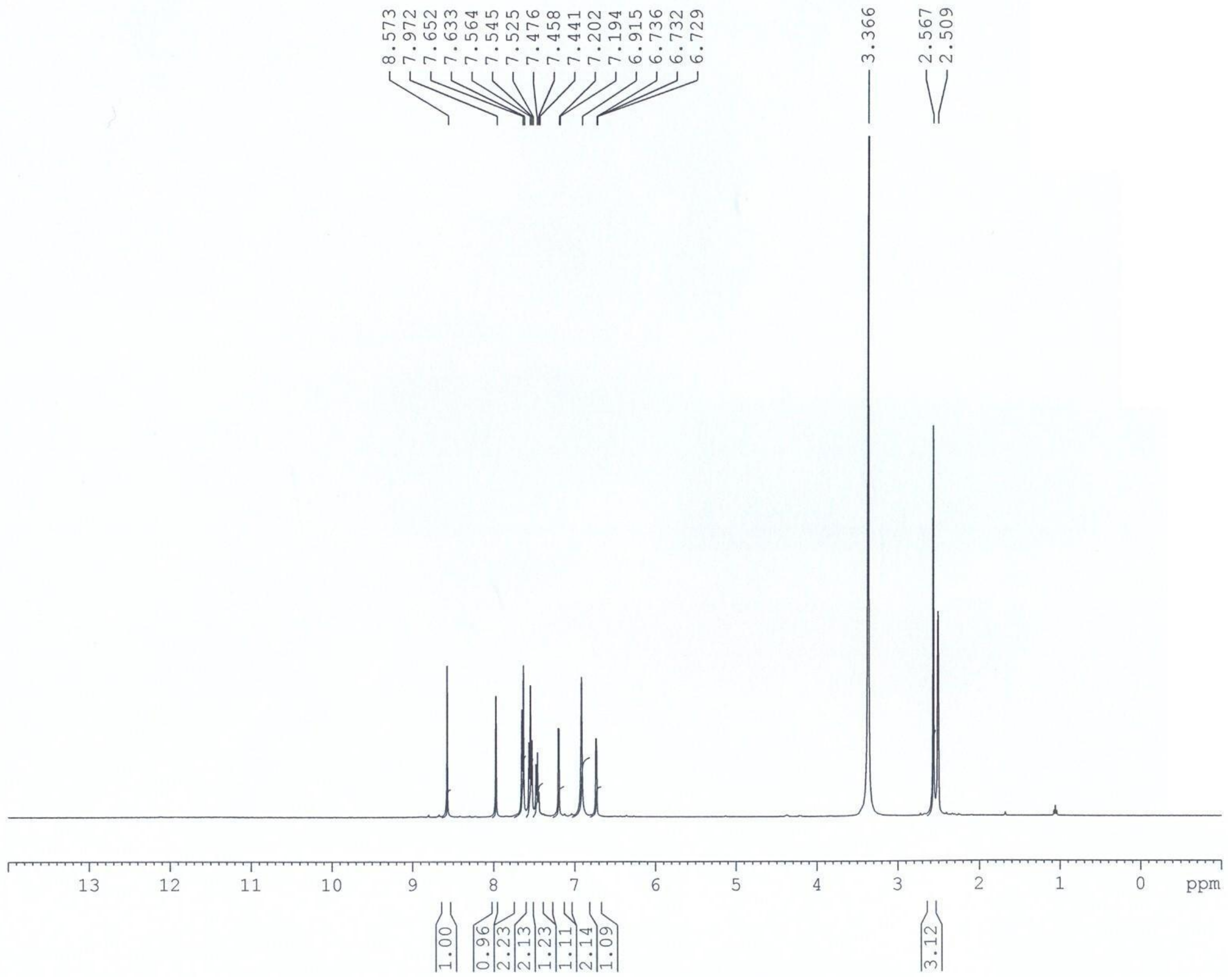

Compound 12n

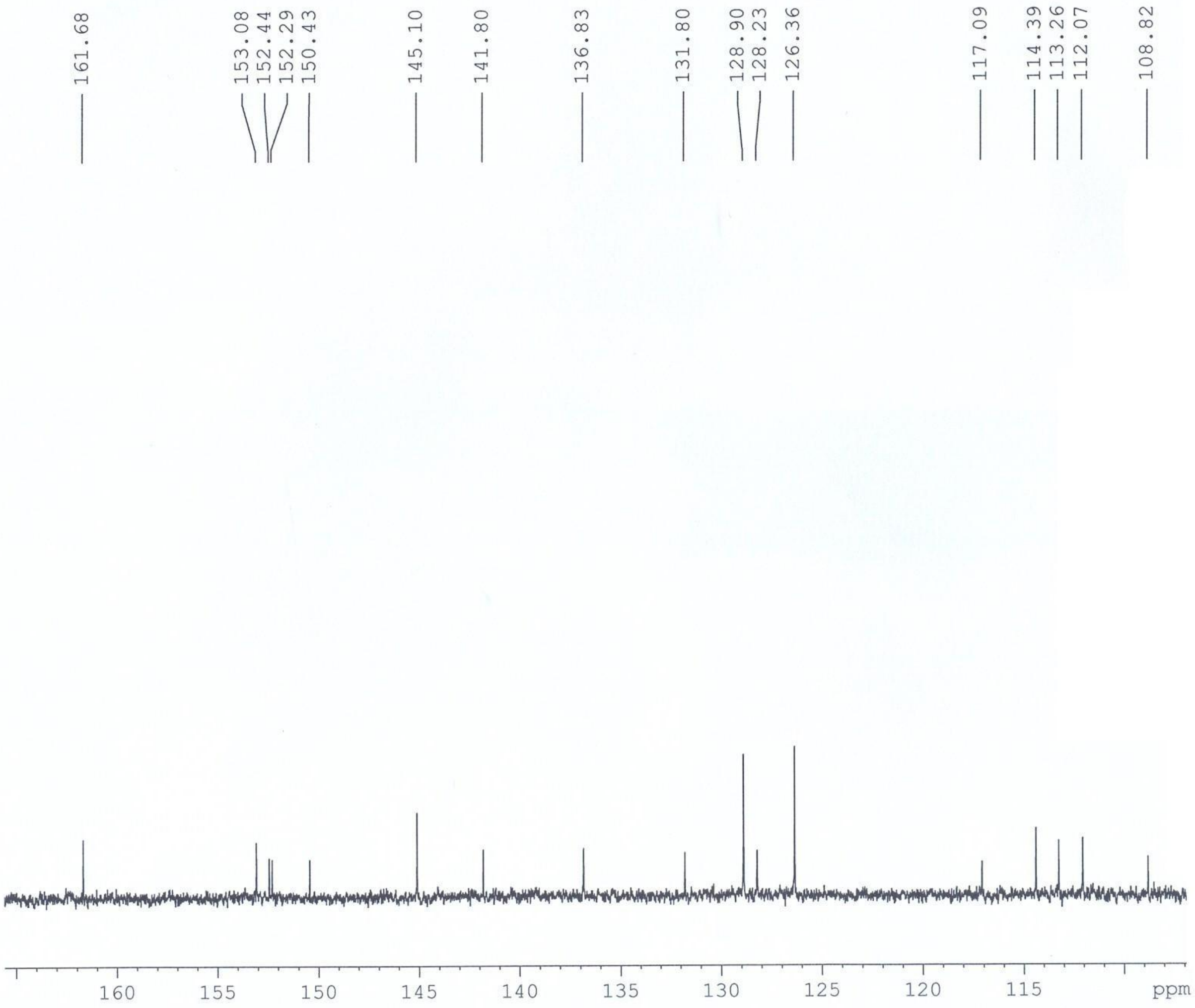

Compound 12n

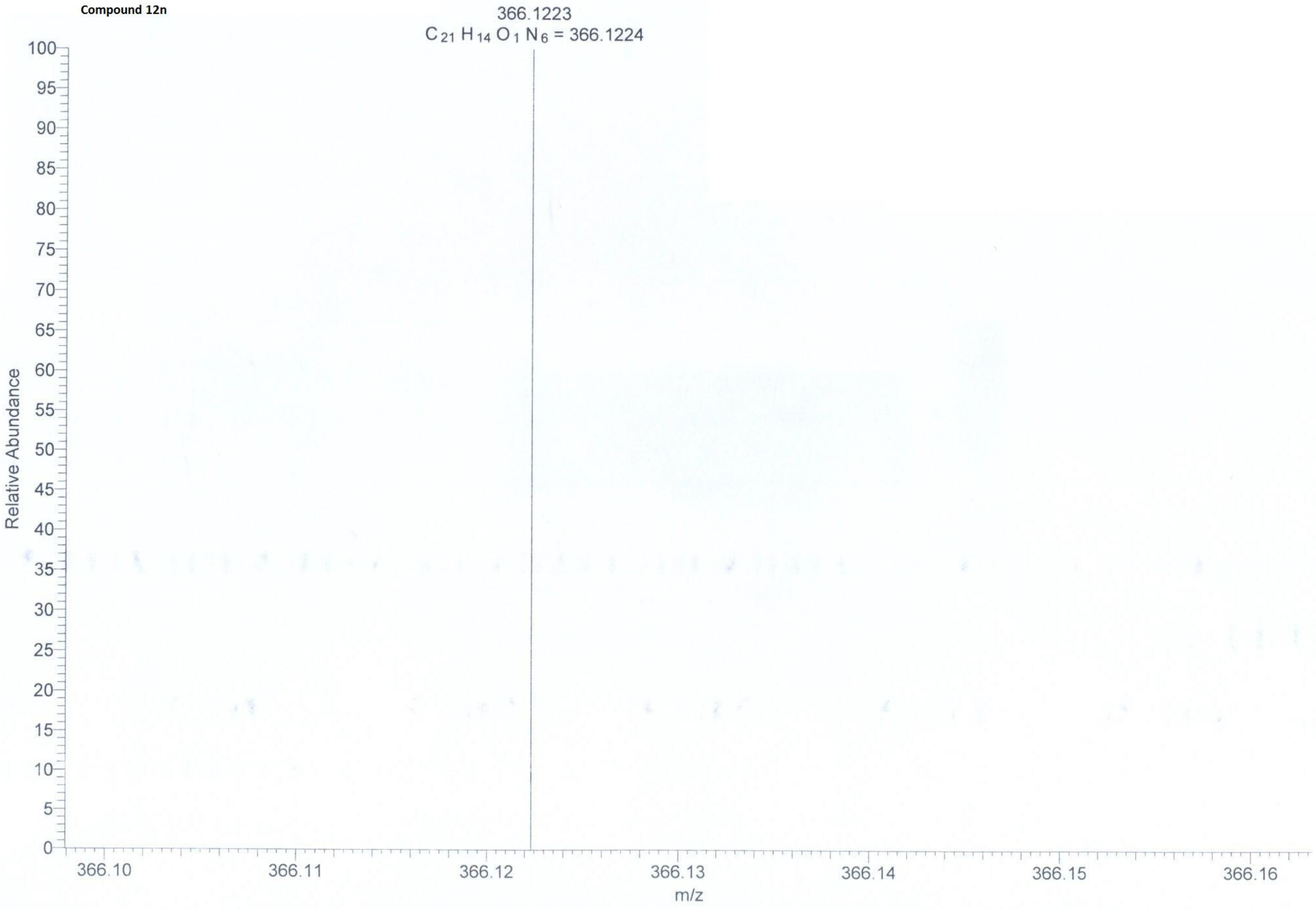

Supplement: Supplementary file 1 [file molecules-22-02114-s001.pdf]
